# Reductive evolution in *Streptococcus agalactiae* and the emergence of a host adapted lineage

**DOI:** 10.1186/1471-2164-14-252

**Published:** 2013-04-15

**Authors:** Isabelle Rosinski-Chupin, Elisabeth Sauvage, Barbara Mairey, Sophie Mangenot, Laurence Ma, Violette Da Cunha, Christophe Rusniok, Christiane Bouchier, Valérie Barbe, Philippe Glaser

**Affiliations:** 1 Unité de Biologie des Bactéries Pathogènes à Gram Positif, 28 rue du Docteur Roux, Paris, Cedex 15, 75724, France; 2 CNRS UMR 3525, Paris, France; 3 CEA/IG/Genoscope, Evry, France; 4 Genomic Platform, Institut Pasteur, Paris, France; 5 Unité de Biologie des Bactéries Intracellulaires, Institut Pasteur, Paris, France

**Keywords:** *Streptococcus agalactiae*, Host-adaptation, Non-homologous recombination, Gene inactivation, Virulence

## Abstract

**Background:**

During host specialization, inactivation of genes whose function is no more required is favored by changes in selective constraints and evolutionary bottlenecks. The Gram positive bacteria *Streptococcus agalactiae* (also called GBS), responsible for septicemia and meningitis in neonates also emerged during the seventies as a cause of severe epidemics in fish farms. To decipher the genetic basis for the emergence of these highly virulent GBS strains and of their adaptation to fish, we have analyzed the genomic sequence of seven strains isolated from fish and other poikilotherms.

**Results:**

Comparative analysis shows that the two groups of GBS strains responsible for fish epidemic diseases are only distantly related. While strains belonging to the clonal complex 7 cannot be distinguished from their human CC7 counterparts according to their gene content, strains belonging to the ST260-261 types probably diverged a long time ago. In this lineage, specialization to the fish host was correlated with a massive gene inactivation and broad changes in gene expression. We took advantage of the low level of sequence divergence between GBS strains and of the emergence of sublineages to reconstruct the different steps involved in this process. Non-homologous recombination was found to have played a major role in the genome erosion.

**Conclusions:**

Our results show that the early phase of genome reduction during host specialization mostly involves accumulation of small and likely reversible indels, followed by a second evolutionary step marked by a higher frequency of large deletions.

## Background

Comparative genomics of strains belonging to species with a large spectrum of hosts, such as *Staphylococcus aureus*, have highlighted two main evolutionary trends linked to the adaptation to a new host: acquisition of new functions through lateral gene transfer facilitating colonization of new niches and gene loss associated with the host specialization [1-3]. These trends were also recognized when considering the emergence of highly virulent host-specialized pathogens from bacterial species with a broader host range. For instance massive gene losses were associated to the emergence of human or equine pathogens such as *Salmonella enterica* sv Typhi and Paratyphi, *Bordetella pertussis* and *parapertussis*_*h*om_ and *Burkholderia mallei* that respectively derive from *Salmonella* Typhimurium*, Bordetella bronchiseptica* and *Burkholderia pseudomallei*[4-9]. During these transitions, it was postulated that gene inactivation and deletions were probably favored by genetic drift and evolutionary bottlenecks. In line with this model the host-specialized pathogens often showed a much higher number of insertions sequences (IS) than their parental strains and IS expansion was proposed to be largely responsible for gene deletions and genome rearrangements observed in these species [10].

*Streptococcus agalactiae* also referred to as Group B streptococcus (GBS) is a Gram-positive bacterium that has emerged as a leading cause of neonatal infections during the sixties and represents an increasing cause of infections in the elderly and in adults with underlying diseases [11-13]. As a commensal it colonizes the digestive and genitourinary tracts of up to 30% of the human adult population [14]. However, *S. agalactiae* was initially described as an animal pathogen causing mastitis in ruminant [15]. Since the 70’s, *S. agalactiae* was found to be responsible for epidemic events of invasive diseases in fish farms, leading to a mortality of up to 30% [16-18]. Cases of infection were also reported for other aquatic poikilotherms such as frogs [19] and aquatic mammals such as dolphins [20]. How GBS is able to adapt to its different hosts remains poorly understood. The genetic diversity of GBS populations has been studied using different methods including multilocus sequence typing (MLST) [21], which led to the recognition of different clonal complexes (CC). Some of these clonal complexes display host preference. For instance, CC67 is essentially associated with the bovine host and the hypervirulent sequence type (ST) 17 strains are mainly isolated from humans. However incidentally strains belonging to human-associated clonal complexes are also isolated from bovines [22] suggesting that relationships between clonal complexes and host specificity are not so strict. Further analysis of the complete genome sequences of eight isolates of human origin and one of bovine origin has highlighted the composite organization of *S. agalactiae* genomes with a conserved backbone (representing the core genome of the species) and a dispensable genome composed of genomic islands that are highly variable between the different strains [23-26].

The ST261 strain 2-22 (or ATCC 51487) was initially isolated as responsible for several epidemics in fish farms in Israel [27,28]. This strain, which showed a restricted metabolic pattern, thermosensitivity and lack of ß hemolytic and CAMP activities was first classified as a different species, *Streptococcus difficile,* but proved later to be a genuine serotype Ib *S. agalactiae* strain [17]. *S. agalactiae* strains were repeatedly isolated from fish infections and found to cluster into two main groups [20,29]. The first group corresponds to strains belonging to the clonal complex 7, also displaying strains isolated from human and bovine hosts. The other strains share two or more common MLST alleles with strain 2-22 and are classified in ST246, 257, 259, 260, 552 and 553. As ST261 strains, these STs were until now never isolated from humans [29]. In addition, a third group of strains belonging to clonal complex 283 has recently been described in fish and humans [29]. The draft genome sequences of one ST260 strain, strain STIR-CD-17, and two ST7 strains, strain ZQ0910 and GD201008-001, respectively isolated from disease outbreaks affecting farmed tilapia in Honduras and Nile Tilapia in China were recently published [30-32].

To decipher the phylogenomic relationships between *S. agalactiae* strains isolated from fish or other poikilotherm animals and strains isolated from human or bovine, we analyzed the genomic sequence of seven isolates belonging to ST260-261 and ST6-7, including the strain 2-22. Comparative analysis confirmed that the two groups of GBS strains responsible for fish epidemic diseases are distantly related. We found that adaptation to fish does not involve any specific function compared to human CC7 isolates. Conversely, specialization to the fish host of the ST260-261 strains was associated with massive gene inactivation and deep remodeling of metabolic and regulatory networks that we also characterized at the transcriptome level. This genome reduction likely occurred through RecA independent recombination.

## Results and discussion

### Fish ST7 strains are closely related to human strains

**Figure 1:**
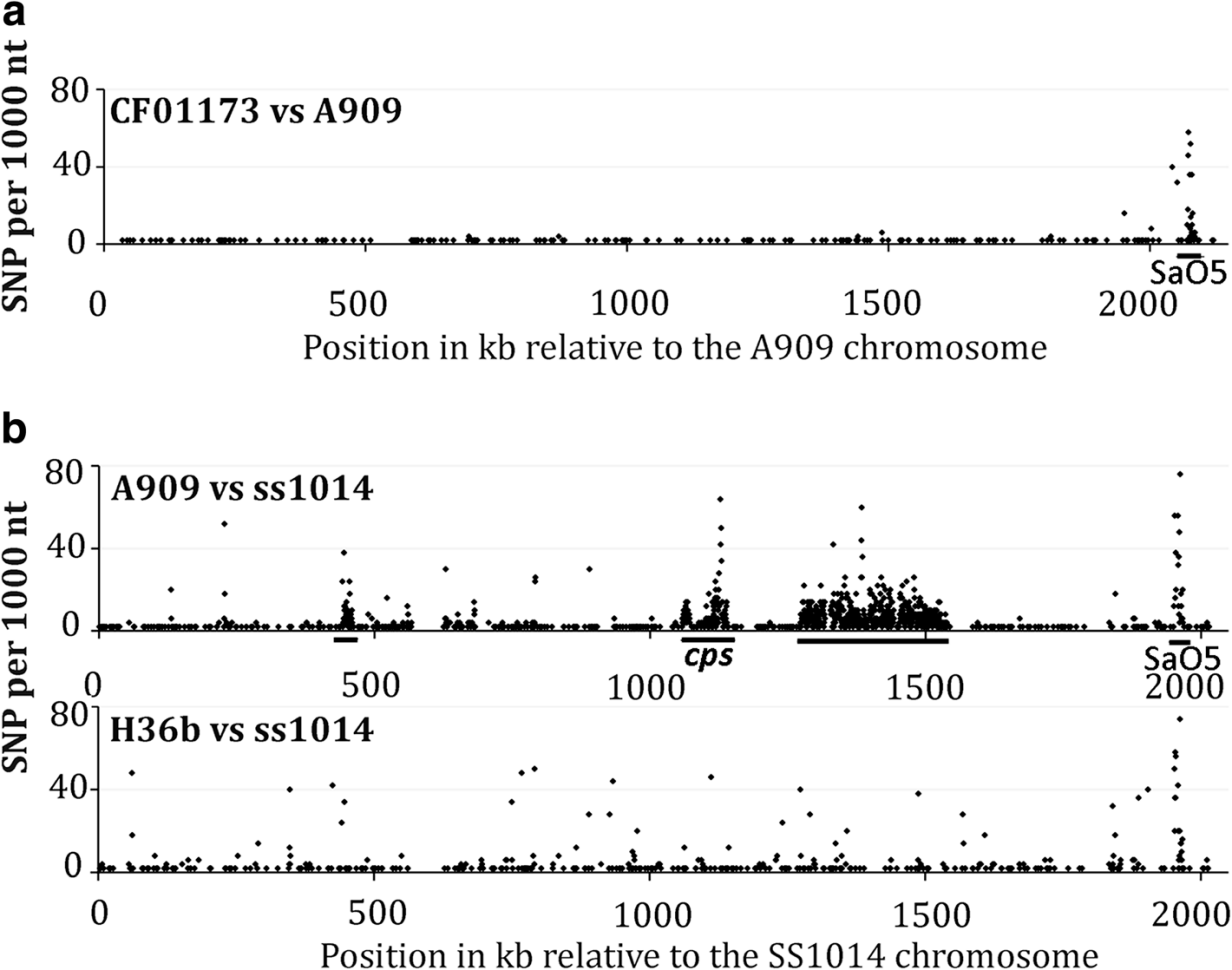
**SNP distribution along the genome sequences of CC7 strains.** (**a**) The number of SNPs (y axis) per 1000 nt in strain CF01173 (ST7) versus strain A909 (ST7) is plotted according to the position of the corresponding fragment on the strain A909 genome (x axis). The goodness of fit to a uniform distribution was tested using a Chi square test on 19 class intervals of 100,000 nt, excluding regions of prophage SaO5, and differences in SNP distribution were found to be not significant (p = 0.20). (**b**) The numbers of SNPs (y axis) per 1000 nt in strains A909 (ST7) and H36B (ST6) versus strain SS1014 (ST6) are plotted according to the position of the corresponding fragment on the strain SS1014 genome (x axis). Regions of higher polymorphism, including the capsule locus (*cps*) and prophage SaO5, are underlined.

We first compared the gene content of *S. agalactiae* strains isolated from fish but grouped into the same CC as the human strain A909 by sequencing the genome of strains CF01173 and SS1014, isolated in USA and UK respectively (Table 1). Whole genome sequence comparison showed that strain CF01173 differed from strain A909 by only 389 SNPs (Additional file 1: Table S2). CF01173 was also closely related to the recently described strains ZQ0910 and GD201008-001 [31,32] isolated from diseased fish in China, which differ by only 105 and 100 SNPs respectively. In contrast the ST6 strain SS1014 was more distant (3484 SNPs), and proved to be closer to the ST6 strain H36B (689 SNPs) isolated from human [26] (Additional file 1: Table S1 and Table S2). Analysis of SNP distributions along the genome sequence showed a uniform distribution when strains of the same ST were compared (Figure 1), with a mean polymorphism of 0.1-0.2 SNP per 1000 nt except in the sequence of an inserted prophage. In contrast alignment of ST6 versus ST7 strains revealed a mosaic pattern of regions of low polymorphism (0.1-0.2 SNP per 1000 nt) interrupted by several regions of higher polymorphism (5-15 SNP per 1000 nt on average) that were probably gained by recombination with distantly related GBS strains, as previously suggested [33]. One of these regions corresponds to the capsule locus that encodes a serotype Ia capsule in ST7 strains and a serotype Ib capsule in ST6 strains. Therefore ST6 and ST7 strains probably shared a common ancestor and recently diverged by recombination with other GBS strains, modifying the capsular serotype between both ST.

**Table 1:** Characteristics of the different strains used in the study

**Strain**	**Origin**	**Infection**	**MLST**	**Serotype**	**Geographical origin**	**Genome size (kbp)**	**Ref.**
**2-22**	trout	meningitis	ST261	Ib	Israel	1,839	[27]
**SS1218**	frog	-	ST261	Ib	Louisiana	1,797	[34]
**05-108A**	tilapia (*Oreochromis* sp.)	meningitis	ST260	Ib	Honduras	1,801	[35]
**90-503**	Hybrid striped bass	meningitis	ST260	Ib	Louisiana	1,753	[35]
**SS1219**	frog	-	ST260	Ib	Taiwan	1,798	[34]
**SS1014**	striped bass	-	ST6	Ib	USA	2,016	[34]
**CF01173**	trout	-	ST7	Ia	GB	2,027	[36]
**A909**	human	-	ST7	Ia	-	2,128	[26]

Gene content was similar between strains CF01173 and A909 except for 13 genes that were disrupted in CF01173 and five in A909 (Additional file 2: Table S3). Seven genes were specifically disrupted in strain SS1014. In addition, we found that the three ST7 strains CF01173, ZQ0910 and GD201008-001 isolated from fish shared one short genomic island that was absent from other GBS and probably resulted from lateral gene transfer. This genomic island encodes proteins 70-95% identical with proteins of *Streptococcus anginosus,* including a protein with a LPXTG-motif cell-wall anchor domain (GBS1173_1788). Sharing of this island in addition to the low number of SNPs between strains CF01173, ZQ0910 and GD201008-001 suggests that the three strains have a common recent ancestor that propagated worldwide. In contrast, emergence of the ST6 strain SS1014 can be considered as an independent event.

Overall, the low level of polymorphism with human strain indicates that CC7 strains infecting fish recently diverged from strains isolated in humans and bovines. Since at least two independent events of emergence were observed, this suggests that CC7 strains might be more amenable to fish colonization/infection than other GBS clonal complexes isolated from humans or bovines. In agreement with this hypothesis, it was recently shown that a *S. agalactiae* ST7 strain isolated from human was able to cause disease in Nile tilapia [37].

### ST260-261 strains form an independent lineage which underwent reductive evolution

**Figure 2:**
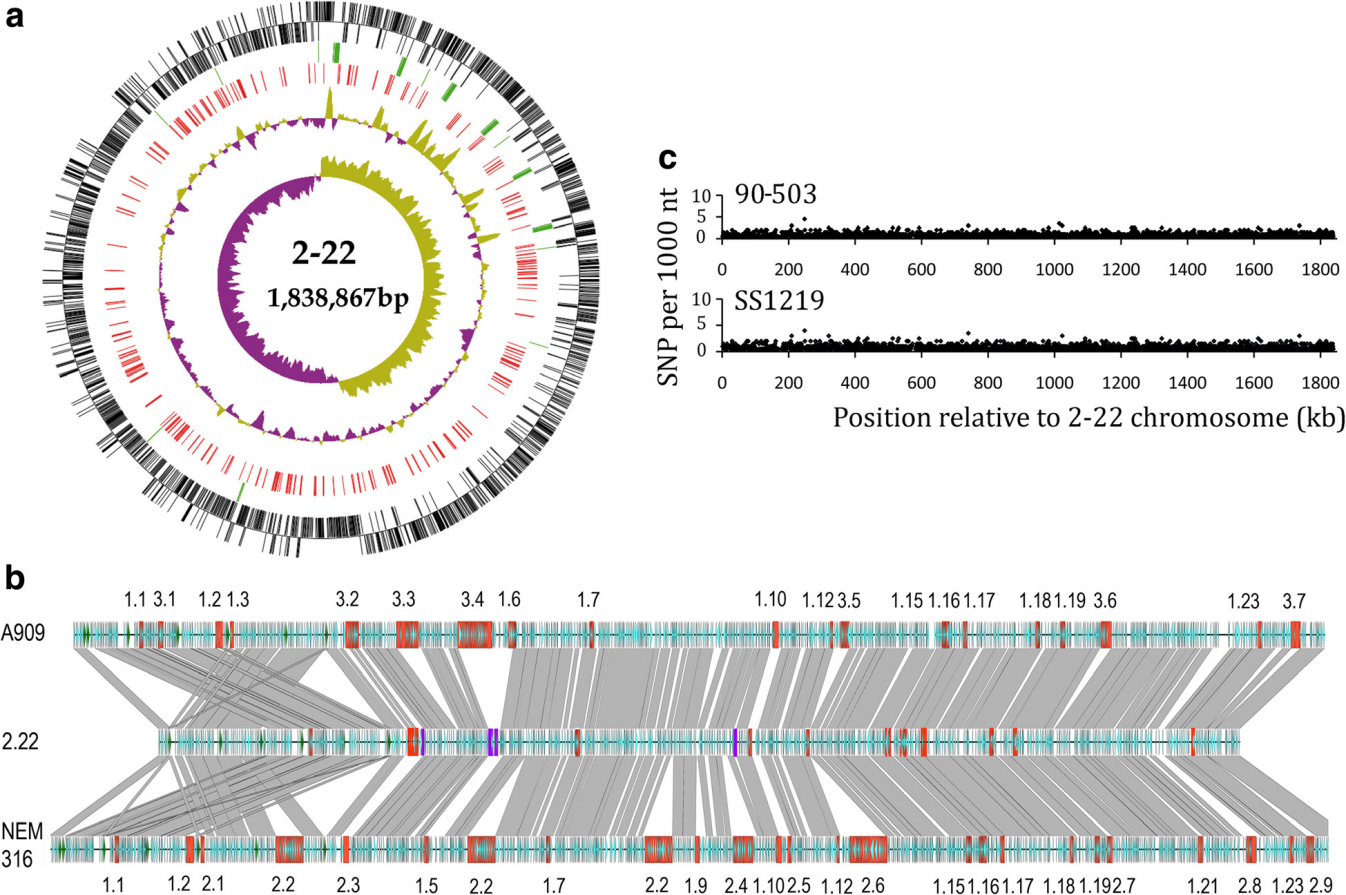
**Genome map of the *S. agalactiae *strain 2-22 and genome comparisons with other GBS strains.** (**a**) Circular genome map of *S. agalactiae* strain 2-22 showing gene positions and orientations. From the outside: circle 1: protein coding genes on the + and - strands; circle 2, in green: genes for rRNA and tRNA; circle 3, in red: pseudogenes; circle 4: %GC plot; circle 5: GC skew [(GC)/(G + C)]. (**b**) Genome sequence alignment with *S. agalactiae* strains A909 and NEM316. Genomic islands characterized in GBS human strains are indicated in red and numbered according to [26] while genomic islands specific to strain 2-22 are in violet. CDS are shown in blue and rDNA genes in green. (**c**) SNP distribution along the genome sequences of ST260-261 strains. The number of SNPs as determined by nucmer [40] (y axis) per 1000 nt in the ST260 strains 90-503 and SS1219 versus strain 2-22 is plotted according to the position of the corresponding fragment on the strain 2-22 chromosome (x axis). The goodness of fit to a uniform distribution was tested using a Chi square test on 36 class intervals of 50000 nt and differences in SNP distribution were found to be not significant (p = 0.20).

To explore the phylogenomics relationships between the second group of strains isolated from fish, and CC7 strains, we sequenced the genome of five strains belonging to ST260 and ST261. While the genomes of four of these strains were obtained as draft sequences, the genome of strain 2-22 was sequenced to completion. Strain 2-22 genome consists of a single circular chromosome of 1,838,867 bp (Figure 2a); this is 10 to 25% smaller than the genome sizes of other sequenced GBS strains, which range from 2,065 kb (ST17 human strain COH1) to 2,456 kb (ST67 bovine strain FSLS3-026). The G + C content (35.5%) is similar to that of other GBS strains. Compared to human GBS genomes, the genome of strain 2-22 lacks one rDNA cluster and 9 tRNA genes (71 tRNA genes and 6 rDNA clusters versus 80 and 7 respectively in other GBS genomes). The deletion of this rDNA cluster is associated with the translocation of a 150 kb genomic region probably caused by recombination between flanking ribosomal RNA operons, as also observed in *Salmonella* Typhi [38,39]. Except for this region the genome of strain 2-22 is syntenic to the genomes of human strains (Figure 2b). The four other strains have a similar genome size as strain 2-22 (Table 1).

Whole-genome sequence comparison of the five strains showed that they clustered into two distinct subgroups correlating with the MLST classification (Additional file 1: Table S2). The ST261 strain SS1218 isolated from frog in Louisiana differed from the strain 2-22 by only 30 SNPs. Strains 90-503 isolated in Louisiana in 1990 and 05-108A isolated in 2005 in Honduras, with 49 SNPs can be considered as variants of the same clone. Strain SS1219 isolated from frog in Taiwan diverged from the 2 former ST260 strains by 130 SNPs. On average, the ST260 strains showed 3,100 SNPs with ST261 strains. ST260-261 strains were also related to the ST552 strain Sa20-06 (3,400 and 1,700 SNPs respectively). In contrast, ST260-261 displayed 15,000 SNP with CC7 strains. Analysis of the SNP distribution along the genome sequences of ST260 and ST261 strains revealed a uniform pattern of 3 SNPs per kb (Figure 2c), suggesting that no recombination occurred in this lineage.

**Figure 3:**
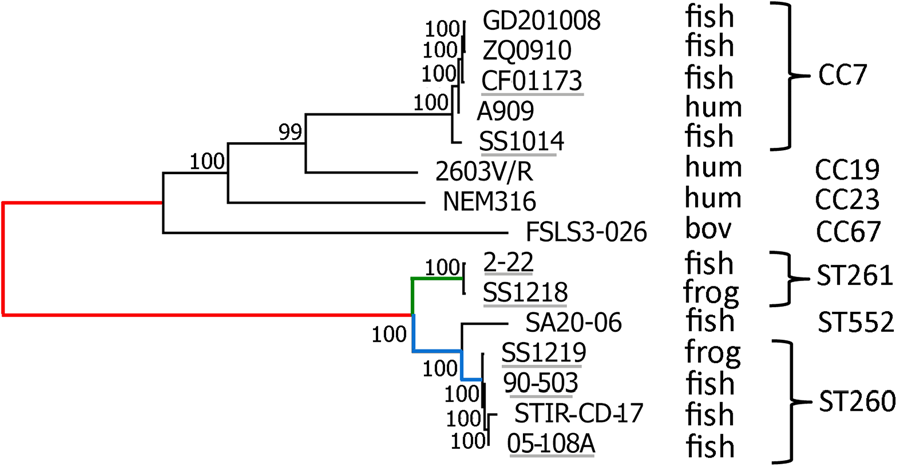
**Phylogenetic relationships between *S. agalactiae *strains isolated from fish/frog, bovine and human.** A maximum likelihood phylogenetic tree was constructed using the MEGA 5.04 software [41] based on 25,597 concatenated SNPs from the core genome of GBS strains (1.34 Mb) of which regions recombined between ST6 and ST7 strains have been removed. The bootstrap values resulting from 1000 replicates are indicated. Strains sequenced in this work are underlined. The tree branches that are further analyzed in Figure 4 are colored (red: N_fbh_-A_260+261_ branch; green: A_260+261_-A_261_ branch; blue: A_260+261_-A_260_ branch). A phylogenetic analysis using the Neighbor-Joining method gave the same topology for the tree (not shown).

A phylogenetic analysis of strains of human, bovine and fish or frog origins confirmed that ST260-261-552 strains constitute a distinct lineage. Separation of this lineage from other *S. agalactiae* clonal complexes, including CC7 strains, was probably ancient, pre-dating the separation between the three strains of human origin (NEM316, A909 and 2606V/R) (Figure 3). While the comparison of the whole genome sequence showed the same mean identity between ST260-261 strains and the three human strains, a higher proportion of nucleotides was found to align with the genome of strain A909 (97.15%) than with other GBS genomes (2603 V/R: 95.64%, NEM316: 95.43%, FSLS3-026: 92.27%) (Additional file 1: Table S1).

The low proportion of nucleotides that did not align with the genomes of human GBS strains suggested that ST260-261 strains encode only few specific functions. Indeed, only four genomic islands were characterized in strain 2-22, that represented 25 kb in total (in violet on Figure 2b). However these four regions essentially contained pseudogenes and one of them was absent from the ST260 strains. We also identified in ST260-261 strains eleven to twenty copies of ISSag1, an insertion sequence previously described in the genomes of human isolates and other streptococci [42] (Additional file 3: Table S4). In addition, the genomes of ST260-261 strains contained 10 regions categorized as genomic islands in human *S. agalactiae* genomes [26], but shared by most GBS characterized so far (shown for strain 2-22 in Figure 2b). They did not contain any Integrative and Conjugative Elements (ICE) or prophages and only two inactivated copies of integrases were identified. They also lacked two mobile genetic elements encoding important virulence loci: the cis-mobilizable element encoding the major surface antigen Alpha like protein/Rib [43] and the composite transposon coding for the C5A peptidase and the laminin binding protein [44]. In total the difference in genomic island content between ST260-261 strains and GBS strains isolated from humans or bovines accounted for about 70-80% of the genome reduction (210-230 kb). An interesting exception was the genomic island 3.2 that was previously described only in strains A909 (ST7) and H36B (ST6) and therefore is also shared by CC7 strains isolated from fish. This conservation is in favor of a role in fish colonization. This GI encodes two phosphotransferase systems (PTS) for galactitol, sugar ABC transporters and genes for galactose utilization (*GBS222_0398-0414* in strain 2-22) (Additional file 4: Figure S1). Sharing of this genomic island explained the higher number of aligned nucleotides between strains 2-22 and A909.

Genome annotation predicted 1547, 1568, 1560 and 1569 protein coding genes for strains 2-22, 90-503, SS1219 and 05-108A respectively, which is significantly less compared to 2096 in NEM316, 1990 in A909 and 2135 in 2603V/R. Furthermore, 190-220 pseudogenes were identified in each strain (Additional file 5: Table S5) compared to 27 to 41 in the human strains. This revealed a massive reduction of the functional genome during the time-course of adaptation to fish in the ST260-261 lineage.

### Reductive evolution is an ongoing process in the ST260-261 lineage

**Figure 4:**
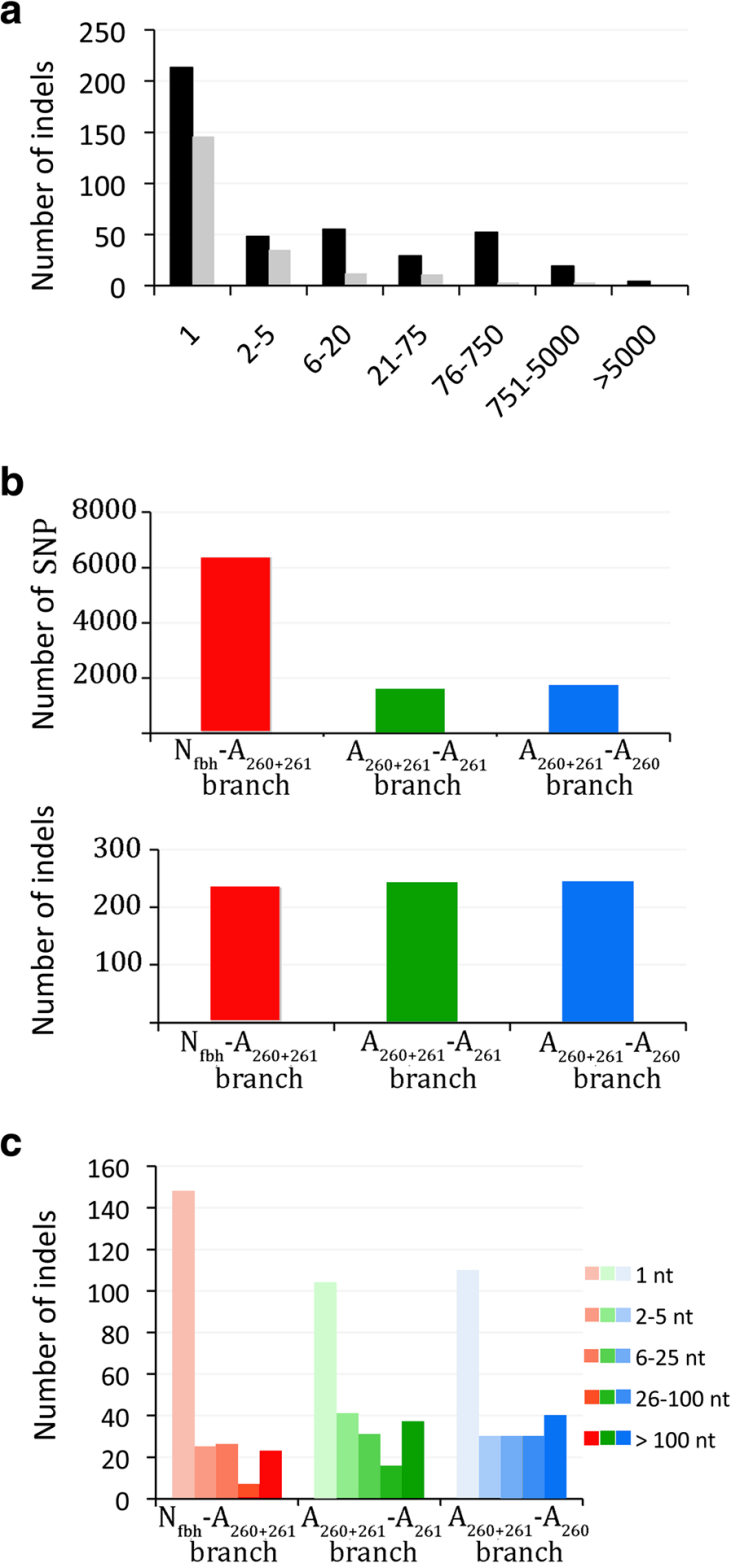
**Ongoing reductive evolution in the ST260-261 lineage.** (**a**) Distribution of insertions/deletions between strains 2-22 and A909, according to their sizes in nucleotides. Black bars correspond to sequences missing in the 2-22 strain compared to A909 and gray bars to sequences missing in A909 compared to strain 2-22. Only indels mapped in the core genome sequences are represented. **b**) Comparison of the number of indels and SNPs that occurred before (N_fbh_-A_260+261_ branch) and after sublineage separation (A_260+261_-A_261_ and A_260+261_-A_260_ branches). Indels and nucleotide changes relative to the A909 sequence were deduced from the comparison of the 15 strains shown in Figure 3. The mutation events were positioned on the phylogenetic tree according to a parcimonious approach. (**c**) Size distribution of the deletion events identified on the N_fbh_-A_260+261,_ A_260+261_-A_261_ and A_260+261_-A_260_ branches respectively. Colors (red, green and blue) correspond to the branch colors of the tree presented in Figure 3.

To get more insights into the evolution of the ST260-261 lineage, we further analyzed the nucleotide changes leading to gene disruption and genome size reduction compared to human strains. Strain A909, belonging to CC7, was used as a reference to compare the genome sequences of the five ST260-261 strains. We also aligned the genomes of two human and one bovine isolates to identify nucleotide changes specific to the fish lineage. Genomic islands and insertion sequences were excluded from the analysis as well as thirty sequences whose evolution involved both insertions and deletions of nucleotides. In these conditions, sequence alignment between strain 2-22 and A909 genomes revealed 621 simple insertion/deletion events ranging from 1 to 10,095 nt (Figure 4a). Using the sequences of human strains as outgroups, we found that, among these indels, 160 corresponded to deletions and 60 to insertions that specifically occurred in the ST261 sublineage, after ST260-261 divergence. The mean size of deletions largely exceeded that of insertions since only 300 nucleotides were gained while 47,000 were lost. Deletions, insertions and nucleotide replacements were respectively responsible for 76, 21 and 19 gene disruptions. In addition, 15% of the indels led to minor protein size modifications (less than 20%) and their consequences on protein functions were more difficult to predict. Finally 30% of the indels were characterized in intergenic regions. Altogether mutations leading to gain or loss of nucleotides represented approximately 12% of total mutations after the divergence from ST260 strains and constituted the major process of gene disruption. This evolution was not specific to the ST261 sublineage as a similar number of insertions and deletions occurred in the ST260 sublineage accounting for 86 gene disruptions (Figure 4b).

In addition, 235 indels and 6363 SNPs relative to strain A909 sequence were common to the five ST260-261 strains but were not observed in A909 *vs* other GBS strains. Forty per cent of these indels were intragenic and led to 91 gene disruptions (Figure 4c). Finally, 34 genes disrupted by a small indel in one sublineage were deleted in the other, suggesting that gene disruption could be a preliminary step to gene loss and genome reduction by secondary longer deletions. As a consequence some loci were observed under different states of decay in the two sublineages. This is the case for instance for the *cyl* locus, the *CRISPR2* locus and the *pil2* pilus locus (Additional file 4: Figure S2). Furthermore, the average size of the indels was found to increase following the divergence of the two sublineages (Figure 4c) compared to the common branch. Therefore, while the process of reductive evolution was already evident in the ancestor of the ST260-261 strains, it was even more pronounced after divergence of the two sublineages.

To better evaluate the specificity of the evolutionary process occurring in ST260-261 strains, we compared the core genome sequence of strains A909 and NEM316, two strains of human origin differing by approximately 11,000 SNP. We detected 314 indels, which represents half the number of indels in the 2-22 *vs* A909 comparison. These indels were essentially short and as much as 80% of them occurred in intergenic regions while less than 4% led to gene inactivations. Therefore, gain or loss of nucleotides was also frequent in other *S. agalactiae* strains, but most indels in coding sequences were probably eliminated by purifying selection. Fitting with this hypothesis, we observed one indel per 5-10 SNPs during the recent evolution of ST7 strains where purifying selection is probably not effective. A similar proportion of indels *vs* SNP was also reported for two strains of *S. enterica* sv Paratyphi [4].

**Figure 5:**
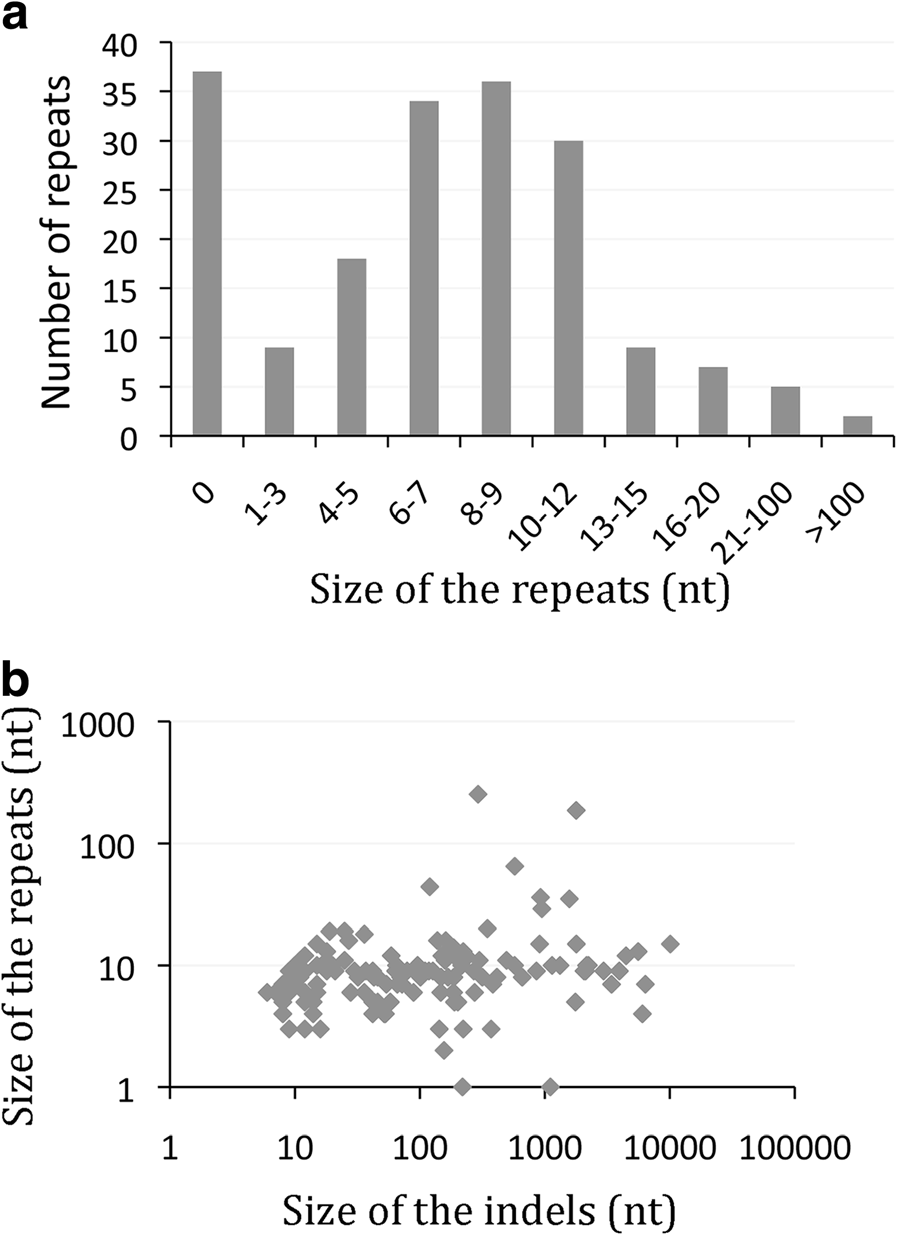
**Characteristics of the repeats identified at the extremities of insertions/deletions.** (**a**) Size distribution of the repeats flanking the 193 indels larger than 4 nucleotides identified in the genome alignment of strains 2-22 and A909. (**b**) Scatter plot (in log scale) showing the size of the indel in nucleotides (x axis) and the length of the flanking repeat in nucleotides (y axis) for the 193 indels larger than 4 nucleotides.

Finally, to gain further insights into the mechanism of the observed insertions and deletions, we analyzed the sequences flanking the indels in strain 2-22. Among the 358 indels of 1 nt, 210 (60%) occurred in homopolymeric tracts longer than four nucleotides and likely resulted from DNA polymerase slippage during replication. Interestingly, 136 of the 193 (70%) indels larger than four nucleotides apparently also involved recombination between repeated sequences. The median size of the repeats was 8 nucleotides, with 8 sequences larger than 20 nucleotides, the largest being 254 nt long (Figure 5a). Since the threshold of repeat length for RecA-dependent homologous recombination is 23-27 nt [45], most of the indels probably occurred by RecA independent recombination. It has been shown that, under laboratory conditions, the efficiency of illegitimate recombination is highly dependent on the size of the repeats [46] and inversely dependent on the distance between repeats [47-49]. Our results suggest that RecA independent recombination between repeats of moderate sizes may also lead to long deletions. Furthermore we did not find any evidence that deletions of long sequences may depend on longer repeats (Figure 5b). This may indicate that the lower efficiency of illegitimate recombination between short repeats is buffered by their higher frequency in the genome. Some of the large deletions might also correspond to several consecutive shorter ones.

From this analysis we propose that accumulation of small and easily reversible indels is common in *S. agalactiae*, mostly occurring as a consequence of RecA independent recombination. Most of these indels are probably removed from the population due to purifying selection during long-term evolution. Alternatively they may be compensated by a second loss or gain of nucleotides. In the ST260-261 lineages host specialization led to the relaxation of the negative selection on genes that became dispensable, allowing the accumulation of larger deletions.

### Functional adaptation to a host restricted way of living

**Figure 6:**
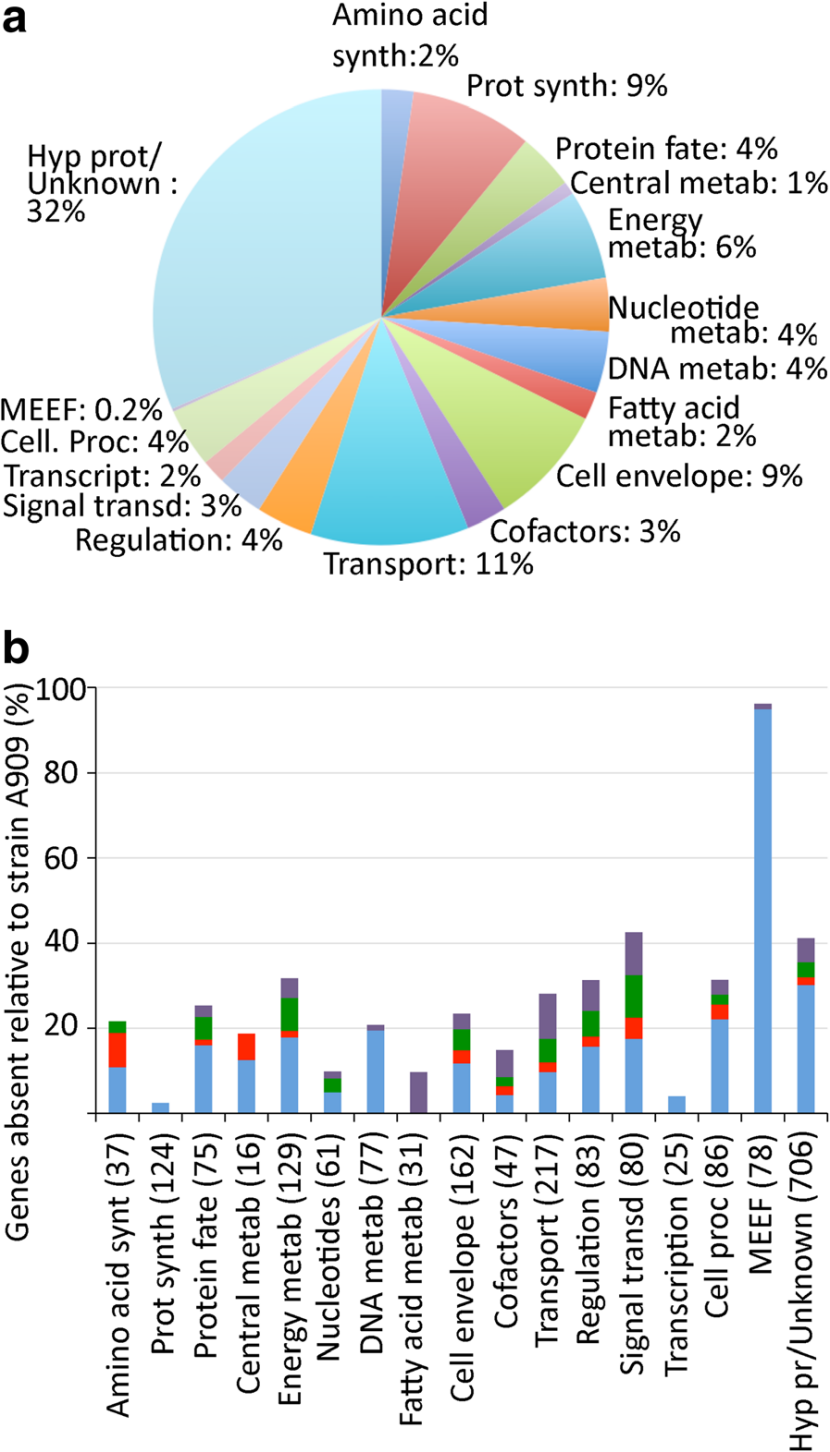
**Functional classification of the genes conserved or inactivated during the evolution of the ST260-ST261 sublineages.** (**a**) Functional classification of the genes of the ST260-261 strain core genome (KEGG classification). Only 1432 functional genes common to the ST260 and 261 sublineages were taken into account. MEEF stands for mobile and extrachromosomal element functions (**b**) Analysis of the genes present in strain A909 and inactivated (disrupted or deleted) in the ST260 and ST261 sublineages according to KEGG functional classification. In blue: Genes inactivated before the separation of the ST260-261 sublineages. In red: Genes inactivated by deletion in one sublineage and gene disruption in the other or by independent events in the two sublineages; in green and violet: genes inactivated in the ST261 or in the ST260 sublineage respectively. The number of genes in each functional category is indicated in brackets.

ST260-261 strains differ from other GBS strains by their host specificity and high pathogenicity. As mentioned, this lineage has only few specific genes compared to other strains and most of them are carried by genomic islands in the process of being eliminated. In particular, we did not identify any specific surface proteins or putative virulence factors. Therefore, the specific properties of the ST260-261 strains more likely rely on functions shared with human isolates and on the loss of some functions. The 1432 genes common to the two sublineages were grouped into functional categories according to KEGG classification (Figure 6a). Compared to functional classification of A909 genes, this analysis revealed a dramatic drop in the proportions of genes involved in mobile and extrachromosomal functions (that reflects the loss of most genomic islands) and in cellular processes, including the pathogenesis subcategory (Figure 6b). The other major functions submitted to evolutionary erosion were energy metabolism, transport and binding, regulation and signal transduction (Figure 6b). Conversely the basal functions of the cell such as transcription and protein synthesis were nearly not affected.

#### Bacterial-host interactions and virulence factors

Surface components play an important role for tissue colonization and infection by mediating interactions between the pathogen and the host cells and evasion from immune defense. GBS strains possess two distinct polysaccharide antigens, the highly sialylated capsule polysaccharide and the group B-antigen. ST260-261 strains harbor the 16 genes involved in the type Ib capsule synthesis (GBS222_0990-1005) similarly to the ST6 strains H36B and SS1014. They also possess the 16 genes responsible for the synthesis of the group B antigen (GBS222_1160-1175). Only seven proteins with a LPXTG signal for cell-wall anchor are conserved (Table 2) including three important *S. agalactiae* virulence factors of human strains: the Fibrinogen-binding protein A, the Serine-rich protein Srr1 and the BibA/HvgA protein [50-53]. However, while in human strains these three proteins carry a variable number of a repeated motif, these repeats have been lost in the ST260-261 strains. This might decrease their accessibility to host immune system and cellular receptors by tethering them to the cell wall.

**Table 2:** Potential virulence factors in the ST260-261 strains

**2-22**	**SS1219**	**90503**	**Gene**	**A909**	**Functional annotation**	**LPXTG**^**1**^	**Ref**	**Mechanism of pathogenicity**
pseudo^2^	GBS1219_1647	GBS90503_1646	*cfa/cfb*	SAK_1983	CAMP factor	no	[54]	***Pore-forming Toxins***
GBS222_0988-1005	GBS1219_979-0996	GBS90503_0979-0996	*cps*	SAK_1246-1263	Capsule synthesis enzymes	no	[55]	***Immune evasion***
GBS222_0661	GBS1219_0659	GBS90503_0659	*sod*	SAK_0913	Superoxide dismutase	no	[56]	
GBS222_0308	GBS1219_0307	GBS90503_0307	*pbp1*	SAK_0222	Penicillin-binding protein 1	no	[57]	***Resistance to host antimicrobial peptides***
GBS222_1019	GBS1219_1007	GBS90503_1007	*pavA*	SAK_1277	fibronectin/fibrinogen binding protein	no	[58]	***Adherence and invasion***
GBS222_0877^3^	GBS1219_0872^3^	GBS90503_08728^3^	*fbsA*	SAK_1142	Fibrinogen-binding protein A	yes	[51,52]	
GBS222_1210^3^	GBS1219_1192^3^	GBS90503_1192^3^	*srr*	SAK_1493	Serin-rich repeat protein	yes	[59]	
GBS222_0182	GBS1219_0182	GBS90503_0182	*sip*	SAK_0065	Surface immunogenic protein	no	[60]	
GBS222_1669^3^	GBS1219_1663^3^	GBS90503_1662^3^		SAK_2002	Surface protein, BibA family		[61]	
GBS222_1026	GBS1219_1014	GBS90503_1014	*hylB*	SAK_1284	Hyaluronidase	no	[60,62]	
GBS222_0382	GBS1219_0382	GBS90503_0382		SAK_0502	surface protein	yes		***Cell-wall anchored proteins of unknown functions***
GBS222_0644	GBS1219_0646	GBS90503_0646		SAK_0896	surface protein	yes		
GBS222_1220	GBS1219_1202	GBS90503_1202		SAK_1503	serine-rich surface protein	yes		
GBS222_1221	GBS1219_1203	GBS90503_1203		SAK_1504	surface protein, Amidase family	yes		

^1^: proteins anchored to the peptidoglycan by an LPXTG C-terminal motif.

^2^ : the gene has been inactivated.

^3^ : amino-acid repeats have been lost in the corresponding protein.

Among the other proteins that are virulence factors in human GBS isolates, the five strains lack the C5A peptidase, the laminin binding protein and components of the pilus. The *cyl* locus has also been inactivated in the two sublineages (Additional file 4: Figure S2) in agreement with the absence of detectable hemolytic activity on blood agar plates. Another major test for *S. agalactiae* identification is the detection of CAMP activity [63,64]. While both ST260 and 261 strains were reported to be negative for the CAMP factor reaction [20] we found that the gene encoding the CAMP factor was disrupted only in ST261 strains. We experimentally confirmed that strain SS1219 was negative for the CAMP test detection, probably because of a lower level of gene expression (see below).

Finally, the five ST260-261 strains express a fibrinogen/fibronectin binding protein of the PavA family and a hyaluronidase that were proposed to be virulence factors in GBS or other *Streptococcus* species [62,65,66] and could also have a role in fish infection.

Altogether, approximately 60% of the genes for proteins involved in pathogenesis and considered as important virulence genes in human stains [67] were affected by genome reduction. In this context the capsule could be a major virulence factor in the fish host, as also observed for *Streptococcus iniae*, another streptococcus species pathogenic for fish [68,69].

#### Metabolism

Analysis of the missing functions revealed a profound remodeling of the metabolism of strains of this lineage. Numerous transport systems for carbon sources (ABC transporters and phosphoenolpyruvate/carbohydrate phospho-transferase systems) and enzymes for degradation of polysaccharides (amylase, extracellular pullulanase, enzyme for degradation of arbutin) were missing or inactivated, reflecting a reduced capacity to utilize diverse carbon sources. In addition ST260-261 strains seem unable to utilize glycerol and glycerol phosphate as genes encoding the glycerol kinase, the glycerol dehydrogenase and the glycerol-phosphate permease are missing or mutated. Fermentative pathways utilizing pyruvate/acetate conversion are also altered, as the phosphotransacetylase gene is missing. In mammals, GBS is primarily considered as a commensal of the digestive tract, an environment rich in diverse C-sources. In contrast, reduction in the catabolic capacities observed in ST260-261 strains is in favor of a transition to an obligate pathogen style-of-life.

#### Transport functions

Approximately 20% of the genes associated with transport systems were missing or inactivated. The targeted functions were mostly the import of nutrients, in particular of C-sources and the transport of inorganic and metal ions. The Na+/H + antiporter, the K + uptake permease were inactivated in both sublineages. Therefore ST260-261 strains may be affected in ionic exchange and would have a reduced capacity to maintain their homeostasis in the face of a changing external environment.

#### Transcriptional networks

Globally, gene disruption and deletion events affected 19 out of the 93 transcriptional regulators predicted in human strains (20%). As much as 13 out of the 21 two-component systems (TCS) (60%) found in GBS were inactivated, either in one (six TCS) or in both (seven TCS) sublineages (Additional file 6: Table S6). Interestingly, analysis of the genome sequence of strain 2-22 revealed that the Rgf TCS, which is involved in the control of virulence in the human ST17 strains [70], was associated with two putative bacteriocins with a double glycine leader peptide and with a bacteriocin export transporter (Additional file 4: Figure S3). This suggests that this TCS originates from a bacteriocin operon that has been partially deleted in human strains. Altogether our observations show that *S. agalactiae* strains adapted to fish may have a reduced capacity to respond to environmental changes compared to human strains and only eight TCS, including the two major systems, CiaRH and CovRS, may be sufficient to allow GBS adaptation to the different environments encountered in fish. Interestingly, both CiaRH and CovRS were also involved in the regulation of virulence genes in GBS human strains [71,72]. The higher virulence of ST260-261 strains might also be due to the deregulated expression of some virulence genes, as observed in the transition from local to systemic infections in Group A Streptococci [73,74].

### Adaptation to fish is associated with broad changes in gene expression

**Figure 7:**
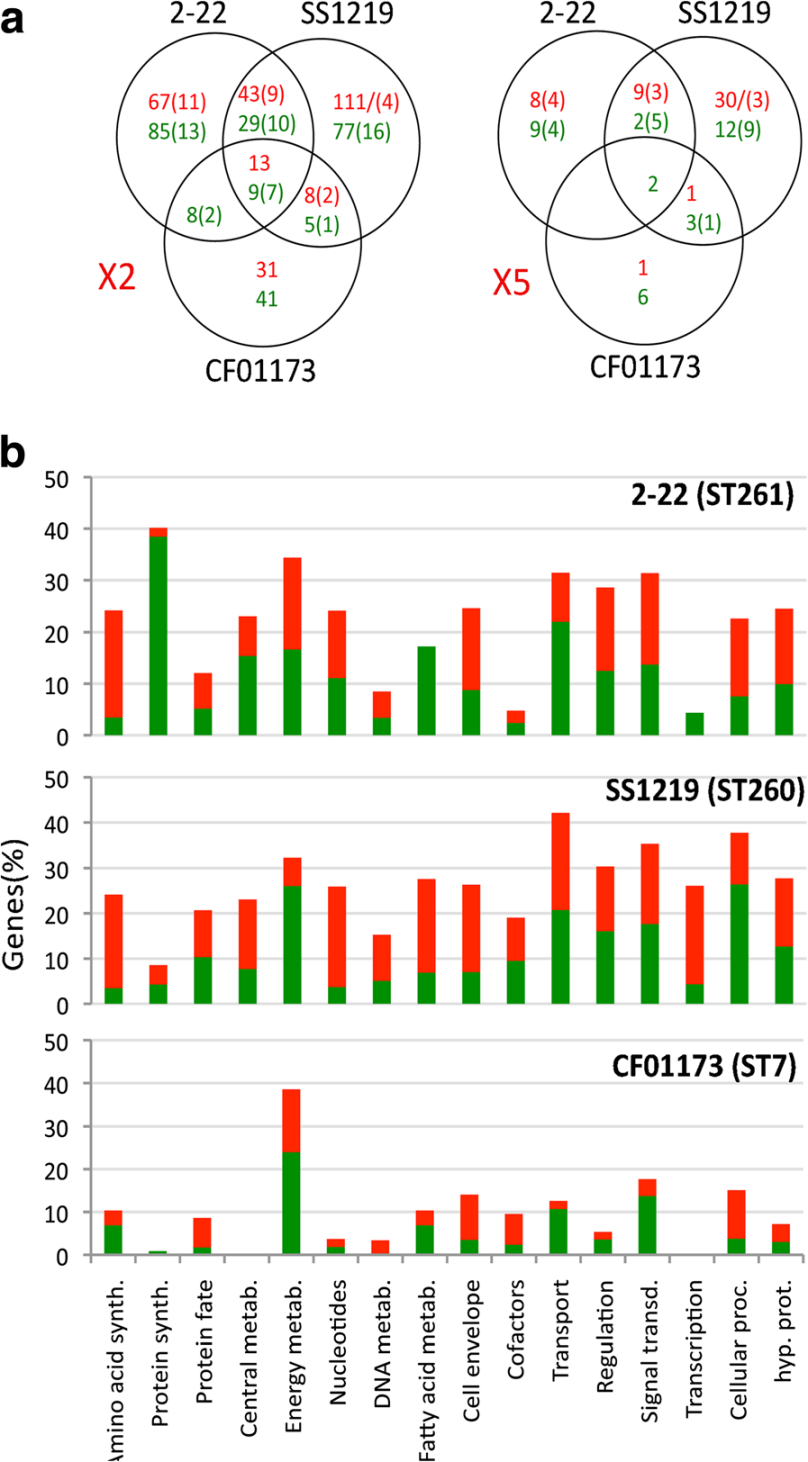
**Genes differentially expressed between GBS isolates from fish and the human A909 strain.** (**a**) Venn diagram showing the number of genes differentially expressed between the fish or frog isolates 2-22, SS1219 and CF01173 and strain A909; left panel: >2 fold variation; right panel: >5 fold variation. Numbers in green and red correspond to genes that are down- and up-regulated, respectively, in the fish isolates compared to strain A909. In brackets are the numbers of down- and up-regulated pseudogenes. (**b**) Proportions of up- (red bars) and down- (green bars) regulated genes, grouped by KEGG functional categories for the three fish or frog isolates relative to strain A909. Only the 1389 genes present in the four strains and with 100% identity matches with probes of the array were taken into account.

As a first step to explore changes in gene expression linked to host adaptation, we performed a comparative analysis at the transcriptome level of strains A909 (ST7 human isolate), CF01173 (ST7 fish isolate), 2-22 (ST261 fish isolate) and SS1219 (ST260 frog isolate). 1389 genes present in the four strains and with 100% identity matches with probes of the array were taken into account in this analysis (Additional file 7: Table S7). Profound modifications in gene expression were observed in ST260-261 strains and to a lesser extent in strain CF01173 compared to strain A909 (Figure 7a). Although strains CF01173 and A909 are closely related, the expression of more than 130 genes varied by a two-fold factor between the two strains. In particular 40% of the genes involved in energy metabolism were expressed at a lower level in the fish isolate than in strain A909. In contrast expression of the gene encoding the virulence protein Srr1 was more than 20 fold increased compared to strain A909. Although up-regulation of the *srr1* gene is not conserved in the ST260-261 fish isolates, in strain CF01173, it might facilitate penetration of the fish blood brain barrier, leading to an increased tendency to cause meningitis, as shown for the Srr1 protein of human isolates [59].

Both the number of genes for which expression was modified and the amplitude of these modifications were even larger in ST260-261 isolates (Figure 7a; Additional file 7: Table S7). For instance two genes encoding putative glyoxalases are expressed at 6-45 higher levels in ST260-261 strains than in strain A909, suggesting higher needs in methylglyoxal detoxification. Genes encoding the enzymes for the synthesis of arginine (arginosuccinate synthase and arginosuccinate lyase) were 10-15 fold more expressed. Four functional categories were more affected by variations in gene expression in both ST260-261 sublineages: metabolism, transport, regulation and signal transduction (Figure 7b). Interestingly these functional categories were also particularly affected by gene erosion, suggesting a remodeling of metabolic networks.

In addition to these global tendencies, ST260 and ST261 strains also harbor sublineage-specific variations in gene expression that probably result from differential gene decay and might lead to specific adaptations. For instance, in strain SS1219, genes encoding phosphoglycerate kinase, phosphoglyceromutase and pyruvate kinase involved in glycolysis and neoglucogenesis are expressed at a lower level than in strain A909. In parallel, genes coding for the arginine deiminase, ornithine carbamoylkinase and carbamate kinase involved in the deiminase pathway are up-regulated, suggesting a shift towards arginine fermentation as the main pathway for energy production in ST260 strains [75]. This up-regulation of the deiminase pathway is associated with a decreased expression of the negative regulator ArgR and the increased expression of the arginine/ornithine antiporter. In contrast, in the same growth conditions, upregulation of the genes for Hpr-kinase and CodY involved in catabolic repression and for lactate dehydrogenase suggests that strain 2-22 might generate energy essentially through glucose utilization and lactic fermentation. Iron import might be more efficient in ST260 than in ST261 strains since strain SS1219 expresses genes for iron ABC transporter and ferrichrome ABC transporter at higher levels than strain A909, while these genes are down-regulated or unchanged in strain 2-22. Both sublineages express lower amounts of ATP synthase, the enzyme responsible for ATP-proton motive force interconversion, than strain A909, indicating that they may be impaired in pH homeostasis in acidic conditions [76].

Since expression of pseudogenes is energetically costly and may generate deleterious products, we took advantage of the massive genome erosion in the ST260-261 lineage to look for evidence for negative selective pressure acting on pseudogene expression. Among the 184 pseudogenes represented by at least one probe on the array, only 22% were down-regulated compared to strain A909, whereas 64% did not present a significant change in mRNA levels and 14% were even up-regulated. Furthermore silencing of pseudogenes in the ST260-261 lineage did not seem to markedly increase with time. Indeed the proportion of down-regulated pseudogenes was similar among pseudogenes arising before (28%) and after the differentiation of sublineages (16% and 25%). Altogether this indicates that no strong evolutionary pressure acts to silence pseudogene expression.

Although modifications in gene expression generally affected different genes in ST260-261 and CC7 strains, 29 genes were found to vary in the same way in the 3 strains isolated from fish. Interestingly, these 29 genes included the operon for the capsule synthesis, the genes for zoocin and hyaluronidase, two response regulators of TCS systems (*ciaR* and *relR*) and three targets of the CiaRH TCS that were up-regulated. Sixteen genes were downregulated or inactivated in the three fish strains compared to A909, among which 11 were involved in energy metabolism or in carbohydrate uptake. Whether these regulations reflect a common mechanism of adaptation to fish environment remains to be established. However, the higher expression of the genes for the capsule synthesis might favor resistance to environmental conditions and to fish immune system, as it has also been reported for *S. iniae*[68]. Similarly the high hyaluronidase expression may help *S. agalactiae* strains to break through fish tissues and be involved in virulence to fish [62].

## Conclusions

Our results show that *S. agalactiae* strains leading to epidemic diseases in fish farms and cold blood animals belong to at least two distinct groups that differ by their strategies of host adaptation. CC7 strains have the potency to colonize and infect multiple hosts such as fish, human and cattle. From a genomic point of view, these CC7 fish strains are not distinguished from their human counterparts by any significant genomic island. However, contrasting with this genomic relatedness, large differences in gene expression were observed and could participate to the adaptation to the fish host. Conversely, our genome analysis indicates that strains of the ST260-261 complex diverged anciently from human and cattle strains and subsequently accumulated specific adaptations leading to the emergence of sublineages.

ST260-261 strains exhibit a striking pattern of genome reduction and we took advantage of the emergence of sublineages to reconstruct the different steps involved in this process. We found that accumulation of short indels can be observed all along the evolution of the GBS species, participating to strain-specific gene disruptions. Therefore even in the core genome of human GBS strains some genes are dispensable. Nevertheless the number of inactivated genes greatly increased during specialization of ST260-261 strains to fish. These gene inactivations mainly result from the ongoing accumulation of short indels, but a tendency to eliminate inactivated genes by deleting longer sequences is more noticeable in the sublineage specific branches. In contrast with what has previously been observed in the course of genomic reduction associated to host specialization or to intracellular symbiosis, deletion events are not correlated with an amplification of insertion sequences. Neither could complex genome rearrangements be noticed, suggesting that recombination between IS is not a general mechanism for genome reduction. Indeed our results point to non-homologous recombination as an alternative mechanism of genome reductive evolution.

## Methods

### Bacterial strains and growth conditions

*S. agalactiae* strains used in this study are described in Table 1. They were grown in Todd Hewitt medium (Difco) at 37°C except strains belonging to ST260-261 that were grown at 30°C.

### Genome sequencing and assembly methods

To perform the complete sequence of strain 2-22 (ATCC 51487), a mix of capillary Sanger and 454 pyrosequencing (Roche, http://www.roche.com) was carried out until ~24-fold coverage. A single and a 3kb insert size mate-paired libraries, leading to around 23-fold coverage of 454 GSflx reads (11-fold coverage and 12-fold coverage respectively), were added to Sanger reads, which was derived from a 10 kb insert fragment size library. This library was constructed after mechanical shearing of genomic DNA and cloning of generated inserts into plasmid pCNS (pSU18-derived). Plasmid DNAs were purified and end-sequenced (3633 reads) by dye-terminator chemistry with ABI3730 sequencers (Applied Biosystems, Foster City, USA) allowing an approximately 1-fold supplementary coverage of the genome. The reads were assembled by Newbler (Roche) and validated via the Consed interface (http://www.phrap.org). A first finishing step was performed using primer walking of clones and polymerase chain reactions (PCRs) (92 and 119 additional reads respectively). Around 70-fold coverage of Illumina reads (36 bp) were mapped, using SOAP (http://soap.genomics.org.cn), for the polishing phase as previously described [77]. Remaining gaps were then covered using PCRs with Platinum Hi-fidelity (InVitrogen) and primers specific to gap edges. The PCR fragments were sequenced by primer walking. Order and orientation of the sequences contiguous to ribosomal DNA was determined using long range PCR with PlatinumHI-fi (Invitrogen).

The genomes of the six other strains isolated from fish or frog were sequenced on the Illumina GAIIx with 36-cycle single-end chemistry and coverages of 100-300×. Reads were assembled using Velvet [78] to produce draft sequences. The number of contigs in each sequence is detailed in Additional file 8: Table S8. Contigs were reordered by Mauve 2.3.1 software [79] using the strain 2-22 genome as a reference for ST260-261 strains and the strain A909 genome for CC7 strains. When possible, overlapping ends of contigs were further assembled. Draft sequences were used to realign the reads using Bowtie [80], followed by alignment visualization with Tablet [81] to detect sequencing errors. Potential sequence ambiguities were only noticed for the ends of the contigs and therefore SNPs determined at ends of contigs were not considered as reliable.

### Nucleotide sequence accession number

The *S. agalactiae* 2-22 complete genome sequence is available from DDBJ/GenBank/EMBL under accession number FO393392. Draft genome sequences of strains 90-503, SS1219, CF01173, SS1014, SS1218 and 05-108A are available under accession numbers CAPZ01000001-CAPZ01000082, CAQA01000001-CAQA01000070, CAQB01000001-CAQB01000131, CAQC01000001-CAQC01000099, CAUB01000001-CAUB01000070 and CATH01000001-CATH01000087 respectively.

### Annotation methods

Annotation of strain 2-22 was performed using the CAAT-box environment as previously described [23]. Briefly, coding sequences (CDS) were defined by combining Genmark predictions with visual inspection of each open reading frame (ORF) for the presence of a start codon with an upstream ribosome-binding site and blastp similarity searches on Uniref 90, Trembl and Swissprot databases. Function predictions were based on blastp similarity searches and on the analysis of motifs using the PFAM databases [82]. For the six other strains putative CDS were determined using Artemis [83]. Predicted CDS were tested in reciprocal pair-wise blastp comparison against NEM316, A909 and 2-22 sequences. ORFs longer than 100 codons or encoding a protein > 90% identity by blastp with a protein annotated in NEM316, A909 or 2-22 were retained for further analyses. For genes with an orthologous sequence in other *S. agalactiae* genomes, assignment of the first codon and annotation were transferred from the orthologous genes. The remaining genes were manually annotated. Open reading frames whose lengths differed by more than 20% from orthologous sequences in NEM316 or A909 sequences were considered as putative pseudogenes and individually analyzed. Differences caused by variant start predictions were eliminated, as well as pseudogenes resulting from a mutation localized in the first or in the last 50 nucleotides of a contig, considered as more error-prone.

### Whole genome sequence comparisons

Determination of insertion/deletion events in ST260-261 strains compared to strain A909 [GenBank: NC_007432] was performed by combining blastn and nucmer analyses [40]. Each indel was individually inspected to differentiate possible misalignments from true indels and studied for the presence of repeat sequences at its extremities. We also aligned to A909 sequence the genome sequences of three *S. agalactiae* strains of human and bovine origins (NEM316, GenBank: [NC_004368]; 2603V/R [GenBank: AE009948] and FSL S3-O26/AEXT01000000 [GenBank: AEXT00000000] [24]). These alignments were used to produce tables of SNPs, indels and gaps relative to A909 genome sequence. Only indels that occurred internally to Velvet contigs in draft genome sequences and were repetitively observed for all strains of the same sequence type were taken into account. No significant difference in indel detection was noted using the whole-genome sequence of strain 2-22 or the Velvet-assembled contigs of the related ST261 strain SS1218. Indels relative to A909 core genome sequence were therefore classified into three categories related to strain phylogeny: i) detected in ST260-261 strains and in one or several other GBS strains, ii) observed for all strains belonging to the ST260-261 lineage but not detected in strains of bovine or human origins and iii) specific to one of the ST260-261 sublineages. For this last category, the allelic sequence identical to that of the human and bovine strains was considered to be the ancestral sequence, allowing to discriminate the mutated sequence and the nature of the mutation (insertion or deletion of nucleotides).

### Phylogenetic analysis

The genome sequence alignment was used to generate a table of SNPs from the *S. agalactiae* core genome. Regions that were recombined between strains A909 and SS1014 were removed from the analysis, as well as sequence gaps. In total 25,597 polymorphic positions in a 1.34 Mb core genome sequence were used to generate a consensus phylogenetic tree by the Maximum Likelihood method based on the Tamura-Nei model with the MEGA 5.04 software [41]. A bootstrap consensus tree was inferred from 1000 replicates. The same tree was obtained using a Neighbor-Joining method and computation of evolutionary distances by the p-distance method.

### SNP distribution

The SNP distribution between two genome sequences was inferred from genome alignment using nucmer and SNP were counted on a 1000 nt window. Indels were considered as unique mutation events and therefore counted as one SNP. The goodness of fit to a uniform distribution was tested using a Chi square test on class intervals of 50000 or 100000 nt as indicated in the figure legends.

### RNA preparation and transcriptome analyses

*S. agalactiae* strains were grown in TH medium at 30°C (strains 2-22 and SS1219) or 37°C (strain CF01173). For each transcriptome analysis, the reference strain A909 was grown at the same temperature as the tested strain. All bacterial cultures were harvested for RNA isolation at mid-exponential growth phase (OD 0.35-0.4). Total RNA was extracted as previously described [72]. RNA was prepared from three independent cultures and each RNA sample was hybridized twice to the microarrays (dye swap). RNA was reverse-transcribed with Superscript indirect cDNA kit (Invitrogen) and labeled with Cy5 or Cy3 (Amersham Biosciences) according to the supplier’s instructions. The microarray contains 12889 45-60mer oligonucleotides designed on the predicted gene sequences of strains NEM316, A909 and COH1. The oligonucleotide design was carried out with the OligoArray server [84] (http://berry.engin.umich.edu/oligoarray/). The microarray was manufactured by Agilent Technologies. Only probes with 100% identity to A909, CF00173, 2-22 and SS1219 sequences were taken into account in the data analysis (ie 4401 probes on 1389 genes). Analysis was performed as described [85]. The transcriptome data are MIAME compliant and have been submitted to the ArrayExpress database maintained at http://www.ebi.ac.uk/microarray-as/ae/ under the Accession Numbers E-MEXP-3828, E-MEXP-3830 and E-MEXP-3829.

## Abbreviations

GBS: Group B Streptococcus; IS: Insertion sequence; GI: Genomic island; MGE: Mobile genetic element; ICE: Integrative conjugative element; MLST: Multilocus sequence typing; CC: Clonal complex; ST: Sequence type; TCS: Two-component system; CAMP factor: Christie Atkins Munch-Petersen factor; SNP: Single nucleotide polymorphism; PTS: Phosphotransferase system; CDS: Coding sequence; ORF: Open reading frame.

## Competing interests

The authors declare that they have no competing interests.

## Authors’ contributions

IRC and PG conceived the study and wrote the article; IRC, PG and CR annotated the sequences; BM, SM, LM, CB and VB obtained the sequence data; EC prepared the biological material; EC, VDC and IRC performed and analyzed the microarray experiments; IRC analyzed the sequence data. All authors have read and approved the manuscript for publication.

## Supplementary Material

Additional file 1: Table S1 Shows the results of genome sequence comparison between the seven GBS fish/frog isolates and human and bovine isolates. **Table S2** describes the number of SNPs between the genome sequences of GBS strains isolated from human and fish. Click here for file

Additional file 2: Table S3 Lists the pseudogenes identified in strains CF01173 and SS1014. Click here for file

Additional file 3: Table S4 Provides a list of the insertion sequences identified in the genome sequence of the seven GBS strains under study. Click here for file

Additional file 4: Figure S1 Shows a comparison of genomic island 3.2 between strains 2-22, SS1219 and A909. **Figure S2** is a comparison of the organization of *pil*2, *cyl*, C5a peptidase and alpha-like protein loci in the ST260-261 strains versus strains isolated from human. **Figure S3** shows the organization of the *rgf* locus in ST260-261 and the sequences of the two putative bacteriocin-like peptides. Click here for file

Additional file 5: Table S5 Lists the pseudogenes identified in ST260-261 strains. Click here for file

Additional file 6: Table S6 Lists the two-component systems and transcription factors annotated in the genome sequences of ST260 and 261 strains. Click here for file

Additional file 7: Table S7 Shows the results of Microarray expression analysis for strains 2-22 (ST261), SS1219 (ST260) and CF01173 (ST7). Click here for file

Additional file 8: Table S8 Provides the characteristics of the Illumina reads and of contigs generated by Velvet. Click here for file
