# Supplementary material for: Reductive evolution in Streptococcus agalactiae and the emergence of a host adapted lineage
Source: BMC Genomics. 2013 Apr 15;14:252. doi: 10.1186/1471-2164-14-252 (PMC3637634; doi:10.1186/1471-2164-14-252)
Supplement: Additional file 1: Table S1 — Shows the results of genome sequence comparison between the seven GBS fish/frog isolates and human and bovine isolates. Table S2 describes the number of SNPs between the genome sequences of GBS strains isolated from human and fish. [file 1471-2164-14-252-S1.pdf]

Table S1: Genome sequence comparison of the seven GBS fish/frog strains against human and bovine GBS strains. The values correspond to the percentage of bases of the fish strain aligned to the human strains A909, NEM316 and 2603V/R or to the bovine strain FSLS3-026; numbers in brackets indicate the average identity of the sequence of the fish strain compared to the sequence of the human and bovine strains, as globally determined with nucmer [72].

Strain[1]	A909[2] (human ST7)	NEM316[3] (human ST23)	2603V/R[4] (human ST110)	FSLS3-026[5] (bovine ST67)
2-22 (ST261)	97.15 (98.95)	95.43 (98.89)	95.64 (98.92)	92.27 (98.91)
05-108A (ST260)	96.72 (98.92)	94.92 (98.9)	95.37 (98.88)	93.05 (98.88)
90-503 (ST260)	96.67 (98.93)	94.87 (98.92)	95.32 (98.89)	93.00 (98.89)
SS1219 (ST260)	96.75 (98.93)	94.97 (98.92)	95.41 (98.89)	93.14 (98.89)
SS1218 (ST261)	97.05 (98.95)	95.27 (98.95)	95.49 (98.93)	92.74 (98.92)
SS1014 (ST6)	95.80 (99.79)	91.62 (99.33)	91.32 (99.45)	85.88 (99.12)
CF01173 (ST7)	99.30 (99.97)	91.28 (99.35)	91.31 (99.48)	87.08 (99.03)

[1]: Sequence type is indicated in brackets
[2]: Accession number [GenBank: NC_007432]
[3]: Accession number [GenBank: NC_004368]
[4]: Accession number [GenBank: AE009948]
[5]: Accession number [GenBank: AEXT00000000]

Table S2: Number of SNP between the genome sequences of GBS strains isolated from human and fish/frog. Values were determined using nucmer [72].

Strain	ST, Serotype	2-22	SS1218	90-503	SS1219	05-108A	CF01173	SS1014
**A909**	**ST7, Ia**	15475	15524	15609	15616	15731	389	3484
**H36B**	**ST6,Ib**	15431	15487	15552	15552	15669	3625	689
**NEM316**	**ST23, III**	15275	15262	15356	15273	15376	10955	11158
**2603V/R**	**ST110,V**	15601	15601	15728	15633	15748	8659	9129
**2-22**	**ST261, Ib**		30	3073	3083	3098	15427	15356
**SS1218**	**ST261, Ib**	30		3067	3083	3086	15526	15446
**90-503**	**ST260,Ib**	3073	3067		132	49	15716	15602
**SS1219**	**ST260,Ib**	3083	3083	132		132	15627	15505
**05-108A**	**ST260,Ib**	3098	3086	49	132		15733	15620
**STIR-CD-17[1]**	**ST260,Ib**	3373	3308	243	335	219	15888	15776
**SA20-06[2]**	**ST552,Ib**	3376	3325	1685	1681	1715	15897	15791
**CF01173**	**ST7, Ia**	15427	15526	15716	15627	15733		3514
**ZQ0910[3]**	**ST7,Ia**	15464	15465	15637	15593	15647	105	3109
**GD201008-001[4]**	**ST7,Ia**	15520	15466	15639	15603	15665	100	3090
**SS1014**	**ST6,Ib**	15356	15446	15602	15505	15620	3514	

[1]: Accession number [GenBank: ALXB01000000]
[2]: Accession number [GenBank: CP003919]
[3]: Accession number [GenBank: NZ_AKAP01000000]
[4]: Accession number [GenBank: CP003810]
