# Supplementary material for: Reductive evolution in Streptococcus agalactiae and the emergence of a host adapted lineage
Source: BMC Genomics. 2013 Apr 15;14:252. doi: 10.1186/1471-2164-14-252 (PMC3637634; doi:10.1186/1471-2164-14-252)
Supplement: Additional file 2: Table S3 — Lists the pseudogenes identified in strains CF01173 and SS1014. [file 1471-2164-14-252-S2.pdf]

Table S3 : Pseudogenes in strains CF01173 and SS1014

gene name	ortholog in A909	Functional Annotation
**Pseudogenes in strain CF01173**		
Pseudogenes specific to strain CF01173		
GBS1173_0076	SAK_0137	
GBS1173_0154	SAK_0227	ComG operon protein 2, putative
GBS1173_0487	SAK_0661	Cof-like hydrolase
GBS1173_0582	SAK_0766	serine/threonine protein kinase, putative
GBS1173_0595	SAK_0778	sortase family protein
GBS1173_0624	SAK_0700	ABC transporter, permease protein Vexp3
GBS1173_0716	SAK_0906	ComE operon protein 1, putative
GBS1173_0802	SAK_0997	exonuclease RexA
GBS1173_0871	SAK_1068	nisin resistance protein Nsr, putative
GBS1173_0874	SAK_1072	sensor histidine kinase
GBS1173_0907	SAK_1104	iron chelate uptake ABC transporter, permease protein
GBS1173_1530	SAK_1768	acetyltransferase
GBS1173_1636	SAK_1880	sensor histidine kinase, putative
GBS1173_1673	SAK_1918	response regulator
Pseudogenes common to strains A909 and CF01173		
GBS1173_0385	SAK_0497	C5a peptidase ScpA
GBS1173_0395	SAK_0512	alcohol dehydrogenase
GBS1173_0418	SAK_0536	galactokinase
GBS1173_0518		prophage LambdaSa2, site-specific recombinase, phage integrase
GBS1173_0618	SAK_0806	endopeptidase O
GBS1173_1110	SAK_1329	FtsK/SpoIIIE family protein
GBS1173_1356	SAK_1586	phosphoserine aminotransferase
GBS1173_1476	SAK_1714	
GBS1173_1524	SAK_1763	Transcriptional regulator

GBS1173_1525	SAK_1764	short-chain dehydrogenase
GBS1173_1618	SAK_1861	major facilitator family protein
GBS1173_1671	SAK_1916	ABC transporter, ATP-binding protein

Pseudogenes in strain SS1014

Pseudogenes specific to strain SS1014

GBS1014_0134	SAK_0188	sensor histidine kinase
GBS1014_0458	SAK_0557	Ser/Thr protein phosphatase family protein
GBS1014_0774	SAK_0927	major facilitator family protein
GBS1014_0931	SAK_1088	NOL1/NOP2/sun family putative RNA methylase
GBS1014_1350	SAK_1520	transcriptional activator, Rgg/GadR/MutR family
GBS1014_1765		hypothetical protein
GBS1014_1934	SAK_2120	B3/4 domain protein

Pseudogenes common to A909 and SS1014

GBS1014_0406	SAK_0497	C5a peptidase ScpA
GBS1014_0419	SAK_0512	oxidoreductase, zinc-binding dehydrogenase family
GBS1014_0543	SAK_0694	site-specific recombinase, phage integrase family
GBS1014_0545	SAK_0696	ISSag6, transposase orfB
GBS1014_0658	SAK_0811	transport protein, putative
GBS1014_1058	SAK_1219	conserved hypothetical protein
GBS1014_1163	SAK_1329	conserved hypothetical protein
GBS1014_1540	SAK_1714	aminopeptidase P, authentic point mutation
GBS1014_1592	SAK_1764	oxidoreductase, short chain dehydrogenase/reductase family
GBS1014_1766	SAK_1951	conserved domain protein
