# Supplementary material for: Reductive evolution in Streptococcus agalactiae and the emergence of a host adapted lineage
Source: BMC Genomics. 2013 Apr 15;14:252. doi: 10.1186/1471-2164-14-252 (PMC3637634; doi:10.1186/1471-2164-14-252)
Supplement: Additional file 3: Table S4 — Provides a list of the insertion sequences identified in the genome sequence of the seven GBS strains under study. [file 1471-2164-14-252-S3.pdf]

Table S4: **Insertion sequences identified in the genome sequence of the fish GBS strains.** The number of insertion sequences for strains 05-108A, 90-503, SS1219, SS1218, CF01173 and SS1014 was estimated based on the ratio between the sequence coverage for the IS and the medium coverage for other non repetitive genomic sequences.

	ISAG1	ISAG8	GBSi1	IS861	IS1381	ISSag9	ISSAg4 (ISAG2)	ISSag5
2-22	11							
05-108A	13-21							
90-503	15-20							
SS1219	14-16							
SS1218	10-11							
CF01173	1	9	7	2-3	6-8	4	2	2-3
A909	1	5		2	6	3	2	
SS1014	0				10-11	1	2	2
H36B	1				8	1	2	
