# Supplementary material for: Reductive evolution in Streptococcus agalactiae and the emergence of a host adapted lineage
Source: BMC Genomics. 2013 Apr 15;14:252. doi: 10.1186/1471-2164-14-252 (PMC3637634; doi:10.1186/1471-2164-14-252)

Figure S1: Comparison of the genomic Island 3.2 in human and fish isolates. Alignment of region 3.2 of strain A909 on genomic sequence of strains 2-22 (ST261) and SS1219 (ST260). Regions with > 70 % nucleotide identity are indicated by blue bars. Genes coding for the two PTS for galactitol, for sugar transporter and for galactose utilization are shown. Pseudogenes are marked by red stars.

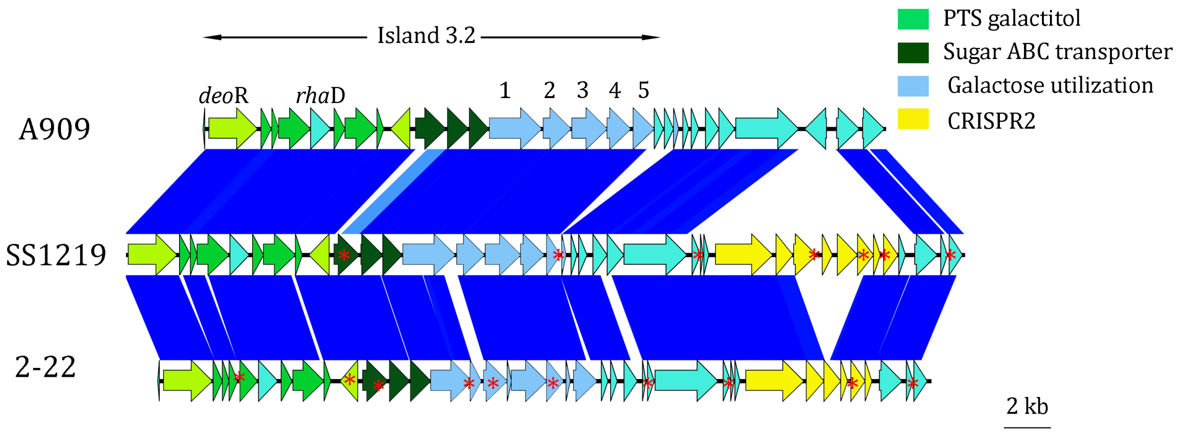

Figure S2: Comparison of the organization of the *pil*2 (a), *cyl* (b), C5a peptidase (c) and alpha-like protein (d) loci in ST260-261 strains versus strains isolated from human.

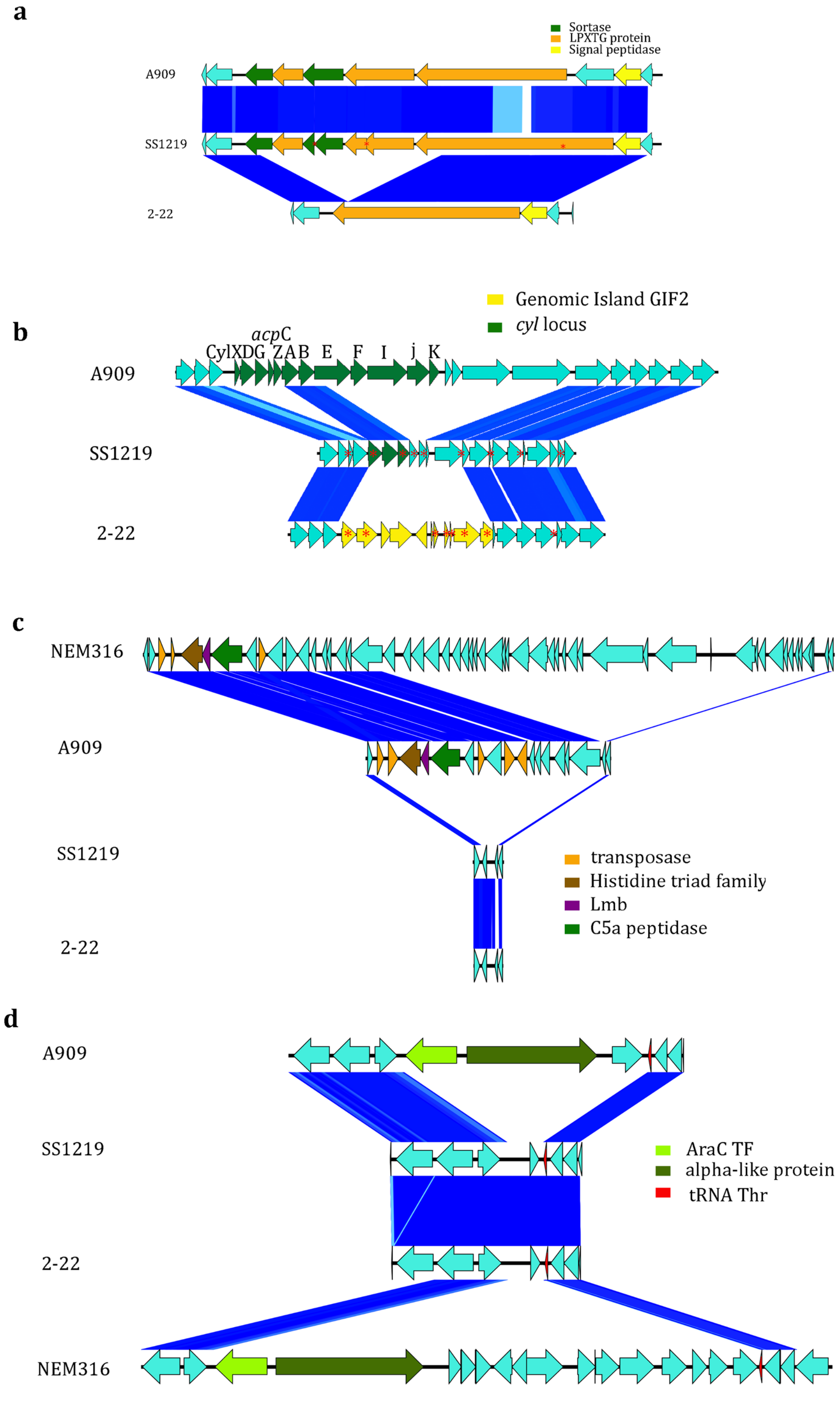

Figure S3: Organization of the *rgf* locus in ST260-261 strains. (a): sequence comparison of the *rgf* locus in various streptococci; (b,c) : sequence comparison of the two bacteriocin-like peptides found in strain 2-22. (d) comparison of the locus in four *S. agalactiae* strains. *GBS222_1615* encodes a putative bacteriocin_II precursor with a double-glycine leader peptide leading to a 47AA-long peptide after cleavage. This peptide, similar to sanguinicin K11 [GenBank: ABX39517] and to a putative bacteriocin from *S. equi* lacks the YGNGVXC-motif characteristic of class II.1 pediocin-like bacteriocins. *GBS222_1615* is associated with genes for a sensor histidine kinase and a response regulator (*GBS222_1618-1619*), as well as for a BlpC-like peptide pheromone with a double-glycine leader peptide (*GBS222_1617*) and a BlpA-like bacteriocin export ABC transporter (*GBS222_1616*). Unlike in *S. thermophilus*, this locus lacks the gene for the accessory transporter protein that is however variably found in streptococcal Blp loci.

Red arrows: genes encoding bacteriocin-like peptides, brown arrows: bacteriocin export ABC transporter, dark blue arrows: accessory transporter protein, light green: response regulator, dark green: histidine kinase.

a

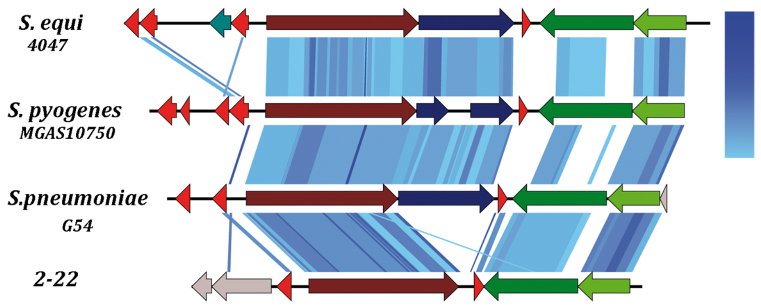

b

```
GBS222_1615 MNVKSIEQLEVMDNEALSHVEGGIGHRWICSKNGKHTYTSAWHLSEKTAAANARNHMALYPGSVCRVYHV
S. equi     MNTKTVEPFSTMTADMLAGVEGGWGYRWRCT----DGYTSAWHLLRDTAQENADNHMILYPGTVCRVYN
Sanguinicin MDTKVMSQFDVLDENTLATVEGGWGYVWQCS----NKYKSAWHAQRRTAQENANAYMRIHRGVVCAVFN
SAI_2022    MKHYHMLKVALVIDGYTVRT-------------GKHTYTSAGHLSEKTAAANARNRMGLYPGSVCRVYHV
```

c

```
GBS222_1617  MKQNTTLNFKTNFQELSPEQLNNITGGGWIDDIKKIINLDKLNFLRL
S. equi      MDKPTRQ    PKFQELTPSQLTQITGGGWLEGLQKVFNVSKPKLLK
S.mitis BlpC MDKNQNL    TSFQELTTTELNQITGGGWWEDILYTLNIIKYNNTKGLHHPIQ
S. pneumo    MDKKQNL    ASFQELTTTELNQITGGGLWEDILYSLNIIKHNNTKGLHHPIQL
SAN_2057     MELQL         QELSPEQLNNITGGGWIDGIKKIINLDKLNFLKL
```

d

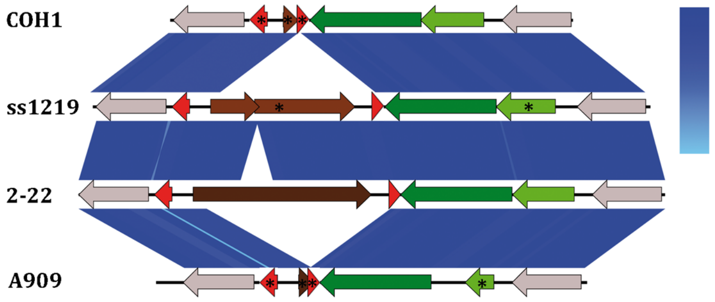

Supplement: Additional file 4: Figure S1 — Shows a comparison of genomic island 3.2 between strains 2-22, SS1219 and A909. Figure S2 is a comparison of the organization of pil2, cyl, C5a peptidase and alpha-like protein loci in the ST260-261 strains versus strains isolated from human. Figure S3 shows the organization of the rgf locus in ST260-261 and the sequences of the two putative bacteriocin-like peptides. [file 1471-2164-14-252-S4.pdf]
