# Supplementary material for: Reductive evolution in Streptococcus agalactiae and the emergence of a host adapted lineage
Source: BMC Genomics. 2013 Apr 15;14:252. doi: 10.1186/1471-2164-14-252 (PMC3637634; doi:10.1186/1471-2164-14-252)
Supplement: Additional file 5: Table S5 — Lists the pseudogenes identified in ST260-261 strains. [file 1471-2164-14-252-S5.pdf]

Table S5: Pseudogenes identified in ST260 and ST261 strains

A. Pseudogenes shared by the five ST260/261 GBS strains

SS1219 locus_tags		90-503 locus_tags		05-108A locus_tags		2-22 locus_tag		ortholog in A909	Functional annotation	KEGG category
GBS1219_0008	pseudo	GBS90503_0008	pseudo	GBS05108_0008	pseudo	GBS222_0005	pseudo	SAK_0005	hypothetical protein	hypothetical protein
GBS1219_0023	pseudo	GBS90503_0023	pseudo	GBS05108_0023	pseudo	GBS222_0017	pseudo	SAK_0328	acetyltransferase, GNAT family	Unknown function
GBS1219_0038	pseudo	GBS90503_0038	pseudo	GBS05108_0038	pseudo	GBS222_0034	pseudo	SAK_0345	glycerol-3-phosphate oxidase	Energy metabolism
GBS1219_0092	pseudo	GBS90503_0092	pseudo	GBS05108_0092	pseudo	GBS222_0090	pseudo	SAK_0400	PTS system, IIC component, lactose/cellobiose family	Signal transduction
GBS1219_0093	pseudo	GBS90503_0093	pseudo	GBS05108_0093	pseudo	GBS222_0091	pseudo	SAK_0404	cysteine synthase/cystathionine beta-synthase family protein	Amino acid biosynthesis
GBS1219_0115	pseudo	GBS90503_0115	pseudo	GBS05108_0115	pseudo	GBS222_0113	pseudo	SAK_0431	membrane protein, putative	Cell envelope
GBS1219_0130	pseudo	GBS90503_0130	pseudo	GBS05108_0130	pseudo	GBS222_0127	pseudo	SAK_0445	hypothetical protein	
GBS1219_0185	pseudo	GBS90503_0185	pseudo	GBS05108_0185	pseudo	GBS222_0185	pseudo	SAK_0068	carbohydrate uptake 1 (CUT1) family ABC transporter, permease protein	Transport and binding proteins
GBS1219_0191	pseudo	GBS90503_0191	pseudo	GBS05108_0191	pseudo	GBS222_0191	pseudo	SAK_0074	similar to acetyl xylan esterase (hypothetical)	Energy metabolism
GBS1219_0240	pseudo	GBS90503_0240	pseudo	GBS05108_0240	pseudo	GBS222_0241	pseudo	SAK_0140	Conserved hypothetical protein	
GBS1219_0272	pseudo	GBS90503_0272	pseudo	GBS05108_0272	pseudo	GBS222_0273	pseudo	SAK_0174	Similar to similar to two-component response regulator	Signal transduction
GBS1219_0319	pseudo	GBS90503_0319	pseudo	GBS05108_0319	pseudo	GBS222_0319	pseudo	SAK_0237	conserved hypothetical protein	conserved hypothetical protein
GBS1219_0320	pseudo	GBS90503_0320	pseudo	GBS05108_0320	pseudo	GBS222_0320	pseudo	SAK_0238	CAAX amino terminal protease family protein	Unknown function
GBS1219_0340	pseudo	GBS90503_0340	pseudo	GBS05108_0340	pseudo	GBS222_0340	pseudo	SAK_0258	alpha amylase family protein	Energy metabolism
GBS1219_0346	pseudo	GBS90503_0346	pseudo	GBS05108_0346	pseudo	GBS222_0346	pseudo	SAK_0264	oxidoreductase, NAD-binding	Unknown function
GBS1219_0387	pseudo	GBS90503_0387	pseudo	GBS05108_0387	pseudo	GBS222_0387	pseudo	SAK_0510	cupin domain protein	Unknown function
GBS1219_0393	pseudo	GBS90503_0393	pseudo	GBS05108_0393	pseudo	GBS222_0393	pseudo		conserved hypothetical protein	
GBS1219_0406	pseudo	GBS90503_0406	pseudo	GBS05108_0406	pseudo	GBS222_0406	pseudo	SAK_0532	Sugar ABC transporter, sugar-binding protein	Transport and binding proteins
GBS1219_0419	pseudo	GBS90503_0419	pseudo	GBS05108_0419	pseudo	GBS222_0419	pseudo		putative Fic protein family protein	
GBS1219_0422	pseudo	GBS90503_0422	pseudo	GBS05108_0422	pseudo	GBS222_0422	pseudo		CRISPR-associated protein Cas4	
GBS1219_0425	pseudo	GBS90503_0425	pseudo	GBS05108_0425	pseudo	GBS222_0423	pseudo		CRISPR-associated protein Cas1	
GBS1219_0428	pseudo	GBS90503_0428	pseudo	GBS05108_0428	pseudo	GBS222_0426	pseudo	SAK_0550	CorA family metal ion transporter	Transport and binding proteins
GBS1219_0435	pseudo	GBS90503_0435	pseudo	GBS05108_0435	pseudo	GBS222_0433	pseudo	SAK_0556	similar to unknown proteins	
GBS1219_0436	pseudo	GBS90503_0436	pseudo	GBS05108_0436	pseudo	GBS222_0434	pseudo	SAK_0557	Ser/Thr protein phosphatase family protein	Unknown function
GBS1219_0451	pseudo	GBS90503_0451	pseudo	GBS05108_0451	pseudo	GBS222_0449	pseudo	SAK_0573	similar to glucose kinase	Energy metabolism
GBS1219_0467	pseudo	GBS90503_0467	pseudo	GBS05108_0467	pseudo	GBS222_0465	pseudo	SAK_0589	Similar to unknown proteins	Unknown function
GBS1219_0534	pseudo	GBS90503_0534	pseudo	GBS05108_0534	pseudo	GBS222_0532	pseudo		Putative DNA methylase	
GBS1219_0535	pseudo	GBS90503_0535	pseudo	GBS05108_0535	pseudo	GBS222_0533	pseudo		conserved hypothetical protein	
GBS1219_0541	pseudo	GBS90503_0541	pseudo	GBS05108_0541	pseudo	GBS222_0535	pseudo		putative integrase	
GBS1219_0554	pseudo	GBS90503_0554	pseudo	GBS05108_0554	pseudo	GBS222_0553	pseudo		putative ATPases of the AAA+ class	
GBS1219_0573	pseudo	GBS90503_0573	pseudo	GBS05108_0573	pseudo	GBS222_0574	pseudo	SAK_0826	2-dehydro-3-deoxyphosphogluconate	Amino acid biosynthesis
GBS1219_0578	pseudo	GBS90503_0578	pseudo	GBS05108_0578	pseudo	GBS222_0579	pseudo	SAK_0831	putative Glycosyl hydrolase, family 3	Energy metabolism
GBS1219_0581	pseudo	GBS90503_0581	pseudo	GBS05108_0581	pseudo	GBS222_0582	pseudo	SAK_0834	similar to alpha-amylase	Energy metabolism
GBS1219_0631	pseudo	GBS90503_0631	pseudo	GBS05108_0631	pseudo	GBS222_0632	pseudo	SAK_0884	Putative oligopeptidase	Protein fate
GBS1219_0654	pseudo	GBS90503_0654	pseudo	GBS05108_0654	pseudo	GBS222_0656	pseudo	SAK_0908	putative Hydrolase, haloacid dehalogenase-like family	Unknown function
GBS1219_0666	pseudo	GBS90503_0666	pseudo	GBS05108_0666	pseudo	GBS222_0668	pseudo	SAK_0920	Hypothetical protein	
GBS1219_0667	pseudo	GBS90503_0667	pseudo	GBS05108_0667	pseudo	GBS222_0669	pseudo	SAK_0921	MarR family transcriptional regulato	Regulatory functions
GBS1219_0687	pseudo	GBS90503_0687	pseudo	GBS05108_0687	pseudo	GBS222_0688	pseudo	SAK_0940	conserved hypothetical prtotein	Transport and binding proteins
GBS1219_0702	pseudo	GBS90503_0702	pseudo	GBS05108_0702	pseudo	GBS222_0703	pseudo	SAK_0955	Surface protein	Unknown function
GBS1219_0724	pseudo	GBS90503_0724	pseudo	GBS05108_0724	pseudo	GBS222_0726	pseudo	SAK_0978	Putative glucose-1-phosphate adenylyltransferase	Energy metabolism
GBS1219_0767	pseudo	GBS90503_0767	pseudo	GBS05108_0767	pseudo	GBS222_0769	pseudo		Hypothetical protein	
GBS1219_0795	pseudo	GBS90503_0795	pseudo	GBS05108_0795	pseudo	GBS222_0798	pseudo	SAK_1048	Bmp family membrane protein	Cell envelope
GBS1219_0796	pseudo	GBS90503_0796	pseudo	GBS05108_0796	pseudo	GBS222_0799	pseudo	SAK_1050	carbohydrate ABC transporter periplasmic-binding protein	Transport and binding proteins
GBS1219_0805	pseudo	GBS90503_0805	contig end	GBS05108_0805	pseudo	GBS222_0808	pseudo	SAK_1059	Similar to Na+/H+ antiporter	Transport and binding proteins
GBS1219_0811	pseudo	GBS90503_0811	pseudo	GBS05108_0811	pseudo	GBS222_0814	pseudo	SAK_1067	Similar to unknown protein	

GBS1219_0832	pseudo	GBS90503_0832	pseudo	GBS05108_0832	pseudo	GBS222_0836	pseudo	SAK_1088	NOL1/NOP2/sun family putative RNA methylase	Protein synthesis
GBS1219_0858	pseudo	GBS90503_0858	pseudo	GBS05108_0858	contig end	GBS222_0863	pseudo	SAK_1128	conserved hypothetical protein	conserved hypothetical protein
GBS1219_0888	pseudo	GBS90503_0887	pseudo	GBS05108_0887	pseudo	GBS222_0894	pseudo	SAK_1156	putative ABC transporter (ATP-binding protein)	Transport and binding proteins
GBS1219_0889	pseudo	GBS90503_0888	pseudo	GBS05108_0888	pseudo	GBS222_0895	pseudo	SAK_1157	putative ABC transporter	Transport and binding proteins
GBS1219_0907	pseudo	GBS90503_0906	pseudo	GBS05108_0906	pseudo	GBS222_0913	pseudo	SAK_1175	similar to cation (K+) transport protein	Transport and binding proteins
GBS1219_0909	pseudo	GBS90503_0908	pseudo	GBS05108_0908	pseudo	GBS222_0915	pseudo	SAK_1177	phosphotransacetylase	Energy metabolism
GBS1219_0920	pseudo	GBS90503_0919	pseudo	GBS05108_0919	pseudo	GBS222_0926	pseudo	SAK_1188	Similar to 6-phospho-beta-glucosidase	Energy metabolism
GBS1219_0922	pseudo	GBS90503_0921	pseudo	GBS05108_0921	pseudo	GBS222_0928	pseudo		putative (3R)-hydroxymyristoyl-[acyl-carrier-protein] dehydratase	
GBS1219_0926	pseudo	GBS90503_0926	pseudo	GBS05108_0926	pseudo	GBS222_0932	pseudo		Replication initiation factor	
GBS1219_0928	pseudo	GBS90503_0928	pseudo	GBS05108_0928	pseudo	GBS222_0934	pseudo		Integrase/recombinase, phage associated, putative	
GBS1219_0932	pseudo	GBS90503_0932	pseudo	GBS05108_0932	pseudo	GBS222_0938	pseudo	SAK_1192	chloride channel (ClC) family protein	Transport and binding proteins
GBS1219_0946	pseudo	GBS90503_0946	pseudo	GBS05108_0946	pseudo	GBS222_0952	pseudo	SAK_1206	Polysaccharide deacetylase family protein	Energy metabolism
GBS1219_0955	pseudo	GBS90503_0955	pseudo	GBS05108_0955	pseudo	GBS222_0961	pseudo	SAK_1218	Conserved hypothetical protein	
GBS1219_1031	pseudo	GBS90503_1031	pseudo	GBS05108_1031	pseudo	GBS222_1043	pseudo	SAK_1301	similar to putative permease	Cell envelope
GBS1219_1032	pseudo	GBS90503_1032	pseudo	GBS05108_1032	pseudo	GBS222_1044	pseudo	SAK_1302	putative pullulanase	Energy metabolism
GBS1219_1041	pseudo	GBS90503_1041	pseudo	GBS05108_1041	pseudo	GBS222_1053	pseudo	SAK_1311	Conserved hypothetical protein	Unknown function
GBS1219_1063	pseudo	GBS90503_1063	pseudo	GBS05108_1063	pseudo	GBS222_1074	pseudo	SAK_1352	similar to unknown proteins	
GBS1219_1093	pseudo	GBS90503_1093	pseudo	GBS05108_1093	pseudo	GBS222_1108	pseudo	SAK_1389	Transcriptional regulator, RofA family	Regulatory functions
GBS1219_1131	pseudo	GBS90503_1131	pseudo	GBS05108_1131	pseudo	GBS222_1146	pseudo	SAK_1427	hypothetical protein	
GBS1219_1173	pseudo	GBS90503_1173	pseudo	GBS05108_1173	pseudo	GBS222_1184	pseudo	SAK_1470	hypothetical protein	
GBS1219_1183	pseudo	GBS90503_1183	pseudo	GBS05108_1183	pseudo	GBS222_1196	pseudo		hypothetical protein	
GBS1219_1193	pseudo	GBS90503_1193	pseudo	GBS05108_1193	pseudo	GBS222_1211	pseudo	SAK_1494	Similar to transcription regulator RofA related-truncated	
GBS1219_1195	pseudo	GBS90503_1195	pseudo	GBS05108_1195	pseudo	GBS222_1213	pseudo	SAK_1496	Similar to unknown proteins	Unknown function
GBS1219_1221	pseudo	GBS90503_1221	pseudo	GBS05108_1221	pseudo	GBS222_1239	pseudo	SAK_1522	hypothetical protein	
GBS1219_1271	pseudo	GBS90503_1271	pseudo	GBS05108_1271	pseudo	GBS222_1289	pseudo	SAK_1574	hypothetical protein	
GBS1219_1499	pseudo	GBS90503_1499	pseudo	GBS05108_1499	pseudo	GBS222_1518	pseudo	SAK_1819	putative phosphoketolase	Energy metabolism
GBS1219_1554	pseudo	GBS90503_1554	pseudo	GBS05108_1554	pseudo	GBS222_1567	pseudo	SAK_1866	hypothetical protein	
GBS1219_1555	pseudo	GBS90503_1555	pseudo	GBS05108_1555	pseudo	GBS222_1568	pseudo	SAK_1867	nitroreductase family protein	Unknown function
GBS1219_1556	pseudo	GBS90503_1556	pseudo	GBS05108_1556	pseudo	GBS222_1569	pseudo	SAK_1868	similar to MarR family transcriptional regulator	Regulatory functions
GBS1219_1567	pseudo	GBS90503_1567	pseudo	GBS05108_1567	pseudo	GBS222_1580	pseudo	SAK_1880	similar to two-component sensor histidine kinase SivA	Signal transduction
GBS1219_1587	pseudo	GBS90503_1587	pseudo	GBS05108_1587	pseudo	GBS222_1600	pseudo	SAK_1901	similar to bifunctional 2',3'-cyclic nucleotide 2'-phosphodiesterase/3'-nucleotidase precursor protein	Purines, pyrimidines, nucleosides, and nucleotides
GBS1219_1598	pseudo	GBS90503_1598	pseudo	GBS05108_1598	pseudo	GBS222_1611	pseudo	SAK_1912	Conserved hypothetical protein	Cell envelope
GBS1219_1608	pseudo	GBS90503_1608	pseudo	GBS05108_1608	pseudo	GBS222_1621	pseudo	SAK_1921	similar to two-component sensor histidine kinase	Signal transduction
GBS1219_1615	pseudo	GBS90503_1614	pseudo	GBS05108_1614	pseudo	GBS222_1628	pseudo	SAK_1928	hypothetical protein	
GBS1219_1651	pseudo	GBS90503_1650	pseudo	GBS05108_1650	pseudo	GBS222_1657	pseudo	SAK_1986	similar to bifunctional homocysteine S-methyltransferase/5,1-methylenetetrahydrofolate reductase protein	Amino acid biosynthesis
GBS1219_1668	pseudo	GBS90503_1667	pseudo	GBS05108_1667	pseudo	GBS222_1674	pseudo	SAK_2008	similar to phosphopentomutase	Purines, pyrimidines, nucleosides, and nucleotides
GBS1219_1709	pseudo	GBS90503_1708	pseudo	GBS05108_1708	pseudo	GBS222_1715	pseudo	SAK_2063	Membrane protein	Cell envelope
GBS1219_1745	pseudo	GBS90503_1744	pseudo	GBS05108_1744	pseudo	GBS222_1753	pseudo	SAK_2120	B3/4 domain-containing protein	Unknown function

B. Pseudogenes shared by ST260-261 strains resulting from convergent events

GBS1219_0045	pseudo	GBS90503_0045	pseudo	GBS05108_0045	pseudo	GBS222_0043	pseudo	SAK_0353	similar to unknown proteins	Cell envelope
GBS1219_0113	pseudo	GBS90503_0113	pseudo	GBS05108_0113	pseudo	GBS222_0111	pseudo	SAK_0429	Similar to sakacin A production response regulator	
GBS1219_0146	pseudo	GBS90503_0146	pseudo	GBS05108_0146	pseudo	GBS222_0143	pseudo	SAK_0462	putative hydrolase, haloacid dehalogenase-like family	Unknown function
GBS1219_0277	pseudo	GBS90503_0277	pseudo	GBS05108_0277	pseudo	GBS222_0278	pseudo	SAK_0179	L-2-hydroxyisocaproate dehydrogenase	Central intermediary metabolism
GBS1219_0301	pseudo	GBS90503_0301	pseudo	GBS05108_0301	pseudo	GBS222_0302	pseudo	SAK_0216	4-diphosphocytidyl-2-C-methyl-D-erythritol kinase	Biosynthesis of cofactors, prosthetic groups, and carriers
GBS1219_0339	pseudo	GBS90503_0339	pseudo	GBS05108_0339	pseudo	GBS222_0339	pseudo	SAK_0257	Similar to trehalose-specific PTS enzyme IIABC	Signal transduction

GBS1219_0367	pseudo	GBS90503_0367	pseudo	GBS05108_0367	pseudo	GBS222_0367	pseudo	SAK_0299	similar to efflux protein (truncated)	Transport and binding proteins
GBS1219_0818	pseudo	GBS90503_0818	pseudo	GBS05108_0818	pseudo	GBS222_0821	pseudo	SAK_1074	ABC transporter, substrate-binding protein	Transport and binding proteins
GBS1219_0868	pseudo	GBS90503_0868	pseudo	GBS05108_0868	pseudo	GBS222_0873	pseudo	SAK_1138	orotidine 5-phosphate decarboxylase	Cell envelope
GBS1219_0931	pseudo	GBS90503_0931	pseudo	GBS05108_0931	pseudo	GBS222_0937	pseudo	SAK_1191	AraC family transcriptional regulator	Regulatory functions
GBS1219_1072	pseudo	GBS90503_1072	pseudo	GBS05108_1072	pseudo	GBS222_1083	pseudo	SAK_1361	product="putative efflux ABC transporter, permease/ATP-binding protein	Transport and binding proteins
GBS1219_1073	pseudo	GBS90503_1073	pseudo	GBS05108_1073	pseudo	GBS222_1088	pseudo	SAK_1367	conserved hypothetical protein	Cell envelope
GBS1219_1087	pseudo	GBS90503_1087	pseudo	GBS05108_1087	pseudo	GBS222_1102	pseudo	SAK_1381	Surface protein	Cell envelope
GBS1219_1507	pseudo	GBS90503_1507	pseudo	GBS05108_1507	pseudo	GBS222_1526	pseudo	SAK_1827	hypothetical protein	
GBS1219_1565	pseudo	GBS90503_1565	pseudo	GBS05108_1565	pseudo	GBS222_1578	pseudo	SAK_1878	similar to malic enzyme ((S)-malate:NAD+ oxidoreductase)	Energy metabolism
GBS1219_1566	pseudo	GBS90503_1566	pseudo	GBS05108_1566	pseudo	GBS222_1579	pseudo	SAK_1879	similar to citrate, cation symporter (CCS) family protein	Transport and binding proteins
GBS1219_1578	pseudo	GBS90503_1578	pseudo	GBS05108_1578	pseudo	GBS222_1591	pseudo	SAK_1891	Neuraminidase	Unknown function
GBS1219_1580	pseudo	GBS90503_1580	pseudo	GBS05108_1580	pseudo	GBS222_1593	pseudo	SAK_1894	Similar to PTS enzyme IIB	Signal transduction
GBS1219_1655	pseudo	GBS90503_1654	pseudo	GBS05108_1654	pseudo	GBS222_1661	pseudo	SAK_1991	Putative Cell surface serine endopeptidase, similar to C5A peptidase	Protein fate
GBS1219_1753	pseudo	GBS90503_1752	pseudo	GBS05108_1752	pseudo	GBS222_1761	pseudo	SAK_2128	similar to unknown proteins	

C. Pseudogenes specific to ST260 strains

GBS1219_0006	pseudo	GBS90503_0006	pseudo	GBS05108_0006	pseudo	GBS222_0003	func	SAK_0003	Similar to putative transcription regulator	Unknown function
GBS1219_0061	pseudo	GBS90503_0061	pseudo	GBS05108_0061	pseudo	GBS222_0059	func	SAK_0369	similar to cysteine aminopeptidase C	Protein fate
GBS1219_0071	pseudo	GBS90503_0071	pseudo	GBS05108_0071	pseudo	GBS222_0069	func	SAK_0379	Similar to ABC transporter (permease)	Cell envelope
GBS1219_0073	pseudo	GBS90503_0073	pseudo	GBS05108_0073	pseudo	GBS222_0071	func	SAK_0381	Similar to two-component response regulator	Signal transduction
GBS1219_0087	pseudo	GBS90503_0087	pseudo	GBS05108_0087	pseudo	GBS222_0085	func	SAK_0395	similar to pyruvate formate-lyase activating enzyme	Energy metabolism
GBS1219_0151	pseudo	GBS90503_0151	pseudo	GBS05108_0151	pseudo	GBS222_0149	func	SAK_0468	Similar to two-component sensor histidine kinase	Signal transduction
GBS1219_0154	pseudo	GBS90503_0154	pseudo	GBS05108_0154	pseudo	GBS222_0152	func	SAK_0471	Similar to unknown protein (putative zinc finfer motif)	Unknown function
GBS1219_0171	pseudo	GBS90503_0171	pseudo	GBS05108_0171	pseudo	GBS222_0171	func	SAK_0054	similar to unknown transmembrane protein	Unknown function
GBS1219_0187	pseudo	GBS90503_0187	pseudo	GBS05108_0187	pseudo	GBS222_0187	func	SAK_0070	similar to unknown proteins	
GBS1219_0239	pseudo	GBS90503_0239	pseudo	GBS05108_0239	pseudo	GBS222_0240	func	SAK_0139	Similar to unknown proteins	
GBS1219_0264	pseudo	GBS90503_0264	pseudo	GBS05108_0264	pseudo	GBS222_0265	func	SAK_0166	Similar to ribose ABC transporter (binding protein)	Transport and binding proteins
GBS1219_0313	pseudo	GBS90503_0313	pseudo	GBS05108_0313	pseudo	GBS222_0314	func	SAK_0231	similar to hypothetical competence proteins	
GBS1219_0344	pseudo	GBS90503_0344	pseudo	GBS05108_0344	pseudo	GBS222_0344	func	SAK_0261	similar to other proteins (including putative transketolase)	Energy metabolism
GBS1219_0355	pseudo	GBS90503_0355	pseudo	GBS05108_0355	pseudo	GBS222_0355	func	SAK_0273	similar to unknown protein	0
GBS1219_0384	pseudo	GBS90503_0384	pseudo	GBS05108_0384	pseudo	GBS222_0384	func	SAK_0506	Similar to hypothetical transcriptional regulators	
GBS1219_0386	pseudo	GBS90503_0386	pseudo	GBS05108_0386	pseudo	GBS222_0386	func	SAK_0509	Similar to decarboxylase	Energy metabolism
GBS1219_0413	pseudo	GBS90503_0413	pseudo	GBS05108_0413	pseudo	GBS222_0413	func	SAK_0539	Putative aldose 1-epimerase	Energy metabolism
GBS1219_0438	pseudo	GBS90503_0438	pseudo	GBS05108_0438	pseudo	GBS222_0436	func	SAK_0559	similar to unknown proteins	Unknown function
GBS1219_0439	pseudo	GBS90503_0439	pseudo	GBS05108_0439	pseudo	GBS222_0437	func	SAK_0560	Similar to unknown proteins	Unknown function
GBS1219_0519	pseudo	GBS90503_0519	pseudo	GBS05108_0519	pseudo	GBS222_0517	func	SAK_0691	similar to unknown proteins	Transport and binding proteins
GBS1219_0544	pseudo	GBS90503_0544	pseudo	GBS05108_0544	pseudo	GBS222_0538	func		Hypothetical protein	
GBS1219_0548	pseudo	GBS90503_0548	pseudo	GBS05108_0548	pseudo	GBS222_0542	func	SAK_0788	similar to unknown proteins, putative transmembrane protein	Cell envelope
GBS1219_0556	pseudo	GBS90503_0556	pseudo	GBS05108_0556	contig end	GBS222_0556	func	SAK_0808	Conserved domain protein	
GBS1219_0559	pseudo	GBS90503_0559	pseudo	GBS05108_0559	pseudo	GBS222_0559	func	SAK_0811	Putative permease	Transport and binding proteins
GBS1219_0568	pseudo	GBS90503_0568	pseudo	GBS05108_0567	func	GBS222_0568	func	SAK_0820	weakly similar to PTS enzyme IIBC	Cell envelope
GBS1219_0571	pseudo	GBS90503_0571	pseudo	GBS05108_0571	pseudo	GBS222_0571	func	SAK_0823	similar to 2-keto-3-deoxygluconate kinase	Unknown function
GBS1219_0585	pseudo	GBS90503_0585	pseudo	GBS05108_0585	pseudo	GBS222_0586	func	SAK_0838	DNA-binding response regulator	Signal transduction
GBS1219_0587	pseudo	GBS90503_0587	pseudo	GBS05108_0587	pseudo	GBS222_0588	func	SAK_0840	similar to unknown proteins	Unknown function
GBS1219_0598	pseudo	GBS90503_0598	pseudo	GBS05108_0598	pseudo	GBS222_0599	func	SAK_0851	Similar to unknown proteins	Unknown function
GBS1219_0599	pseudo	GBS90503_0599	pseudo	GBS05108_0599	pseudo	GBS222_0600	func	SAK_0852	Similar to unknown proteins	Unknown function
GBS1219_0625	pseudo	GBS90503_0625	pseudo	GBS05108_0625	pseudo	GBS222_0625	func	SAK_0878	Similar to putative phosphoglycerate mutase	Unknown function
GBS1219_0645	pseudo	GBS90503_0645	pseudo	GBS05108_0645	pseudo	GBS222_0647	func	SAK_0899	similar to ABC transporter (ATP-binding protein)	Transport and binding proteins
GBS1219_0653	pseudo	GBS90503_0653	pseudo	GBS05108_0653	pseudo	GBS222_0655	func	SAK_0907	similar to competence protein CelB (ComEC)	Cellular processes
GBS1219_0686	pseudo	GBS90503_0686	pseudo	GBS05108_0686	pseudo	GBS222_0687	func	SAK_0939	conserved hypothetical protein	Cell envelope

GBS1219_0696	pseudo	GBS90503_0696	pseudo	GBS05108_0696	pseudo	GBS222_0697	func	SAK_0950	similar to uridine kinase	Purines, pyrimidines, nucleosides, and nucleotides
GBS1219_0722	pseudo	GBS90503_0722	pseudo	GBS05108_0722	pseudo	GBS222_0724	func	SAK_0976	similar to 1,4-alpha-glucan branching enzyme	Energy metabolism
GBS1219_0725	pseudo	GBS90503_0725	pseudo	GBS05108_0725	pseudo	GBS222_0727	func	SAK_0979	similar to glycogen synthase	Energy metabolism
GBS1219_0750	pseudo	GBS90503_0750	pseudo	GBS05108_0750	pseudo	GBS222_0752	func	SAK_1004	similar to acetoin dehydrogenase E3 component (dihydrolipoamide dehydrogenase)	Energy metabolism
GBS1219_0773	pseudo	GBS90503_0773	pseudo	GBS05108_0773	pseudo	GBS222_0775	func	SAK_1025	acetyltransferase	Unknown function
GBS1219_0777	pseudo	GBS90503_0777	pseudo	GBS05108_0777	pseudo	GBS222_0779	func	SAK_1029	similar to acetyltransferase (chloramphenicol ?)	Unknown function
GBS1219_0798	pseudo	GBS90503_0798	pseudo	GBS05108_0798	pseudo	GBS222_0801	func	SAK_1052	Similar to sugar (?) ABC transporter (permease)	Transport and binding proteins
GBS1219_0881	pseudo	GBS90503_0880	pseudo	GBS05108_0880	pseudo	GBS222_0887	func	SAK_1150	Similar to NADH-dependent oxidoreductase	Unknown function
GBS1219_1108	pseudo	GBS90503_1108	pseudo	GBS05108_1108	pseudo	GBS222_1123	func	SAK_1404	CapA domain-containing protein	Unknown function
GBS1219_1171	pseudo	GBS90503_1171	pseudo	GBS05108_1171	pseudo	GBS222_1182	func	SAK_1468	Hypothetical protein	
GBS1219_1186	pseudo	GBS90503_1186	pseudo	GBS05108_1186	pseudo	GBS222_1199	func	SAK_1482	Similar to preprotein translocase secA	Protein fate
GBS1219_1189	pseudo	GBS90503_1189	pseudo	GBS05108_1189	pseudo	GBS222_1202	func	SAK_1485	Hypothetical protein	Protein fate
GBS1219_1190	pseudo	GBS90503_1190	pseudo	GBS05108_1190	pseudo	GBS222_1206	func	SAK_1489	Similar to putative glycosyl transferase	Energy metabolism
GBS1219_1218	pseudo	GBS90503_1218	pseudo	GBS05108_1218	pseudo	GBS222_1236	func	SAK_1520	similar to transcriptional regulator	Regulatory functions
GBS1219_1272	pseudo	GBS90503_1272	pseudo	GBS05108_1272	pseudo	GBS222_1290	func	SAK_1575	Similar to branched-chain amino acid transporter	Transport and binding proteins
GBS1219_1274	pseudo	GBS90503_1274	pseudo	GBS05108_1274	pseudo	GBS222_1292	func	SAK_1577	hypothetical protein	Cellular processes
GBS1219_1302	pseudo	GBS90503_1302	pseudo	GBS05108_1302	pseudo	GBS222_1320	func	SAK_1606	Similar to TRK potassium uptake system protein TrkH	Transport and binding proteins
GBS1219_1342	pseudo	GBS90503_1342	pseudo	GBS05108_1342	pseudo	GBS222_1362	func	SAK_1647	similar to ABC transporter (ATP-binding protein)	Transport and binding proteins
GBS1219_1373	pseudo	GBS90503_1373	pseudo	GBS05108_1373	pseudo	GBS222_1391	func	SAK_1678	conserved hypothetical protein	Cell envelope
GBS1219_1385	pseudo	GBS90503_1385	pseudo	GBS05108_1385	pseudo	GBS222_1403	func	SAK_1690	Similar to unknown proteins	Unknown function
GBS1219_1438	pseudo	GBS90503_1438	pseudo	GBS05108_1438	pseudo	GBS222_1456	func	SAK_1744	Similar to X-prolyl dipeptidyl aminopeptidase	Protein fate
GBS1219_1446	pseudo	GBS90503_1446	pseudo	GBS05108_1446	pseudo	GBS222_1465	func		Conserved hypothetical protein	
GBS1219_1451	pseudo	GBS90503_1451	pseudo	GBS05108_1451	pseudo	GBS222_1470	func	SAK_1772	Similar to transcriptional regulator	Regulatory functions
GBS1219_1487	pseudo	GBS90503_1487	pseudo	GBS05108_1487	pseudo	GBS222_1506	func	SAK_1807	Hypothetical protein	
GBS1219_1494	pseudo	GBS90503_1494	pseudo	GBS05108_1494	pseudo	GBS222_1513	func	SAK_1814	similar to two-component response regulator	Signal transduction
GBS1219_1503	pseudo	GBS90503_1503	pseudo	GBS05108_1503	pseudo	GBS222_1522	func	SAK_1823	similar to L-xylulose kinase	Energy metabolism
GBS1219_1506	pseudo	GBS90503_1506	pseudo	GBS05108_1506	pseudo	GBS222_1525	func	SAK_1826	similar to glycerate dehydrogenase	Unknown function
GBS1219_1519	pseudo	GBS90503_1519	pseudo	GBS05108_1519	pseudo	GBS222_1538	func	SAK_1839	Hypothetical conserved membrane protein	Unknown function
GBS1219_1583	pseudo	GBS90503_1583	pseudo	GBS05108_1583	pseudo	GBS222_1596	func	SAK_1897	Streptococcal histidine triad family protein	Cellular processes
GBS1219_1602	pseudo	GBS90503_1602	pseudo	GBS05108_1602	pseudo	GBS222_1615	func		Bacteriocin processing peptidase / Bacteriocin export ABC transporter	
GBS1219_1605	pseudo	GBS90503_1605	pseudo	GBS05108_1605	pseudo	GBS222_1618	func	SAK_1918	similar to two-component response regulator	Signal transduction
GBS1219_1610	pseudo	GBS90503_1609	pseudo	GBS05108_1609	pseudo	GBS222_1623	func	SAK_1923	Similar to transcriptional regulator PhoU	Transport and binding proteins
GBS1219_1657	pseudo	GBS90503_1656	pseudo	GBS05108_1656	pseudo	GBS222_1663	func	SAK_1993	Similar to two-component sensor histidine kinase	Signal transduction
GBS1219_1672	pseudo	GBS90503_1671	pseudo	GBS05108_1671	pseudo	GBS222_1678	func	SAK_2012	Similar to transcriptional regulator (GntR family)	Regulatory functions
GBS1219_1711	pseudo	GBS90503_1710	pseudo	GBS05108_1710	pseudo	GBS222_1717	func	SAK_2065	similar to ornithine carbamoyltransferase	Amino acid biosynthesis
GBS1219_1712	pseudo	GBS90503_1711	pseudo	GBS05108_1711	pseudo	GBS222_1720	func	SAK_2068	Similar to osmoprotectant ABC transporter (ATP-binding protein)	Transport and binding proteins
GBS1219_1713	pseudo	GBS90503_1712	pseudo	GBS05108_1712	pseudo	GBS222_1721	func	SAK_2069	similar to osmoprotectant ABC transporter permease and substrate binding protein	Transport and binding proteins
GBS1219_1726	pseudo	GBS90503_1725	pseudo	GBS05108_1725	pseudo	GBS222_1734	func	SAK_2101	similar to membrane proteins of the MarC family	Cell envelope
GBS1219_1740	pseudo	GBS90503_1739	pseudo	GBS05108_1739	pseudo	GBS222_1748	func	SAK_2115	putative transmembrane protein similar to unknown protein	Transport and binding proteins

D. Pseudogenes specific to ST261 strains

GBS1219_0041	func	GBS90503_0041	func	GBS05108_0041	func	GBS222_0039	pseudo	SAK_0349	similar to transcriptional regulatory protein (N-terminal part)	
GBS1219_0046	func	GBS90503_0046	func	GBS05108_0046	func	GBS222_0044	pseudo	SAK_0354	similar to Similar to beta-glucoside specific PTS system enzyme IIBC	Signal transduction
GBS1219_0070	func	GBS90503_0070	func	GBS05108_0070	func	GBS222_0068	pseudo	SAK_0378		Transport and binding proteins
GBS1219_0089	func	GBS90503_0089	func	GBS05108_0089	func	GBS222_0087	pseudo	SAK_0397	transcriptional regulator, SorC family	Regulatory functions
GBS1219_0096	func	GBS90503_0096	func	GBS05108_0096	func	GBS222_0094	pseudo	SAK_0407	Putative ComFC protein	Cellular processes
GBS1219_0142	func	GBS90503_0142	func	GBS05108_0142	func	GBS222_0139	pseudo	SAK_0458	transcriptional repressor CopY	Regulatory functions

GBS1219_0188	func	GBS90503_0188	func	GBS05108_0188	func	GBS222_0188	pseudo	SAK_0071	Conserved hypothetical protein	Cell envelope
GBS1219_0205	func	GBS90503_0205	func	GBS05108_0205	func	GBS222_0205	pseudo	SAK_0087	similar to alcohol dehydrogenase	Energy metabolism
GBS1219_0281	func	GBS90503_0281	func	GBS05108_0281	func	GBS222_0282	pseudo	SAK_0183	Similar to unknown protein	Unknown function
GBS1219_0285	func	GBS90503_0285	func	GBS05108_0285	func	GBS222_0286	pseudo	SAK_0195	similar to unknown proteins	
GBS1219_0312	func	GBS90503_0312	func	GBS05108_0312	func	GBS222_0313	pseudo	SAK_0227	putative competence protein ComGB	Cellular processes
GBS1219_0330	func	GBS90503_0330	func	GBS05108_0330	func	GBS222_0330	pseudo	SAK_0248	Similar to two-component sensor histidine kinase	Signal transduction
GBS1219_0383	func	GBS90503_0383	func	GBS05108_0383	func	GBS222_0383	pseudo	SAK_0503	similar to unknown proteins	Biosynthesis of cofactors, prosthetic groups, and carriers
GBS1219_0400	func	GBS90503_0400	func	GBS05108_0400	func	GBS222_0400	pseudo	SAK_0526	Putative PTS system galactitol-specific enzyme IIC component, N terminal part	Signal transduction
GBS1219_0405	func	GBS90503_0405	func	GBS05108_0405	func	GBS222_0405	pseudo	SAK_0531	Putative transcriptional regulator, AraC family, N-terminal part	Regulatory functions
GBS1219_0409	func	GBS90503_0409	func	GBS05108_0409	func	GBS222_0409	pseudo	SAK_0535	Putative alpha-galactosidase, N-terminal	Energy metabolism
GBS1219_0410	func	GBS90503_0410	func	GBS05108_0410	func	GBS222_0410	pseudo		Galactokinase, N-terminal	
GBS1219_0412	func	GBS90503_0412	func	GBS05108_0412	func	GBS222_0412	pseudo	SAK_0538	UDP-glucose 4-epimerase	Energy metabolism
GBS1219_0417	func	GBS90503_0417	func	GBS05108_0417	func	GBS222_0417	pseudo	SAK_0546	hypothetical protein	
GBS1219_0444	func	GBS90503_0444	contig end	GBS05108_0444	contig end	GBS222_0442	pseudo	SAK_0566	biotin synthetase	Biosynthesis of cofactors, prosthetic groups, and carriers
GBS1219_0489	func	GBS90503_0489	func	GBS05108_0489	func	GBS222_0487	pseudo	SAK_0660	similar to cell wall muropeptide branching enzyme, FemAB family protein	Cell envelope
GBS1219_0529	func	GBS90503_0529	func	GBS05108_0529	func	GBS222_0527	pseudo	SAK_0714	similar to unknown proteins	
GBS1219_0545	func	GBS90503_0545	func	GBS05108_0545	func	GBS222_0539	pseudo	SAK_0785	Unknown	
GBS1219_0558	func	GBS90503_0558	func	GBS05108_0558	func	GBS222_0558	pseudo	SAK_0810		Transport and binding proteins
GBS1219_0572	func	GBS90503_0572	func	GBS05108_0572	func	GBS222_0573	pseudo	SAK_0825	similar to transcriptional regulator (GntR family)	Regulatory functions
GBS1219_0574	func	GBS90503_0574	func	GBS05108_0574	func	GBS222_0575	pseudo	SAK_0827	similar to glucuronate isomerase	Energy metabolism
GBS1219_0590	func	GBS90503_0590	func	GBS05108_0590	func	GBS222_0591	pseudo	SAK_0843	polar amino acid ABC transporter permease	Transport and binding proteins
GBS1219_0660	func	GBS90503_0660	func	GBS05108_0660	func	GBS222_0662	pseudo	SAK_0914	putative transcriptional antiterminator LicT family	Regulatory functions
GBS1219_0661	func	GBS90503_0661	func	GBS05108_0661	func	GBS222_0663	pseudo	SAK_0915	putative PTS system, beta-glucosides-specific IIABC components	Signal transduction
GBS1219_0681	func	GBS90503_0681	func	GBS05108_0681	func	GBS222_0683	pseudo	SAK_0935	similar to unknown proteins	Cell envelope
GBS1219_0682	func	GBS90503_0682	func	GBS05108_0682	func	GBS222_0684	pseudo	SAK_0936	putative glycosyl transferase, family 8	Energy metabolism
GBS1219_0713	func	GBS90503_0713	func	GBS05108_0713	func	GBS222_0715	pseudo	SAK_0967	Putative Acetyltransferase, GNAT family	Unknown function
GBS1219_0739	func	GBS90503_0739	func	GBS05108_0739	func	GBS222_0741	pseudo	SAK_0993	Putative acetyltransferase	Unknown function
GBS1219_0744	func	GBS90503_0744	func	GBS05108_0744	func	GBS222_0746	pseudo	SAK_0998	Putative magnesium transporter, CorA family	Transport and binding proteins
GBS1219_0766	func	GBS90503_0766	func	GBS05108_0766	func	GBS222_0768	pseudo	SAK_1020	CRISPR associated protein	Mobile and extrachromosomal element functions
GBS1219_0771	func	GBS90503_0771	func	GBS05108_0771	func	GBS222_0773	pseudo	SAK_1023	Internalin A	
GBS1219_0775	func	GBS90503_0775	func	GBS05108_0775	func	GBS222_0777	pseudo	SAK_1027	cation transporter HAD ATPase	Transport and binding proteins
GBS1219_0776	func	GBS90503_0776	func	GBS05108_0776	func	GBS222_0778	pseudo	SAK_1028	Unknown	
GBS1219_0810	func	GBS90503_0810	func	GBS05108_0810	func	GBS222_0813	pseudo	SAK_1066	Similar to other lipoprotein	Cell envelope
GBS1219_0857	func	GBS90503_0857	func	GBS05108_0857	func	GBS222_0861	pseudo		conserved hypothetical protein	Inorganic ion transport and metabolism
GBS1219_0870	func	GBS90503_0869	func	GBS05108_0869	func	GBS222_0874	pseudo	SAK_1139	similar to ABC transporter (ATP-binding protein)	Transport and binding proteins
GBS1219_0899	func	GBS90503_0899	func	GBS05108_0899	func	GBS222_0906	pseudo	SAK_1168	NADPH-dependent FMN reductase domain-containing protein	Unknown function
GBS1219_0905	func	GBS90503_0904	func	GBS05108_0904	func	GBS222_0911	pseudo	SAK_1173	Similar to drug-export protein	Transport and binding proteins
GBS1219_0908	func	GBS90503_0907	func	GBS05108_0907	func	GBS222_0914	pseudo	SAK_1176	short chain dehydrogenase/reductase family	Unknown function
GBS1219_0930	func	GBS90503_0930	func	GBS05108_0930	func	GBS222_0936	pseudo	SAK_1190	Cof-like hydrolase family protein	Unknown function
GBS1219_0969	func	GBS90503_0969	func	GBS05108_0969	func	GBS222_0977	pseudo	SAK_1233	Conserved hypothetical protein	Unknown function
GBS1219_0999	contig end	GBS90503_0999	contig end	GBS05108_0999	contig end	GBS222_1008	pseudo	SAK_1267	similar to purine nucleoside phosphorylase	Purines, pyrimidines, nucleosides, and nucleotides
GBS1219_1004	func	GBS90503_1004	func	GBS05108_1004	func	GBS222_1016	pseudo	SAK_1274	putative ABC transporter (ATP-binding protein)	Transport and binding proteins
GBS1219_1049	func	GBS90503_1049	func	GBS05108_1049	func	GBS222_1061	pseudo	SAK_1338	Putative amino acid transporter	Transport and binding proteins
GBS1219_1074	func	GBS90503_1074	func	GBS05108_1074	func	GBS222_1089	pseudo	SAK_1368	ABC transporter, ATP-binding/permease protein	Transport and binding proteins
GBS1219_1075	func	GBS90503_1075	func	GBS05108_1075	func	GBS222_1090	pseudo	SAK_1369	ABC transporter (ATP-binding protein)	Transport and binding proteins
GBS1219_1076	func	GBS90503_1076	func	GBS05108_1076	func	GBS222_1091	pseudo	SAK_1370	Putative acetyltransferase, GNAT family	Unknown function
GBS1219_1083	func	GBS90503_1083	func	GBS05108_1083	func	GBS222_1098	pseudo	SAK_1377	putative PTS system, fructose specific IIABC components	Signal transduction
GBS1219_1088	func	GBS90503_1088	func	GBS05108_1088	func	GBS222_1103	pseudo	SAK_1382	similar to 2-dehydropantoate 2-reductase	Biosynthesis of cofactors, prosthetic groups, and carriers
GBS1219_1098	func	GBS90503_1098	func	GBS05108_1098	func	GBS222_1113	pseudo	SAK_1394	Putative exported protein	Transport and binding proteins
GBS1219_1132	func	GBS90503_1132	func	GBS05108_1132	func	GBS222_1147	pseudo	SAK_1428		Transport and binding proteins
GBS1219_1174	func	GBS90503_1174	func	GBS05108_1174	func	GBS222_1185	pseudo	SAK_1471	Similar to glycerol (sugar)-3-phosphate transporter	Transport and binding proteins

GBS1219_1179	func	GBS90503_1179	func	GBS05108_1179	func	GBS222_1190	pseudo	SAK_1476		Transport and binding proteins
GBS1219_1181	func	GBS90503_1181	func	GBS05108_1181	contig end	GBS222_1192	pseudo	SAK_1478	similar to maltodextrin ABC transporter (permease)	Transport and binding proteins
GBS1219_1184	func	GBS90503_1184	func	GBS05108_1184	func	GBS222_1197	pseudo	SAK_1480	conserved hypothetical protein	
GBS1219_1185	func	GBS90503_1185	func	GBS05108_1185	func	GBS222_1198	pseudo	SAK_1481	Similar to putative glucosyl transferase	Cell envelope
GBS1219_1201	func	GBS90503_1201	func	GBS05108_1201	func	GBS222_1219	pseudo	SAK_1502	Similar to aminopeptidase	Protein fate
GBS1219_1214	func	GBS90503_1214	func	GBS05108_1214	func	GBS222_1232	pseudo	SAK_1516	Putative membrane protein	Cell envelope
GBS1219_1223	func	GBS90503_1223	func	GBS05108_1223	func	GBS222_1240	pseudo	SAK_1524	hypothetical protein	
GBS1219_1240	func	GBS90503_1240	func	GBS05108_1240	func	GBS222_1257	pseudo	SAK_1541	Similar to nickel ABC transporter, permease protein	Transport and binding proteins
GBS1219_1269	func	GBS90503_1269	func	GBS05108_1269	func	GBS222_1287	pseudo	SAK_1572	Similar to other proteins	
GBS1219_1290	func	GBS90503_1290	func	GBS05108_1290	func	GBS222_1308	pseudo	SAK_1594	similar to amino acid ABC transporter (ATP-binding protein)	Transport and binding proteins
GBS1219_1291	func	GBS90503_1291	func	GBS05108_1291	func	GBS222_1309	pseudo	SAK_1595	similar to amino acid ABC transporter (ATP-binding protein)	Transport and binding proteins
GBS1219_1292	func	GBS90503_1292	func	GBS05108_1292	func	GBS222_1310	pseudo	SAK_1596	similar to hydrophobic amino acid uptake ABC transporter, permease LivM	Transport and binding proteins
GBS1219_1322	func	GBS90503_1322	func	GBS05108_1322	func	GBS222_1342	pseudo	SAK_1626	Amidase family protein	Unknown function
GBS1219_1354	func	GBS90503_1354	func	GBS05108_1354	func	GBS222_1374	pseudo	SAK_1659	Similar to dihydroxyacetone kinase DAK1 domain-containing protein	Fatty acid and phospholipid metabolism
GBS1219_1357	func	GBS90503_1357	func	GBS05108_1357	func	GBS222_1375	pseudo	SAK_1662	similar to dihydroxyacetone kinase DAK1 domain-containing protein	Fatty acid and phospholipid metabolism
GBS1219_1410	func	GBS90503_1410	func	GBS05108_1410	func	GBS222_1428	pseudo	SAK_1716	similar to multidrug resistance protein	Transport and binding proteins
GBS1219_1428	func	GBS90503_1428	func	GBS05108_1428	func	GBS222_1446	pseudo	SAK_1734	DNA polymerase IV	DNA metabolism
GBS1219_1450	func	GBS90503_1450	func	GBS05108_1450	func	GBS222_1469	pseudo	SAK_1771	cyclopropane-fatty-acyl-phospholipid synthase	Fatty acid and phospholipid metabolism
GBS1219_1493	func	GBS90503_1493	func	GBS05108_1493	func	GBS222_1512	pseudo	SAK_1813	sensor histidine kinase DltS	Signal transduction
GBS1219_1505	func	GBS90503_1505	func	GBS05108_1505	func	GBS222_1524	pseudo	SAK_1825	Similar to galactitol-specific PTS enzyme IIC	Signal transduction
GBS1219_1535	func	GBS90503_1535	func	GBS05108_1535	func	GBS222_1554	pseudo	SAK_1856	putative Na+/H+ exchanger family protein	Transport and binding proteins
GBS1219_1543	func	GBS90503_1543	func	GBS05108_1543	func	GBS222_1562	pseudo	SAK_1864	Similar to transcriptional regulator (LacI family)	Regulatory functions
GBS1219_1545	func	GBS90503_1545	func	GBS05108_1545	func	GBS222_1564	pseudo			
GBS1219_1552	func	GBS90503_1552	func	GBS05108_1552	func	GBS222_1565	pseudo			
GBS1219_1570	func	GBS90503_1570	func	GBS05108_1570	func	GBS222_1583	pseudo	SAK_1883	Similar to glucan 1,6-alpha-glucosidase	Energy metabolism
GBS1219_1579	func	GBS90503_1579	func	GBS05108_1579	func	GBS222_1592	pseudo	SAK_1893	Similar to galactitol-specific PTS enzyme IIC	Signal transduction
GBS1219_1589	func	GBS90503_1589	func	GBS05108_1589	func	GBS222_1602	pseudo	SAK_1903	Similar to glutamyl-aminopeptidase (hypothetical)	Protein fate
GBS1219_1611	func	GBS90503_1610	func	GBS05108_1610	func	GBS222_1624	pseudo	SAK_1924	similar to phosphate ABC transporter (ATP-binding protein)	Transport and binding proteins
GBS1219_1620	func	GBS90503_1619	func	GBS05108_1619	func	GBS222_1633	pseudo	SAK_1933	Putative acetyltransferase, GNAT family	Unknown function
GBS1219_1624	func	GBS90503_1623	func	GBS05108_1623	func	GBS222_1637	pseudo		Conserved hypothetical protein	
GBS1219_1641	func	GBS90503_1640	func	GBS05108_1640	func	GBS222_1646	pseudo	SAK_1975	Putative ABC transporter (ATP-binding protein)	Transport and binding proteins
GBS1219_1647	func	GBS90503_1646	func	GBS05108_1646	func	GBS222_1653	pseudo	SAK_1983	CAMfactor	Cellular processes
GBS1219_1684	func	GBS90503_1683	func	GBS05108_1683	func	GBS222_1690	pseudo	SAK_2023		Unknown function

E. Pseudogenes specific to strain SS1219

GBS1219_0040	pseudo	GBS90503_0040	contig end	GBS05108_0040	contig end	GBS222_0036	func	SAK_0348	similar to NADH oxidase	Unknown function
GBS1219_0487	pseudo	GBS90503_0487	contig end	GBS05108_0487	contig end	GBS222_0485	func	SAK_0658	similar to Cell Wall Muropeptide Branching Enzyme	Cell envelope
GBS1219_0815	pseudo	GBS90503_0815	func	GBS05108_0815	func	GBS222_0818	func	SAK_1071	Similar to two-component response regulator	Signal transduction
GBS1219_0990	pseudo	GBS90503_0990	func	GBS05108_0990	func	GBS222_0999	func	SAK_1257	polysaccharide biosynthesis protein CpsG	Cell envelope
GBS1219_1100	pseudo	GBS90503_1100	func	GBS05108_1100	func	GBS222_1115	func	SAK_1396	Similar to carbamoyl-phosphate synthase, small subunit	Purines, pyrimidines, nucleosides, and nucleotides
GBS1219_1109	pseudo	GBS90503_1109	func	GBS05108_1109	func	GBS222_1124	func	SAK_1405	Similar to hypothetical thiamine biosynthesis protein ThiI	Biosynthesis of cofactors, prosthetic groups, and carriers
GBS1219_1575	pseudo	GBS90503_1575	func	GBS05108_1575	func	GBS222_1588	func	SAK_1888	Similar to tagatose-6-phosphate kinase	Energy metabolism

F.Pseudogenes specific to strains 90503 or/and 0518A

GBS1219_0567	func	GBS90503_0567	func	GBS05108_0568	pseudo	GBS222_0567	func	SAK_0819	similar to transcriptional regulator, LysR family	Regulatory functions
GBS1219_1758	contig end	GBS90503_1760	pseudo	GBS05108_1760	contig end				transposase	

G. Pseudogenes shared by strains SS1219 and 2-22 (convergence)

GBS1219_0845	pseudo	GBS90503_0845	func	GBS05108_0845	func	GBS222_0849	pseudo	SAK_1101	DNA processing protein DprA, putative	Cellular processes
GBS1219_1086	pseudo	GBS90503_1086	contig end	GBS05108_1086	contig end	GBS222_1101	pseudo	SAK_1380	beta-lactam resistance facto	Cell envelope

H. Pseudogenes shared by strains 90503 and 2-22 (convergence)

GBS1219_0655	func	GBS90503_0655	pseudo	GBS05108_0655	pseudo	GBS222_0657	pseudo	SAK_0909	Similar to transcriptional regulator (LacI family)	Regulatory functions

GBS1219_1023	func	GBS90503_1023	pseudo	GBS05108_1023	pseudo	GBS222_1035	pseudo	SAK_1293	putative hydrolytic protein	Unknown function

I. Pseudogenes in ST260 strains , deleted in strain 2-22

GBS1219_0019	pseudo	GBS90503_0019	pseudo	GBS05108_0019	pseudo			SAK_0322	hypothetical protein	conserved domain protein
GBS1219_0117	pseudo	GBS90503_0117	pseudo	GBS05108_0117	pseudo				hypothetical protein	
GBS1219_0424	pseudo	GBS90503_0424	pseudo	GBS05108_0424	pseudo				CRISPR-associated protein Cas4	
GBS1219_0537	pseudo	GBS90503_0537	pseudo	GBS05108_0537	pseudo				hypothetical protein	
GBS1219_0538	pseudo	GBS90503_0538	pseudo	GBS05108_0538	pseudo				putative conjugative transposon recombinase	
GBS1219_0539	pseudo	GBS90503_0539	pseudo	GBS05108_0539	pseudo				Conserved hypothetical protein	
GBS1219_0550	pseudo	GBS90503_0550	pseudo	GBS05108_0550	pseudo			SAK_0795	ABC transporter, ATP-binding protein CylA	Cellular processes
GBS1219_0552	pseudo	GBS90503_0552	pseudo	GBS05108_0552	pseudo			SAK_0797	CylE protein	Cellular processes
GBS1219_0553	pseudo	GBS90503_0553	pseudo	GBS05108_0553	pseudo			SAK_0805	hypothetical protein	
GBS1219_0684	pseudo	GBS90503_0684	pseudo	GBS05108_0684	pseudo			SAK_0937	hypothetical protein	
GBS1219_0773	pseudo	GBS90503_0773	pseudo	GBS05108_077	pseudo				acetyltransferase	
GBS1219_1143	pseudo	GBS90503_1143	pseudo	GBS05108_1143	pseudo			SAK_1439	sortase family protein	
GBS1219_1144	pseudo	GBS90503_1144	pseudo	GBS05108_1144	pseudo			SAK_1440	surface protein Spb1	
GBS1219_1631	pseudo	GBS90503_1630	pseudo	GBS05108_1630	pseudo				hypothetical protein	
GBS1219_1636	pseudo	GBS90503_1635	pseudo	GBS05108_1635	pseudo				hypothetical protein	

K. Pseudogenes in strain 2-22, deleted in ST260 strains

						GBS222_0236	pseudo		conserved hypothetical protein	
						GBS222_0238	pseudo		conserved hypothetical protein	
						GBS222_0544	pseudo		Surface protein	
						GBS222_0545	pseudo		cell surface protein	
						GBS222_0549	pseudo		Membrane spanning protein	
						GBS222_0550	pseudo		Nucleotide-binding protein	
						GBS222_0551	pseudo		hypothetical protein	
						GBS222_0552	pseudo		hypothetical protein	
						GBS222_0554	pseudo		Endopeptidase O	
						GBS222_0862	pseudo		hypothetical protein	
						GBS222_1084	pseudo	SAK_1362	ABC transporter (ATP-binding protein)	Transport and binding proteins
						GBS222_1086	pseudo	SAK_1364	Surface protein, 5 -nucleotidase family protein, LPXTG motif	Unknown function
						GBS222_1087	pseudo	SAK_1366	Putative NADP-specific glutamate dehydrogenase	Amino acid biosynthesis
						GBS222_1207	pseudo	SAK_1490	Similar to putative glycosyl transferase	Energy metabolism
						GBS222_1280	pseudo		hypothetical protein	
						GBS222_1423	pseudo	SAK_1709	hypothetical protein	
						GBS222_1464	pseudo		putative Prenyltransferase	Biosynthesis of cofactors, prosthetic groups, and carriers
						GBS222_1718	pseudo	SAK_2066	Similar to hypothetical two-component sensor histidine kinase	Signal transduction
						GBS222_1719	pseudo	SAK_2067	Similar to hypothetical two-component response regulator	Signal transduction

L. Genes deleted in ST261, not in ST260 strains

GBS1219_0020	func	GBS90503_0020	func	GBS05108_0020	func			SAK_0323	hypothetical protein containing a lipase domain	
GBS1219_0021	func	GBS90503_0021	func	GBS05108_0021	func			SAK_0324	hypothetical protein containing a lipase domain	
GBS1219_0232	func	GBS90503_0232	func	GBS05108_0232	func				hypothetical protein	
GBS1219_0318	func	GBS90503_0318	func	GBS05108_0318	func			SAK_0326		
GBS1219_0423	func	GBS90503_0423	func	GBS05108_0423	func					
GBS1219_0536	func	GBS90503_0536	func	GBS05108_0536	func				similar to Abortive infection protein AbiGI	
GBS1219_0551	func	GBS90503_0551	func	GBS05108_0551	func			SAK_0796	ABC transporter, permease protein CylB	
GBS1219_0608	func	GBS90503_0608	func	GBS05108_0608	func			SAK_0861	hypothetical protein	
GBS1219_0630	func	GBS90503_0630	func	GBS05108_0630	func			SAK_0883	putative lipoprotein	
GBS1219_0786	func	GBS90503_0786	func	GBS05108_0786	func			SAK_1039	hypothetical protein	
GBS1219_1055	func	GBS90503_1055	func	GBS05108_1055	func				hypothetical protein	
GBS1219_1141	func	GBS90503_1141	func	GBS05108_1141	func				sortase family protein, putative	
GBS1219_1142	func	GBS90503_1142	func	GBS05108_1142	func				hypothetical protein	
GBS1219_1222	func	GBS90503_1222	func	GBS05108_1222	func			SAK_1523	CAAX amino terminal protease family protein	
GBS1219_1355	func	GBS90503_1355	func	GBS05108_1355	func			SAK_1660	transcriptional regulator, putative	
GBS1219_1356	func	GBS90503_1356	func	GBS05108_1356	func			SAK_1661	hypothetical protein	
GBS1219_1405	func	GBS90503_1405	func	GBS05108_1405	func			SAK_1711	hypothetical protein	
GBS1219_1546	func	GBS90503_1546	func	GBS05108_1546	func				PTS system mannose/fructose/sorbose family IIC	
GBS1219_1547	func	GBS90503_1547	func	GBS05108_1547	func				PTS system mannose/fructose/sorbose family IIA subunit	

GBS1219_1548	func	GBS90503_1548	func	GBS05108_1548	func				PTS system mannose/fructose/sorbose family IIA subunit	
GBS1219_1549	func	GBS90503_1549	func	GBS05108_1549	func				PTS system mannose/fructose/sorbose family IIA subunit	
GBS1219_1550	func	GBS90503_1550	func	GBS05108_1550	func				3-ketoacyl-(acyl-carrier-protein) reductase	
GBS1219_1551	func	GBS90503_1551	func	GBS05108_1551	func				3-ketoacyl-(acyl-carrier-protein) reductase	
GBS1219_1632	func	GBS90503_1631	func	GBS05108_1631	func			SAK_1965	hypothetical protein	
GBS1219_1633	func	GBS90503_1632	func	GBS05108_1632	func			SAK_1966	ABC transporter, ATP-binding protein	
GBS1219_1637	func	GBS90503_1636	func	GBS05108_1636	func			SAK_1971	hypothetical protein	
GBS1219_1638	func	GBS90503_1637	func	GBS05108_1637	func			SAK_1972	PadR family transcriptional regulator	
GBS1219_1639	func	GBS90503_1638	func	GBS05108_1638	func			SAK_1973	acetyltransferase	
GBS1219_1640	func	GBS90503_1639	func	GBS05108_1639	func			SAK_1974	hypothetical protein	
GBS1219_1642	func	GBS90503_1641	func	GBS05108_1641	func			SAK_1976	hypothetical protein	
GBS1219_1757	func	GBS90503_1757	func	GBS05108_1757	func				hypothetical protein	

M. Genes deleted in ST260 strains

						GBS222_0148	func		DNA-binding response regulator	
						GBS222_0546	func		Putative transcriptional regulator, MarR family	
						GBS222_0547	func		Putative acyl-CoA thioesterase	
						GBS222_0548	func		Cyclic nucleotide-binding domain protein	
						GBS222_0572	func		similar to beta-glucuronidase	
						GBS222_0646	func		Similar to unknown proteins	
						GBS222_0709	func		Hypothetical protein	
						GBS222_0824	func		Hypothetical protein	
						GBS222_1009	func		similar to arsenate reductase (hypothetical)	
						GBS222_1010	func		similar to arsenate reductase (hypothetical)	
						GBS222_1012	func		hypothetical protein	
						GBS222_1085	func		similar to hypothetical transcriptional regulator	
						GBS222_1194	func		conserved hypothetical protein	
						GBS222_1203	func		Similar to unknown protein	
						GBS222_1204	func		Similar to hypothetical glycosyl transferase	
						GBS222_1205	func		Similar to hypothetical glycosyl transferase	
						GBS222_1208	func		Similar to putative glycosyl transferase	

N. Genes deleted in strain 90503, not in strain SS1219

GBS1219_1609	func					GBS222_1622	func	SAK_1922	similar to two-component response regulator (PhoB)	Signal transduction
