# Supplementary material for: Reductive evolution in Streptococcus agalactiae and the emergence of a host adapted lineage
Source: BMC Genomics. 2013 Apr 15;14:252. doi: 10.1186/1471-2164-14-252 (PMC3637634; doi:10.1186/1471-2164-14-252)
Supplement: Additional file 6: Table S6 — Lists the two-component systems and transcription factors annotated in the genome sequences of ST260 and 261 strains. [file 1471-2164-14-252-S6.pdf]

Table S6: Two-component systems and transcription factors annotated in the genomes of ST260 and ST261 strains. Inactivation by gene disruption (ps) or deletion (del) of genes encoding putative Two-Component Systems (TCS) and transcription factors identified in NEM316 and A909 genomes.

type	name	family	ortholog in NEM316	ortholog in A909	Description	2-22	SS1219	90-503
TCS		OmpR	gbs0121	SAK_0174	two-component response regulator	ps	ps	ps
TCS			gbs0122	SAK_0175	two-component sensor histidine kinase	+[1]	+	+
TCS			gbs0180	SAK_0248	two-component sensor histidine kinase	ps	+	+
TCS		LytT	gbs0181	SAK_0249	two-component response regulator	+	+	+
TCS			gbs0298	SAK_0380	two-component sensor histidine kinase	+	+	+
TCS		LuxR/GerR	gbs0299	SAK_0381	two-component response regulator	+	ps	ps
TCS			gbs0309	SAK_0391	two-component sensor histidine kinase	+	+	+
TCS		LuxR/GerR	gbs0310	SAK_0392	two-component response regulator	+	+	+
TCS		OmpR	gbs0429	SAK_0467	two-component response regulator	+	+	+
TCS			gbs0430	SAK_0468	two-component sensor histidine kinase	+	ps	ps
TCS	vncR	OmpR	gbs0597	SAK_0701	two-component response regulator VncR	del	del	del
TCS	vncS		gbs0598	SAK_0702	two-component sensor histidine kinase VncS	del	del	del
TCS		OmpR	gbs0685	SAK_0838	two-component response regulator	+	ps	ps
TCS	vicR	OmpR	gbs0741	SAK_0845	two-component response regulator	+	+	+
TCS	vicK		gbs0742	SAK_0846	two-component sensor histidine kinase	+	+	+
TCS	graR	OmpR	gbs0963	SAK_1071	two-component response regulator	+	ps	+
TCS	graS		gbs0964	SAK_1072	two-component sensor histidine kinase	+	+	+
TCS	ciaH		gbs1019	SAK_1079	two-component sensor histidine kinase	+	+	+
TCS	ciaR	OmpR	gbs1020	SAK_1080	two-component response regulator	+	+	+
TCS	lytR	LytT	gbs1051	SAK_1111	two-component response regulator	+	+	+
TCS	lytS		gbs1052	SAK_1112	two-component sensor histidine kinase LytS	+	+	+
TCS	relS		gbs1397	SAK_1358	two-component sensor histidine kinase	+	+	+
TCS	relR	OmpR	gbs1398	SAK_1359	two-component response regulator	+	+	+
TCS	covS		gbs1671	SAK_1638	two-component sensor histidine kinase	+	+	+
TCS	covR	OmpR	gbs1672	SAK_1639	two-component response regulator	+	+	+
TCS	dltS		gbs1834	SAK_1813	two-component sensor histidine kinase	ps	+	+
TCS	dltR	OmpR	gbs1835	SAK_1814	two-component response regulator	+	ps	ps
TCS	SivS		gbs1908	SAK_1880	two-component sensor histidine kinase	ps	ps	ps
TCS	SivR	GntR	gbs1909	SAK_1881	two-component response regulator	+	+	+
TCS			gbs1934	SAK_1906	two-component response regulator	+	+	+
TCS		AraC	gbs1935	SAK_1907	two-component sensor histidine kinase	+	+	+
TCS	rgfC		gbs1943	SAK_1917	two-component sensor histidine kinase (C-terminal part)	+	+	+
TCS	rgfA	LytT	gbs1944	SAK_1918	two-component response regulator	+	ps	ps
TCS	PhoR		gbs1947	SAK_1921	two-component sensor histidine kinase	ps	ps	ps
TCS	PhoB	OmpR	gbs1948	SAK_1922	two-component response regulator (PhoB)	+	+	+
TCS		OmpR	gbs2009	SAK_1992	two-component response regulator	+	+	+
TCS			gbs2010	SAK_1993	two-component sensor histidine kinase	+	ps	ps
TCS		OmpR	gbs2081	SAK_2061	two-component response regulator	del	del	del
TCS			gbs2082	SAK_2062	two-component sensor histidine kinase	del	del	del
TCS			gbs2086	SAK_2066	hypothetical two-component sensor histidine kinase	+	del	del
TCS			gbs2087	SAK_2067	hypothetical two-component response regulator	ps	del	del
			gbs0003	SAK_0003	putative transcription regulator	+	+	+
	hrcA		gbs0094	SAK_0145	transcription repressor of class I heat-shock (HrcA)	+	+	+
			gbs0118	SAK_0171	transcription repressor of ribose operon	+	+	+
			gbs0150	SAK_0217	transcriptional regulator	+	+	+
			gbs0168	SAK_0235	transcriptional regulator	+	+	+
			gbs0191	SAK_0259	hypothetical transcriptional antiterminator (BglG family)	+	+	+
			gbs0207	0	transcriptional regulator	+	+	+
			gbs0212	SAK_0279	transcriptional regulator	del	+	+
			gbs0230	SAK_0298	transcriptional regulator (Rgg like)	+	+	+
			gbs0250	SAK_0332	transcriptional regulator (TetR/AcrR family)	+	+	+
			gbs0267	SAK_0349	transcriptional regulatory protein (N-terminal part)	+	+	+
		DeoR	gbs0314	SAK_0396	transcriptional regulator (DeoR family)	+	+	+
			gbs0315	SAK_0397	hypothetical transcriptional regulator	ps	+	+
		MarR	gbs0330	SAK_0417	transcriptional regulator (MarR family)	+	+	+
			gbs0342	SAK_0429	sakacin A production response regulator [Streptococcus mutans] hypothetical	+	+	+
			gbs0355	SAK_0442	transcription regulator, hypothetical.	+	+	+
			gbs0420	SAK_0458	negative transcriptional regulator (copper transport operon)	ps	+	+
			gbs0427	SAK_0465	simiular to transcription regulator (Fur family)	+	+	+
			gbs0458	SAK_0506	hypothetical transcriptional regulators	+	+	+
			gbs0462	SAK_0511	transcriptional regulator (MerR family)	+	+	+
			gbs0468	SAK_0515	transcriptional regulator tetR-family	+	+	+
		AraC/XylS	gbs0469	SAK_0516	transcriptional regulator (AraC/XylS family)	del	del	del
			gbs0475	0	transcriptional regulator (phage related)	+	+	+
			gbs0546	SAK_0601	transcriptional regulator	+	+	+
			gbs0612	SAK_0717	membrane bound transcriptional regulator	+	+	+
			gbs0617	SAK_0770	putative transcriptional regulator (TetR/AcrR family) C-terminal part	+	+	+
			gbs0618	SAK_0721	putative transcriptional regulator (TetR/AcrR family) N-terminal part	del	del	del
			gbs0626	0	transcriptional regulator (C-terminal part)	+	+	+
			gbs0627	SAK_0775	transcriptional regulator (N-terminal part)	del	del	del
			gbs0666	SAK_0819	transcriptional regulator, LysR family	+	ps?	ps?
			gbs0672	SAK_0825	transcriptional regulator (GntR family)	ps	+	+
			gbs0680	SAK_0833	catabolite control protein A	+	+	+
			gbs0774	SAK_0879	transcriptional regulator	+	+	+
			gbs0804	SAK_0909	transcriptional regulator (LacI family)	ps	+	ps
			gbs0809	SAK_0914	transcription antiterminator	ps	+	+
		MarR	gbs0816	SAK_0921	transcriptional regulator, MarR family	ps	ps	ps
			gbs0823	SAK_0928	hypothetical transcription factor	+	+	+
			gbs0833	SAK_0938	transcriptional regulator	+	+	+
			gbs0848	SAK_0953	transcriptional repressor of the biotin operon	+	+	+
			gbs0928	SAK_1034	transcriptional regulator (GntR family)	+	+	+
			gbs0954	SAK_1063	transcriptional regulator (GntR family)	+	+	+
			gbs1022	SAK_1082	phosphate uptake regulatory protein	+	+	+
		AraC/XylS	gbs1173	SAK_1191	transcriptional regulator (AraC/XylS family)	ps	ps	ps
			gbs1196	SAK_1215	hypothetical transcriptional regulator	+	+	+

		gbs1201	SAK_1220	transcriptional regulator, GntR family	+	+	+
		gbs1221	0	repressor protein - phage associated	+	+	+
		gbs1249	0	transcriptional regulator (LysR family)	+	+	+
		gbs1327	0	transcriptional regulator (phage related)	+	+	+
		gbs1332	0	transcription antiterminator	+	+	+
		gbs1337	0	lactose repressor	+	+	+
		gbs1344	0	phage repressor-like protein	+	+	+
		gbs1381	SAK_1341	transcriptional regulator	+	+	+
		gbs1402	SAK_1363	hypothetical transcriptional regulator	+	+	+
		gbs1418	SAK_1379	transcription repressor of fructose operon FruR	+	+	+
	RofA	gbs1426	SAK_1389	transcription regulator RofA related	ps	ps	ps
		gbs1434	SAK_1397	pyrimidine biosynthetic operon repressor	+	+	+
		gbs1437	SAK_1400	transcriptional regulator (LysR/MarR family)	+	+	+
rogB	RofA	gbs1479	0	transcription regulator	+	+	+
		gbs1509	SAK_1474	transcriptional regulator (LacI family)	+	+	+
rgA	RofA	gbs1530	0	transcription regulator RofA related	+	+	+
		gbs1555	SAK_1520	transcriptional regulator	+	ps	ps
		gbs1582	SAK_1548	transcriptional regulator (LysR family)	+	+	+
		gbs1590	SAK_1557	metal-dependent transcriptional regulator	+	+	+
		gbs1666	SAK_1633	SWI/SNF family helicase	+	+	+
		gbs1693	SAK_1660	putative transcription regulator	del	+	+
		gbs1699	SAK_1667	transcriptional regulator	+	+	+
		gbs1719	SAK_1687	transcriptional regulator (CodY family)	+	+	+
		gbs1736	SAK_1704	transcriptional regulator (LacI family)	+	+	+
		gbs1749	0	transcription regulator	+	+	+
		gbs1761	SAK_1724	transcriptional regulators	+	+	+
		gbs1780	SAK_1743	transcriptional regulator (FNR/CRP family)	+	+	+
		gbs1793	SAK_1772	transcriptional regulator	+	ps	ps
		gbs1807	SAK_1786	transcriptional regulator, MerR/GlnR family	+	+	+
		gbs1815	SAK_1794	transcription repressor of purine operon PurR	+	+	+
		gbs1842	SAK_1821	Weakly transcriptional regulator (antiterminator)	+	+	+
		gbs1849	SAK_1828	transcriptional regulator (LacI family)	+	+	+
		gbs1866	SAK_1845	transcription regulator	+	+	+
		gbs1870	SAK_1849	transctiptional regulator CtsR	+	+	+
		gbs1882	SAK_1862	transcriptional regulator (Crp/Fnr family)	+	+	+
		gbs1884	SAK_1864	transcriptional regulator (LacI family)	ps	+	+
		gbs1897	SAK_1868	transcription regulator (MarR family)	ps	ps	ps
		gbs1923	SAK_1896	transcriptional regulator, DeoR family	+	+	+
		gbs1949	SAK_1923	transcriptional regulator PhoU	+	+	+
		gbs1958	SAK_1932	transcriptional regulators	+	+	+
		gbs1972	SAK_1948	transcriptional regulator (phage related)	ps	+	+
		gbs1975	SAK_1949	hypothetical transcriptional regulator	+	+	+
		gbs1994	SAK_1977	transcription regulator (N-terminal part)	+	+	+
		gbs2027	SAK_2012	transcriptional regulator (GntR family)	+	ps	ps
		gbs2055	SAK_2041	arginine repressor ArgR	+	+	+
		gbs2094	SAK_2074	transcriptional regulator (TetR/AcrR family)	+	+	+
		gbs2117	SAK_2116	transcriptional regulator	+	+	+
		gbs2119	SAK_2118	arginine repressor (ArgR)	+	+	+
		gbs2120	SAK_2119	transcriptional regulator (Crp/Fnr family)	+	+	+
			SAK_0531		ps	+	+
			SAK_0188		del	+	+
			SAK_0189		del	+	+
		gbs1990	SAK_1972		+	+	+
		gbs2065	SAK_2052		del	del	del
			SAK_2088		del	del	del
			SAK_2012		+	ps	ps
			SAK_1948		del	del	del

1: the gene is functional.
