# Supplementary material for: Reductive evolution in Streptococcus agalactiae and the emergence of a host adapted lineage
Source: BMC Genomics. 2013 Apr 15;14:252. doi: 10.1186/1471-2164-14-252 (PMC3637634; doi:10.1186/1471-2164-14-252)
Supplement: Additional file 7: Table S7 — Shows the results of Microarray expression analysis for strains 2-22 (ST261), SS1219 (ST260) and CF01173 (ST7). [file 1471-2164-14-252-S7.pdf]

Table S7: Microarray expression analysis of strains 2-22 (ST261), SS1219 (ST260) and CF01173 (ST7) (Excel file). Transcript levels in the three strains were compared to transcript levels in strain A909 grown in the same conditions of temperature (37°C for strain CF01173; 30°C for strains 2-22 and SS1219). Genes whose expression varied more than 2-fold compared to strain A909 are highlighted in red (up-regulated genes) and green (down-regulated genes). The transcripts originating from functional (funct) or inactivated (pseudo) genes are designated according to their nomenclature in strain A909. Only probes with 100% identity to A909, CF00173, 2-22 and SS1219 transcript sequences were taken into account in the data analysis. The number of probes for each transcript is indicated.

A909 ortholog	nb of probes	Fold-change in 2-22 vs A909		Fold-change in SS1219 vs A909		Fold-change in CF01173 vs A909		Functional Annotation	KEGG category
SAK_0001	4	0.77	funct	1.73	funct	1.49	func	chromosomal replication initiation protein	DNA metabolism
SAK_0002	1	0.68	funct	1.73	funct	1.18	func	DNA polymerase III subunit beta	DNA metabolism
SAK_0003	1	1.1	funct	1.65	pseudo	1.74	func	diacylglycerol kinase domain-containing protein	Unknown function
SAK_0004	3	1.05	funct	1.53	funct	1.53	func	hypothetical protein	conserved hypothetical protein
SAK_0006	3	0.73	funct	0.78	funct	1.05	func	GTP-dependent nucleic acid-binding protein EngD	Unknown function
SAK_0008	2	0.85	funct	1.56	funct	2.15	func	transcription-repair coupling factor	DNA metabolism
SAK_0009	1	0.92	funct	0.7	funct	1.1	func	S4 domain-containing protein	Unknown function
SAK_0010	1	0.63	funct	0.87	funct	1.94	func	hypothetical protein	conserved hypothetical protein
SAK_0012	3	0.62	funct	0.84	funct	1.85	func	hypothetical protein	conserved hypothetical protein
SAK_0014	5	1.37	funct	1.05	funct	1.19	func	hypoxanthine-guanine phosphoribosyltransferase	Purines, pyrimidines, nucleosides, and nucleotides
SAK_0015	2	1.1	funct	0.95	funct	1.04	func	cell division protein FtsH	Cellular processes
SAK_0050	1	1.14	funct	1.05	funct	0.94	func	PcsB protein	Cellular processes
SAK_0051	1	1.45	funct	0.77	funct	0.79	func	ribose-phosphate pyrophosphokinase	Purines, pyrimidines, nucleosides, and nucleotides
SAK_0052	7	0.83	funct	0.32	funct	0.97	func	aromatic amino acid aminotransferase	Amino acid biosynthesis
SAK_0053	3	1.01	funct	0.35	funct	1.13	func	DNA repair protein RecO	DNA metabolism
SAK_0055	2	1.57	funct	1.12	funct	0.89	func	putative glycerol-3-phosphate acyltransferase PlsX	Fatty acid and phospholipid metabolism
SAK_0056	1	1.21	funct	1.23	funct	0.88	func	acyl carrier protein	Fatty acid and phospholipid metabolism
SAK_0057	1	1.56	funct	7.41	funct	0.67	func	phosphoribosylaminoimidazole-succinocarboxamide synthase	Purines, pyrimidines, nucleosides, and nucleotides
SAK_0058	1	1.08	funct	3.96	funct	0.83	func	phosphoribosylformylglycinamidine synthase	Purines, pyrimidines, nucleosides, and nucleotides
SAK_0059	1	0.84	funct	3.49	funct	0.9	func	amidophosphoribosyltransferase	Purines, pyrimidines, nucleosides, and nucleotides
SAK_0060	2	0.93	funct	2.92	funct	0.89	func	phosphoribosylaminoimidazole synthetase	Purines, pyrimidines, nucleosides, and nucleotides
SAK_0062	2	0.94	funct	2.23	funct	0.85	func	acetyltransferase	Unknown function
SAK_0064	4	3.35	funct	6.26	funct	5.03	func	zoocin A	Cellular processes
SAK_0065	5	1.99	funct	2.04	funct	1.14	func	group B streptococcal surface immunogenic protein	Cell envelope
SAK_0066	1	1.18	funct	0.97	funct	0.74	func	N-acetylmannosamine-6-phosphate 2-epimerase	Central intermediary metabolism
SAK_0070	2	2.25	funct	0.85	pseudo	0.72	func	hypothetical protein	conserved hypothetical protein
SAK_0073	1	2.07	funct	1.34	funct	0.99	func	ROK family protein	Unknown function
SAK_0075	3	1.79	funct	1.59	funct	0.59	func	RpiR family phosphosugar-binding transcriptional regulator	Regulatory functions
SAK_0077	1	1.1	funct	5.69	funct	0.64	func	phosphoribosylaminoimidazole carboxylase catalytic subunit	Purines, pyrimidines, nucleosides, and nucleotides
SAK_0080	2	1.56	funct	2.08	funct	1.12	func	adenylosuccinate lyase	Purines, pyrimidines, nucleosides, and nucleotides
SAK_0082	1	1.35	funct	1.45	funct	1.19	func	Holliday junction DNA helicase RuvB	DNA metabolism
SAK_0084	4	1.31	funct	1.02	funct	0.91	func	MORN repeat-containing protein	Unknown function
SAK_0085	3	1.31	funct	1.1	funct	0.95	func	hypothetical protein	Unknown function
SAK_0086	3	0.08	funct	0.11	funct	0.83	func	bifunctional acetaldehyde-CoA/alcohol dehydrogenase	Energy metabolism
SAK_0087	1	0.09	pseudo	0.2	funct	0.28	func	alcohol dehydrogenase	Energy metabolism
SAK_0088	2	0.72	funct	0.73	funct	0.65	func	threonine synthase	Amino acid biosynthesis
SAK_0089	1	1.02	funct	1.09	funct	0.72	func	MATE efflux family protein	Transport and binding proteins
SAK_0090	2	0.39	funct	1.24	funct	0.8	func	30S ribosomal protein S10	Protein synthesis
SAK_0091	3	0.29	funct	0.95	funct	0.82	func	50S ribosomal protein L3	Protein synthesis
SAK_0092	4	0.32	funct	0.92	funct	0.77	func	50S ribosomal protein L4	Protein synthesis
SAK_0093	1	0.27	funct	0.74	funct	0.79	func	50S ribosomal protein L23	Protein synthesis
SAK_0094	2	0.29	funct	0.91	funct	0.85	func	50S ribosomal protein L2	Protein synthesis
SAK_0095	2	0.26	funct	1.03	funct	0.93	func	30S ribosomal protein S19	Protein synthesis
SAK_0096	4	0.28	funct	0.99	funct	0.93	func	50S ribosomal protein L22	Protein synthesis
SAK_0097	3	0.24	funct	1.09	funct	0.99	func	30S ribosomal protein S3	Protein synthesis
SAK_0098	3	0.24	funct	1.1	funct	1.04	func	50S ribosomal protein L16	Protein synthesis
SAK_0099	2	0.24	funct	0.86	funct	0.96	func	50S ribosomal protein L29	Protein synthesis
SAK_0100	2	0.25	funct	0.95	funct	1.07	func	30S ribosomal protein S17	Protein synthesis
SAK_0101	3	0.24	funct	1	funct	1.02	func	50S ribosomal protein L14	Protein synthesis
SAK_0102	3	0.23	funct	0.93	funct	0.99	func	50S ribosomal protein L24	Protein synthesis
SAK_0103	3	0.22	funct	1.08	funct	0.89	func	50S ribosomal protein L5	Protein synthesis
SAK_0104	1	0.22	funct	0.89	funct	0.95	func	30S ribosomal protein S14	Protein synthesis
SAK_0105	1	0.23	funct	0.97	funct	0.92	func	30S ribosomal protein S8	Protein synthesis
SAK_0106	3	0.21	funct	0.84	funct	0.87	func	50S ribosomal protein L6	Protein synthesis
SAK_0107	2	0.21	funct	0.89	funct	0.84	func	50S ribosomal protein L18	Protein synthesis
SAK_0108	2	0.21	funct	0.75	funct	0.91	func	30S ribosomal protein S5	Protein synthesis
SAK_0109	3	0.23	funct	0.88	funct	0.86	func	50S ribosomal protein L30	Protein synthesis
SAK_0110	2	0.18	funct	1.02	funct	0.88	func	50S ribosomal protein L15	Protein synthesis
SAK_0111	3	0.21	funct	1.46	funct	0.85	func	preprotein translocase subunit SecY	Protein fate
SAK_0112	2	0.39	funct	0.88	funct	0.71	func	adenylate kinase	Purines, pyrimidines, nucleosides, and nucleotides
SAK_0113	2	0.48	funct	0.71	funct	0.92	func	translation initiation factor IF-1	Protein synthesis
SAK_0114	3	0.51	funct	0.74	funct	0.91	func	30S ribosomal protein S13	Protein synthesis
SAK_0115	3	0.57	funct	0.87	funct	0.92	func	30S ribosomal protein S11	Protein synthesis

SAK_0116	5	0.59	funct	0.85	funct	0.98	func	DNA-directed RNA polymerase subunit alpha	Transcription
SAK_0117	3	0.66	funct	1	funct	1	func	50S ribosomal protein L17	Protein synthesis
SAK_0139	2	1.18	funct	1.01	pseudo	1.23	func	hypothetical protein	conserved hypothetical protein
SAK_0140	4	4.56	pseudo	0.55	pseudo	1.24	func	hypothetical protein	conserved hypothetical protein
SAK_0142	1	1.67	funct	0.92	funct	0.88	func	phosphoglycerate mutase family protein	Unknown function
SAK_0143	2	1.04	funct	1.09	funct	1.04	func	D-alanyl-D-alanine carboxypeptidase	Cell envelope
SAK_0144	3	0.92	funct	1.09	funct	1.09	func	mannosyl-glycoprotein endo-beta-N-acetylglucosamidase family protein	Cell envelope
SAK_0145	3	1.74	funct	1.33	funct	0.8	func	heat-inducible transcription repressor	Regulatory functions
SAK_0146	3	1.42	funct	1.35	funct	1.01	func	co-chaperone protein GrpE	Protein fate
SAK_0147	2	0.97	funct	0.85	funct	0.97	func	molecular chaperone DnaK	Protein fate
SAK_0148	3	0.85	funct	0.92	funct	1.25	func	co-chaperone protein DnaJ	Protein fate
SAK_0149	2	1.4	funct	0.89	funct	1.39	func	GntR family transcriptional regulator	Regulatory functions
SAK_0150	2	0.71	funct	1.87	funct	1.38	func	tRNA pseudouridine synthase A	Protein synthesis
SAK_0151	2	0.83	funct	1.9	funct	1.25	func	phosphomethylpyrimidine kinase	Biosynthesis of cofactors, prosthetic groups, and carriers
SAK_0152	3	0.88	funct	1.87	funct	1.29	func	hypothetical protein	Cell envelope
SAK_0153	2	0.83	funct	2.28	funct	1.29	func	hypothetical protein	conserved hypothetical protein TIGR01440
SAK_0154	3	0.99	funct	0.66	funct	0.98	func	hypothetical protein	Transport and binding proteins
SAK_0155	3	0.78	funct	1.16	funct	1.15	func	trigger factor	Protein fate
SAK_0156	1	1.16	funct	0.65	funct	1.01	func	DNA-directed RNA polymerase subunit delta	Transcription
SAK_0157	3	0.4	funct	0.78	funct	1.37	func	CTP synthetase	Purines, pyrimidines, nucleosides, and nucleotides
SAK_0158	3	0.87	funct	0.6	funct	1.05	func	hypothetical protein	conserved hypothetical protein
SAK_0160	4	4.75	funct	6.08	funct	1.43	func	deoxyuridine 5'-triphosphate nucleotidohydrolase	Purines, pyrimidines, nucleosides, and nucleotides
SAK_0161	6	1.63	funct	1.54	funct	0.75	func	DNA repair protein RadA	DNA metabolism
SAK_0162	3	0.46	funct	0.65	funct	0.79	func	carbonic anhydrase, putative	Central intermediary metabolism
SAK_0163	2	2.02	funct	0.41	funct	1.82	func	pyridine nucleotide-disulfide oxidoreductase	Unknown function
SAK_0165	2	1.16	funct	0.83	funct	1.05	func	glutamyl-tRNA synthetase	Protein synthesis
SAK_0166	3	1.86	funct	0.92	pseudo	0.32	func	ribose ABC transporter, ribose-binding protein	Transport and binding proteins
SAK_0167	3	1.78	funct	1.19	funct	0.28	func	ribose ABC transporter, permease protein	Transport and binding proteins
SAK_0168	1	2.15	funct	1.2	funct	0.25	func	ribose ABC transporter, ATP-binding protein	Transport and binding proteins
SAK_0169	2	1.73	funct	1.3	funct	0.37	func	D-ribose pyranase	Transport and binding proteins
SAK_0174	2	1.04	pseudo	0.92	pseudo	0.95	func	DNA-binding response regulator	Signal transduction
SAK_0175	2	0.79	funct	0.95	funct	1.01	func	sensor histidine kinase	Signal transduction
SAK_0176	1	15.44	funct	10.79	funct	0.66	func	argininosuccinate synthase	Amino acid biosynthesis
SAK_0177	3	13.05	funct	10.44	funct	0.84	func	argininosuccinate lyase	Amino acid biosynthesis
SAK_0178	3	1.4	funct	0.8	funct	0.68	func	fructose-bisphosphate aldolase	Energy metabolism
SAK_0179	2	1.46	pseudo	0.34	pseudo	0.79	func	L-2-hydroxyisocaproate dehydrogenase	Central intermediary metabolism
SAK_0180	4	0.69	funct	0.96	funct	0.98	func	50S ribosomal protein L28	Protein synthesis
SAK_0181	3	1.26	funct	0.91	funct	0.83	func	hypothetical protein	conserved hypothetical protein
SAK_0182	4	0.87	funct	1.01	funct	0.95	func	DAK2 domain-containing protein	Unknown function
SAK_0183	1	1.7	pseudo	1.45	funct	1.11	func	SPFH domain-containing protein/band 7 family protein	Unknown function
SAK_0193	2	0.64	funct	1.1	funct	1.07	func	amino acid ABC transporter, ATP-binding protein, putative	Transport and binding proteins
SAK_0194	1	0.71	funct	0.91	funct	0.87	func	His/Glu/Gln/Arg/opine amino acid ABC transporter amino acid-binding protein/permease	Transport and binding proteins
SAK_0196	1	1.02	funct	1.43	funct	1	func	undecaprenyl pyrophosphate phosphatase	Cellular processes
SAK_0197	1	0.88	funct	0.93	funct	0.93	func	adaptor protein	Regulatory functions
SAK_0198	3	0.89	funct	1.17	funct	0.89	func	glycosyl transferase family protein	Cell envelope
SAK_0199	2	0.8	funct	0.47	funct	0.86	func	FeS assembly ATPase SufC	Biosynthesis of cofactors, prosthetic groups, and carriers
SAK_0200	5	0.81	funct	0.57	funct	0.92	func	hypothetical protein	Biosynthesis of cofactors, prosthetic groups, and carriers
SAK_0201	5	0.9	funct	0.66	funct	0.96	func	cysteine desulfurase SufS	Biosynthesis of cofactors, prosthetic groups, and carriers
SAK_0202	2	0.81	funct	0.68	funct	0.94	func	NifU family SUF system FeS assembly protein	Biosynthesis of cofactors, prosthetic groups, and carriers
SAK_0203	1	1.08	funct	0.95	funct	1.14	func	hypothetical protein	Biosynthesis of cofactors, prosthetic groups, and carriers
SAK_0204	3	1.5	funct	1.94	funct	1.21	func	D-alanyl-D-alanine carboxypeptidase	Cell envelope
SAK_0205	2	1.27	funct	1.7	funct	0.96	func	D-alanyl-D-alanine carboxypeptidase	Cell envelope
SAK_0206	2	1.19	funct	0.43	pseudo	0.56	func	oligopeptide ABC transporter, oligopeptide-binding protein, putative	Transport and binding proteins
SAK_0207	2	0.85	funct	0.5	funct	0.78	func	oligopeptide ABC transporter, permease protein	Transport and binding proteins
SAK_0208	2	0.82	funct	0.5	funct	0.79	func	oligopeptide ABC transporter, permease protein OppC, putative	Transport and binding proteins
SAK_0209	1	0.73	funct	0.46	funct	0.83	func	oligopeptide ABC transporter, ATP-binding protein	Transport and binding proteins
SAK_0210	1	0.84	funct	0.48	funct	0.86	func	oligopeptide ABC transporter, ATP-binding protein	Transport and binding proteins
SAK_0216	6	1.03	pseudo	0.91	pseudo	0.62	func	4-diphosphocytidyl-2-C-methyl-D-erythritol kinase	Biosynthesis of cofactors, prosthetic groups, and carriers
SAK_0217	1	0.82	funct	0.94	funct	0.63	func	adc operon repressor AdcR	Regulatory functions
SAK_0218	2	0.88	funct	1.06	funct	0.61	func	zinc ABC transporter, ATP-binding protein	Transport and binding proteins
SAK_0219	2	0.77	funct	0.94	funct	0.55	func	zinc ABC transporter, permease protein	Transport and binding proteins
SAK_0220	1	2.42	funct	2.81	funct	0.84	func	hypothetical protein	conserved hypothetical protein
SAK_0221	2	0.94	funct	0.73	funct	1	func	tyrosyl-tRNA synthetase	Protein synthesis
SAK_0222	3	1.03	funct	1.05	funct	1.06	func	penicillin-binding protein 1B	Cell envelope
SAK_0223	2	1.33	funct	0.85	funct	0.69	func	DNA-directed RNA polymerase subunit beta	Transcription
SAK_0224	5	1.04	funct	1.24	funct	0.83	func	DNA-directed RNA polymerase subunit beta'	Transcription
SAK_0225	3	0.74	funct	0.45	funct	0.57	func	hypothetical protein	conserved hypothetical protein
SAK_0233	1	1.02	funct	0.69	funct	1.09	func	hypothetical protein	conserved hypothetical protein
SAK_0234	2	0.51	funct	0.71	funct	0.86	func	acetate kinase	Energy metabolism

SAK_0235	1	0.42	funct	0.41	funct	1.45	func	DNA-binding protein	Unknown function
SAK_0237	1	0.65	pseudo	0.7	pseudo	1.27	func	hypothetical protein	conserved hypothetical protein
SAK_0238	4	0.82	pseudo	0.43	pseudo	0.89	func	CAAX amino terminal protease family protein	Unknown function
SAK_0239	3	1.15	funct	0.76	funct	0.94	func	pyrroline-5-carboxylate reductase	Amino acid biosynthesis
SAK_0240	8	1.03	funct	0.68	funct	0.92	func	glutamyl aminopeptidase	Protein fate
SAK_0241	2	1.04	funct	0.93	pseudo	0.82	func	hypothetical protein	hypothetical protein
SAK_0242	2	0.75	funct	0.52	funct	0.9	func	hypothetical protein	conserved hypothetical protein
SAK_0243	3	0.58	funct	0.54	funct	0.9	func	thioredoxin family protein	Energy metabolism
SAK_0244	1	0.65	funct	0.66	funct	0.89	func	tRNA-binding domain-containing protein	Unknown function
SAK_0245	3	0.79	funct	0.46	funct	0.76	func	hypothetical protein	conserved hypothetical protein
SAK_0246	2	1.63	funct	1.17	funct	0.81	func	single-stranded DNA-binding protein	DNA metabolism
SAK_0247	3	1.02	funct	0.4	funct	0.51	func	HAD-superfamily hydrolase, subfamily IA, variant 3 family protein	Unknown function
SAK_0248	2	1.17	pseudo	0.51	funct	0.56	func	sensor histidine kinase	Signal transduction
SAK_0249	3	1.31	funct	0.73	funct	0.58	func	DNA-binding response regulator	Signal transduction
SAK_0250	3	0.16	funct	1.24	funct	0.9	func	hypothetical protein	Unknown function
SAK_0252	3	0.58	funct	1.9	funct	0.93	func	peptide/opine/nickel uptake ABC transporter substrate-binding protein	Transport and binding proteins
SAK_0257	2	0.1	pseudo	0.02	pseudo	0	func	PTS system, trehalose-specific IIBCA component	Signal transduction
SAK_0258	6	0.04	pseudo	0.02	pseudo	0.01	func	alpha amylase family protein	Energy metabolism
SAK_0259	1	0.65	funct	0.97	funct	0.84	func	PRD domain/PTS system IIA domain-containing protein	Regulatory functions
SAK_0260	1	1.16	funct	0.98	funct	0.88	func	PTS system lactose/cellobiose family IIB subunit	Signal transduction
SAK_0261	1	0.73	funct	0.88	funct	0.83	func	ascorbate-specific PTS system enzyme IIC	Signal transduction
SAK_0262	2	0.8	funct	0.91	pseudo	1.1	func	transketolase, N-terminal subunit, putative	Energy metabolism
SAK_0264	3	12.34	pseudo	6.58	pseudo	1.77	func	oxidoreductase, NAD-binding	Unknown function
SAK_0265	5	0.91	funct	1.07	funct	1.09	func	30S ribosomal protein S15	Protein synthesis
SAK_0266	3	1.09	funct	0.72	funct	0.8	func	polynucleotide phosphorylase/polyadenylase	Transcription
SAK_0267	2	0.79	funct	0.85	funct	0.83	func	hypothetical protein	conserved hypothetical protein
SAK_0268	1	0.75	funct	0.84	funct	1	func	serine O-acetyltransferase	Amino acid biosynthesis
SAK_0270	3	0.72	funct	0.91	funct	1.05	func	cysteinyl-tRNA synthetase	Protein synthesis
SAK_0271	2	0.69	funct	1.95	funct	0.85	func	hypothetical protein	conserved hypothetical protein
SAK_0272	2	1.17	funct	0.62	funct	1.12	func	RNA methyltransferase	Protein synthesis
SAK_0273	2	0.89	funct	0.68	pseudo	1.15	func	hypothetical protein	conserved hypothetical protein
SAK_0274	3	0.94	funct	0.46	funct	0.91	func	DegV family protein	Unknown function
SAK_0275	1	2.04	funct	1.16	funct	1.01	func	hypothetical protein	hypothetical protein
SAK_0276	3	0.54	funct	0.88	funct	0.94	func	50S ribosomal protein L13	Protein synthesis
SAK_0277	3	0.7	funct	1.7	funct	0.79	func	30S ribosomal protein S9	Protein synthesis
SAK_0290	1	8.16	funct	7.29	pseudo	1.22	func	hypothetical protein	conserved hypothetical protein
SAK_0298	4	1.58	funct	0.24	funct	1	func	Rgg/GadR/MutR family transcriptional regulator	Regulatory functions
SAK_0299	1	1.8	pseudo	0.34	pseudo	0.76	func	major facilitator family transporter	Transport and binding proteins
SAK_0300	2	0.48	0	0.4	funct	0.59	func	quaternary amine ABC transporter permease	Transport and binding proteins
SAK_0301	2	0.57	funct	0.41	funct	0.65	func	quaternary amine ABC transporter amino acid-binding protein	Transport and binding proteins
SAK_0302	3	0.77	funct	0.39	funct	0.58	func	quaternary amine ABC transporter permease	Transport and binding proteins
SAK_0303	1	0.8	funct	0.37	funct	0.52	func	quaternary amine ABC transporter ATP-binding protein	Transport and binding proteins
SAK_0327	1	1.05	funct	0.34	funct	2.16	func	acetyltransferase	Unknown function
SAK_0329	2	0.85	funct	0.27	funct	2.17	func	acetyltransferase	Unknown function
SAK_0335	1	1.03	funct	1.76	pseudo	1.71	func	hypothetical protein	conserved hypothetical protein
SAK_0336	1	0.68	funct	1.65	funct	1.77	func	PadR family transcriptional regulator	Regulatory functions
SAK_0337	2	2.08	funct	9.92	funct	0.86	func	hypothetical protein	conserved hypothetical protein
SAK_0338	3	1.24	funct	2.98	funct	1.63	func	N-acetylglucosamine-6-phosphate deacetylase	Central intermediary metabolism
SAK_0340	3	0.33	funct	0.58	funct	0.8	func	glycyl-tRNA synthetase subunit alpha	Protein synthesis
SAK_0341	2	0.26	funct	0.58	funct	0.85	func	acyl carrier protein phosphodiesterase, putative	Fatty acid and phospholipid metabolism
SAK_0342	3	0.3	funct	0.79	funct	0.84	func	glycyl-tRNA synthetase subunit beta	Protein synthesis
SAK_0345	1	1.17	pseudo	1.21	funct	0.95	func	glycerol kinase	Energy metabolism
SAK_0346	3	0.94	funct	1.1	pseudo	0.79	func	glycerol-3-phosphate oxidase	Energy metabolism
SAK_0347	2	0.98	funct	1.38	funct	0.74	func	glycerol uptake facilitator protein	Transport and binding proteins
SAK_0348	4	1.1	funct	1.75	pseudo	0.83	func	pyridine nucleotide-disulfide oxidoreductase	Unknown function
SAK_0350	2	1.41	funct	1.3	funct	0.63	func	transketolase	Energy metabolism
SAK_0351	1	1.09	funct	0.94	funct	0.75	func	hypothetical protein	conserved hypothetical protein
SAK_0352	2	1.17	funct	1.07	funct	0.86	func	ABC transporter, ATP-binding protein	Transport and binding proteins
SAK_0354	4	0.58	funct	0.36	funct	0.74	func	PTS system, IIBC component	Signal transduction
SAK_0355	3	1.04	funct	0.53	funct	1.15	func	gamma-glutamyl kinase	Amino acid biosynthesis
SAK_0356	2	0.96	funct	0.59	funct	1.02	func	gamma-glutamyl phosphate reductase	Amino acid biosynthesis
SAK_0357	3	1.19	funct	1.22	funct	1.66	func	S-adenosyl-methyltransferase MraW	Unknown function
SAK_0358	3	0.88	funct	1.51	funct	1.74	func	cell division protein FtsL, putative	Cellular processes
SAK_0359	4	1.05	funct	1.07	funct	1.31	func	penicillin-binding protein 2X	Cell envelope
SAK_0360	1	1.09	funct	1.35	funct	1.01	func	phospho-N-acetylmuramoyl-pentapeptide-transferase	Cell envelope
SAK_0361	3	1.64	funct	2.72	funct	1.58	func	DEAD-box ATP dependent DNA helicase	Transcription
SAK_0362	2	0.07	funct	0.97	funct	1.49	func	polar amino acid ABC transporter amino acid-binding protein	Transport and binding proteins
SAK_0363	2	0.09	funct	0.73	funct	1.38	func	polar amino acid ABC transporter permease	Transport and binding proteins
SAK_0364	3	0.11	funct	0.75	funct	1.13	func	polar amino acid ABC transporter ATP-binding protein	Transport and binding proteins
SAK_0365	2	0.93	funct	1.21	funct	0.93	func	hypothetical protein	conserved hypothetical protein
SAK_0366	4	0.83	funct	0.72	funct	0.92	func	thioredoxin-disulfide reductase	Energy metabolism
SAK_0367	2	1.12	funct	0.86	funct	0.97	func	nicotinate phosphoribosyltransferase	Biosynthesis of cofactors, prosthetic groups, and carriers
SAK_0368	3	1.15	funct	1.03	funct	0.98	func	NAD synthetase	Biosynthesis of cofactors, prosthetic groups, and carriers

SAK_0369	7	1.94	funct	0.65	pseudo	1.45	func	aminopeptidase C	Protein fate
SAK_0370	3	1.12	funct	1.49	funct	0.95	func	pencillin-binding protein	Cell envelope
SAK_0371	3	1.48	funct	1.54	funct	0.78	func	Holliday junction-specific endonuclease	DNA metabolism
SAK_0372	3	1.18	funct	1.02	funct	0.93	func	hypothetical protein	conserved hypothetical protein
SAK_0373	2	0.97	funct	0.91	funct	0.78	func	DivIVA domain-containing protein	Unknown function
SAK_0374	4	0.95	funct	1.82	funct	1.32	func	hypothetical protein	conserved hypothetical protein
SAK_0375	4	1.07	funct	1.78	funct	1.23	func	hypothetical protein	conserved hypothetical protein
SAK_0376	2	0.8	funct	0.52	funct	0.78	func	S-ribosylhomocysteinase	Cellular processes
SAK_0377	2	0.74	funct	1.39	funct	0.68	func	hypothetical protein	Unknown function
SAK_0378	1	0.34	pseudo	0.1	funct	0.08	func	ABC transporter, ATP-binding protein	Transport and binding proteins
SAK_0379	3	0.41	funct	0.15	pseudo	0.14	func	hypothetical protein	Cell envelope
SAK_0380	3	0.49	funct	0.26	funct	0.25	func	hypothetical protein	Signal transduction
SAK_0381	1	0.61	funct	0.42	pseudo	0.3	func	LuxR family DNA-binding response regulator	Signal transduction
SAK_0382	3	0.65	funct	0.36	funct	0.29	func	hypothetical protein	conserved hypothetical protein
SAK_0383	2	1.72	funct	1.51	funct	1.11	func	guanylate kinase	Purines, pyrimidines, nucleosides, and nucleotides
SAK_0384	2	1.38	funct	1.59	funct	1.08	func	DNA-directed RNA polymerase subunit omega	Transcription
SAK_0385	1	1.53	funct	1.11	funct	1.77	func	primosome assembly protein PriA	DNA metabolism
SAK_0386	1	1.49	funct	1.04	funct	1.35	func	methionyl-tRNA formyltransferase	Protein synthesis
SAK_0387	1	1.39	funct	1.19	funct	1.64	func	sun protein	Protein synthesis
SAK_0388	2	1.48	funct	1.6	funct	1.3	func	serine/threonine protein phosphatase Stp1	Regulatory functions
SAK_0389	1	1.39	funct	1.46	funct	1.4	func	serine/threonine protein kinase Stk1	Regulatory functions
SAK_0390	2	1.79	funct	0.99	funct	1.21	func	hypothetical protein	conserved hypothetical protein
SAK_0391	4	1.39	funct	1.09	funct	1.36	func	sensor histidine kinase	Signal transduction
SAK_0392	2	1.25	funct	1.1	funct	1.32	func	LuxR family DNA-binding response regulator	Signal transduction
SAK_0393	2	0.92	funct	0.77	funct	0.86	func	Cof-like hydrolase/peptidyl-prolyl cis-trans isomerase domain-containing protein	Unknown function
SAK_0394	2	1.15	funct	1.09	funct	0.7	func	hypothetical protein	Cellular processes
SAK_0395	2	0.54	funct	1.05	pseudo	0.95	func	pyruvate formate-lyase-activating enzyme, putative	Energy metabolism
SAK_0396	2	0.76	funct	1.35	funct	1.22	func	DeoR family transcriptional regulator	Regulatory functions
SAK_0397	3	0.72	pseudo	1.46	funct	1.15	func	SorC family transcriptional regulator	Regulatory functions
SAK_0399	1	1.24	funct	1.74	funct	0.66	func	PTS system lactose/cellobiose family IIB subunit	Signal transduction
SAK_0404	3	0.05	pseudo	0.59	pseudo	1.66	func	cysteine synthase/cystathionine beta-synthase family protein	Amino acid biosynthesis
SAK_0405	2	0.53	funct	0.85	funct	1.31	func	hypothetical protein	conserved hypothetical protein TIGR00257
SAK_0408	6	2.9	funct	2.21	funct	0.6	func	ribosomal subunit interface protein	Protein synthesis
SAK_0414	2	0.94	funct	1.33	funct	0.81	func	aspartate kinase	Amino acid biosynthesis
SAK_0415	1	1.39	funct	2.14	funct	1	func	HAD-superfamily hydrolase, subfamily IA, variant 3 family protein	Unknown function
SAK_0416	3	0.55	funct	0.45	funct	2.09	func	enoyl-CoA hydratase	Fatty acid and phospholipid metabolism
SAK_0417	4	0.48	funct	1.11	funct	1.75	func	MarR family transcriptional regulator	Regulatory functions
SAK_0418	4	0.42	funct	1.15	funct	1.94	func	3-oxoacyl-(acyl carrier protein) synthase III	Fatty acid and phospholipid metabolism
SAK_0419	3	0.6	funct	1.29	funct	1.68	func	acyl carrier protein	Fatty acid and phospholipid metabolism
SAK_0420	3	0.51	funct	1.01	funct	1.71	func	enoyl-(acyl-carrier-protein) reductase II	Fatty acid and phospholipid metabolism
SAK_0421	3	0.48	funct	0.86	funct	1.5	func	acyl-carrier-protein S-malonyltransferase	Fatty acid and phospholipid metabolism
SAK_0422	2	0.65	funct	1.16	funct	1.46	func	3-ketoacyl-(acyl-carrier-protein) reductase	Fatty acid and phospholipid metabolism
SAK_0423	2	0.82	funct	1.25	funct	1.22	func	3-oxoacyl-(acyl carrier protein) synthase II	Fatty acid and phospholipid metabolism
SAK_0424	3	0.63	funct	1.09	funct	1.27	func	acetyl-CoA carboxylase biotin carboxyl carrier protein subunit	Fatty acid and phospholipid metabolism
SAK_0425	2	0.78	funct	1.26	funct	1.3	func	(3R)-hydroxymyristoyl-ACP dehydratase	Fatty acid and phospholipid metabolism
SAK_0426	1	0.64	funct	1.22	funct	1.18	func	acetyl-CoA carboxylase biotin carboxylase subunit	Fatty acid and phospholipid metabolism
SAK_0427	12	0.66	funct	1.15	funct	1.13	func	acetyl-CoA carboxylase subunit beta	Fatty acid and phospholipid metabolism
SAK_0428	2	0.73	funct	1.27	funct	1.18	func	acetyl-CoA carboxylase subunit alpha	Fatty acid and phospholipid metabolism
SAK_0429	2	0.92	pseudo	1.32	pseudo	1.4	func	hypothetical protein	conserved hypothetical protein
SAK_0430	2	0.48	funct	0.54	funct	0.84	func	seryl-tRNA synthetase	Protein synthesis
SAK_0431	1	0.52	pseudo	0.7	pseudo	0.91	func	hypothetical protein	Cell envelope
SAK_0432	3	0.81	funct	0.91	funct	0.9	func	hypothetical protein	conserved hypothetical protein
SAK_0433	3	0.39	funct	1.32	funct	0.62	func	PTS system mannose/fructose/sorbose family IIDsubunit	Signal transduction
SAK_0434	3	0.33	funct	0.86	funct	0.59	func	PTS system lactose/cellobiose family IIC subunit	Signal transduction
SAK_0435	3	0.31	funct	0.66	funct	0.56	func	PTS system mannose/fructose/sorbose family IIA subunit	Signal transduction
SAK_0436	2	0.99	funct	2.19	funct	1.26	func	Cof-like hydrolase	Unknown function
SAK_0437	4	1.47	funct	1.81	funct	0.81	func	hypothetical protein	Cell envelope
SAK_0439	3	0.82	funct	1.83	funct	0.79	func	AzgA family purine transporter	Transport and binding proteins
SAK_0440	3	0.8	funct	1.29	funct	1.14	func	hypothetical protein	conserved hypothetical protein TIGR00150
SAK_0441	2	0.51	funct	1.15	funct	1.41	func	acetyltransferase	Unknown function
SAK_0442	1	0.39	funct	1.1	pseudo	1.59	func	hypothetical protein	Regulatory functions
SAK_0443	2	1.21	funct	1.33	funct	1.09	func	hypothetical protein	conserved hypothetical protein
SAK_0444	2	1.37	funct	1.3	funct	1.14	func	HIT family protein	Unknown function
SAK_0446	2	2.07	funct	2.18	funct	1.26	func	ABC transporter, ATP-binding protein	Transport and binding proteins
SAK_0447	2	1.74	funct	1.87	funct	1.43	func	ABC transporter, permease protein EcsB, putative	Transport and binding proteins
SAK_0448	2	1.36	funct	1.65	funct	1.12	func	hypothetical protein	conserved hypothetical protein
SAK_0449	3	1.01	funct	1.62	funct	1.08	func	tRNA (guanine-N(7)-)-methyltransferase	Protein synthesis

SAK_0451	3	0.94	funct	1.4	funct	0.83	func	hypothetical protein	conserved hypothetical protein
SAK_0452	2	0.57	funct	1.5	funct	0.9	func	transcription elongation factor NusA	Transcription
SAK_0453	3	0.45	funct	1.42	funct	1.03	func	hypothetical protein	Unknown function
SAK_0454	4	0.49	funct	1.38	funct	0.96	func	hypothetical protein	Protein synthesis
SAK_0455	2	0.53	funct	1.3	funct	0.87	func	translation initiation factor IF-2	Protein synthesis
SAK_0456	3	0.59	funct	1.3	funct	1.06	func	ribosome-binding factor A	Transcription
SAK_0457	2	0.78	funct	0.96	funct	1.82	func	GDXG lipolytic enzyme family protein	Unknown function
SAK_0458	1	6.79	pseudo	1.29	funct	0.71	func	transcriptional repressor CopY	Regulatory functions
SAK_0459	3	3.51	funct	1.1	funct	1.17	func	copper-translocating P-type ATPase	Cellular processes
SAK_0460	2	3.56	funct	1.01	funct	1.24	func	copper transporter, copper-binding protein, putative	Cellular processes
SAK_0461	2	1.63	funct	0.62	funct	0.98	func	hypothetical protein	Cell envelope
SAK_0463	6	1.71	funct	0.94	funct	1.17	func	DNA polymerase I	DNA metabolism
SAK_0464	2	1.25	funct	0.95	funct	1.88	func	CoA-binding domain-containing protein	Unknown function
SAK_0465	3	1.57	funct	1.32	funct	0.83	func	FUR family transcriptional regulator	Regulatory functions
SAK_0469	1	1.25	funct	1.04	funct	0.68	func	hypothetical protein	Cell envelope
SAK_0470	1	0.39	funct	0.74	funct	0.79	func	queuine tRNA-ribosyltransferase	Protein synthesis
SAK_0471	1	0.69	funct	1.19	pseudo	0.5	func	hypothetical protein	Unknown function
SAK_0472	1	0.96	funct	0.86	funct	0.51	func	BioY family protein	Unknown function
SAK_0473	4	2.4	funct	0.98	funct	0.86	func	metallo-beta-lactamase family protein	Unknown function
SAK_0474	1	2.75	funct	1.42	funct	0.9	func	cytidine/deoxycytidylate deaminase family protein	Unknown function
SAK_0475	3	0.88	funct	0.67	funct	1.01	func	glucose-6-phosphate isomerase	Energy metabolism
SAK_0476	2	3.19	funct	1.23	pseudo	1	func	5-formyltetrahydrofolate cyclo-ligase family protein	Unknown function
SAK_0477	3	3.41	funct	1.39	funct	1.04	func	rhomboid family protein	Unknown function
SAK_0478	4	1.28	funct	1.81	funct	1.71	func	bmp family protein	Cell envelope
SAK_0479	1	1.41	funct	1.54	funct	0.9	func	UTP-glucose-1-phosphate uridylyltransferase	Cell envelope
SAK_0480	1	1.2	funct	1.59	funct	0.73	func	NAD(P)H-dependent glycerol-3-phosphate dehydrogenase	Fatty acid and phospholipid metabolism
SAK_0481	3	1.12	funct	2.89	funct	1.23	func	ribonuclease P	Transcription
SAK_0482	6	1.04	funct	3.5	funct	1.57	func	membrane protein oxaA, putative	Cell envelope
SAK_0483	4	0.86	funct	3.22	funct	1.75	func	R3H domain-containing protein	Unknown function
SAK_0492	2	1.59	funct	0.79	funct	0.58	func	hypothetical protein	conserved hypothetical protein
SAK_0494	4	1.07	funct	1.29	funct	1.08	func	23S rRNA (uracil-5-)-methyltransferase RumA	Protein synthesis
SAK_0495	1	0.73	funct	1.03	funct	1.37	func	hypothetical protein	conserved hypothetical protein
SAK_0496	1	0.77	funct	0.71	funct	1.3	func	acetyltransferase	Unknown function
SAK_0498	1	0.85	funct	0.95	funct	1.94	func	glycosyl transferase, group 2 family protein	Cell envelope
SAK_0499	1	1.12	funct	0.77	funct	1.35	func	ribonucleotide-diphosphate reductase subunit beta	Purines, pyrimidines, nucleosides, and nucleotides
SAK_0501	2	1.28	funct	0.74	funct	1.72	func	ribonucleotide-diphosphate reductase subunit alpha	Purines, pyrimidines, nucleosides, and nucleotides
SAK_0502	1	1.18	funct	0.71	funct	2.62	func	hypothetical protein	conserved hypothetical protein
SAK_0503	1	1.2	pseudo	0.7	funct	2.7	func	pyridoxamine 5'-phosphate oxidase family protein	Biosynthesis of cofactors, prosthetic groups, and carriers
SAK_0506	3	3.15	funct	3.25	pseudo	1.87	func	hypothetical protein	conserved hypothetical protein
SAK_0509	1	3.5	funct	3.11	pseudo	1.62	func	4-carboxymuconolactone decarboxylase, putative	Energy metabolism
SAK_0510	1	5.47	pseudo	3.1	pseudo	1.62	func	cupin domain-containing protein	Unknown function
SAK_0513	3	4.82	funct	2.37	funct	1.16	func	aldo/keto reductase family oxidoreductase	Unknown function
SAK_0514	2	2.56	funct	1.18	funct	1.11	func	cation efflux transporter	Transport and binding proteins
SAK_0520	1	0.89	funct	2.71	funct	1.4	func	hypothetical protein	conserved hypothetical protein
SAK_0521	1	1.12	funct	2.54	funct	1.43	func	hypothetical protein	conserved hypothetical protein
SAK_0522	3	0.98	funct	2.6	funct	1.49	func	hypothetical protein	conserved hypothetical protein
SAK_0523	1	1.4	funct	1.13	funct	0.56	func	PTS system IIA domain-containing protein	Unknown function
SAK_0526	1	2.18	pseudo	0.38	funct	0.72	func	PTS system, galactitol-specific IIC component, putative	Signal transduction
SAK_0530	1	9.2	funct	0.51	funct	0.89	func	PTS system, galactitol-specific IIB component, putative	Signal transduction
SAK_0531	2	2.34	pseudo	1.15	funct	0.65	func	AraC family transcriptional regulator	Regulatory functions
SAK_0542	1	0.42	funct	0.48	pseudo	0.33	func	aldose 1-epimerase	Energy metabolism
SAK_0543	2	0.83	funct	0.57	funct	0.83	func	hypothetical protein	conserved hypothetical protein
SAK_0544	1	0.81	funct	1.16	funct	0.7	func	hypothetical protein	conserved hypothetical protein
SAK_0545	2	0.36	funct	0.53	funct	0.84	func	acetyltransferase	Unknown function
SAK_0546	1	0.3	pseudo	0.67	funct	0.92	func	hypothetical protein	hypothetical protein
SAK_0547	2	0.38	funct	0.55	funct	0.71	func	valyl-tRNA synthetase	Protein synthesis
SAK_0549	4	2.11	funct	1.66	funct	1.04	func	Gfo/Idh/MocA family oxidoreductase	Unknown function
SAK_0550	1	0.8	pseudo	0.45	pseudo	0.73	func	CorA family metal ion transporter	Transport and binding proteins
SAK_0551	1	1.28	funct	1.31	funct	0.81	func	hypothetical protein	conserved hypothetical protein
SAK_0552	2	6.88	funct	3.92	funct	0.78	func	asparagine synthetase AsnA	Amino acid biosynthesis
SAK_0553	1	1.53	funct	1.63	funct	1.11	func	hypothetical protein	conserved hypothetical protein
SAK_0554	2	2.73	funct	3.62	funct	1.51	func	methyltransferase, putative	Unknown function
SAK_0555	2	1.97	funct	2.68	funct	1.49	func	phosphopantetheine adenylyltransferase	Biosynthesis of cofactors, prosthetic groups, and carriers
SAK_0556	3	1.63	pseudo	1.68	pseudo	1.29	func	hypothetical protein	conserved hypothetical protein
SAK_0557	3	1.56	pseudo	0.62	pseudo	1.02	func	Ser/Thr protein phosphatase family protein	Unknown function
SAK_0558	2	0.68	funct	0.84	funct	1.21	func	hypothetical protein	conserved hypothetical protein
SAK_0559	1	0.81	funct	0.84	pseudo	0.93	func	radical SAM protein	Unknown function
SAK_0560	1	0.91	funct	0.92	pseudo	0.85	func	VanZ family protein	Unknown function
SAK_0561	2	0.36	funct	0.66	funct	0.75	func	ABC transporter, ATP-binding/permease protein	Transport and binding proteins
SAK_0562	2	0.34	funct	0.77	funct	0.83	func	ABC transporter, ATP-binding/permease protein	Transport and binding proteins
SAK_0563	1	0.79	funct	0.9	funct	0.78	func	anthranilate synthase component II	Amino acid biosynthesis
SAK_0570	3	0.73	funct	0.59	funct	0.78	func	endonuclease III	DNA metabolism
SAK_0572	3	0.71	funct	0.42	funct	0.74	func	hypothetical protein	conserved hypothetical protein
SAK_0575	2	0.98	funct	0.8	funct	1.04	func	GTP-binding protein TypA	Unknown function
SAK_0576	2	0.95	funct	1.12	funct	0.77	func	hypothetical protein	conserved hypothetical protein
SAK_0577	2	1.19	funct	0.69	funct	1.09	func	UDP-N-acetylmuramoyl-L-alanyl-D-glutamate synthetase	Cell envelope

SAK_0578	1	0.93	funct	0.94	funct	1.93	func	undecaprenyldiphospho-muramoylpentapeptide beta-N-acetylglucosaminyltransferase	Cell envelope
SAK_0579	1	0.93	funct	0.76	funct	2.24	func	cell division protein DivIB	Cellular processes
SAK_0580	3	1.08	funct	1.55	funct	1.22	func	cell division protein FtsA	Cellular processes
SAK_0581	3	1.14	funct	1.34	funct	1.41	func	cell division protein FtsZ	Cellular processes
SAK_0582	3	0.88	funct	1.04	funct	1.56	func	hypothetical protein	conserved hypothetical protein TIGR00044
SAK_0583	3	0.91	funct	1.06	funct	1.52	func	hypothetical protein	conserved hypothetical protein
SAK_0584	3	0.88	funct	1.05	funct	1.83	func	YggT family protein	Unknown function
SAK_0585	2	0.88	funct	0.86	funct	1.48	func	S4 domain-containing protein	Unknown function
SAK_0586	2	1.04	funct	0.68	funct	1.21	func	cell division protein DivIVA, putative	Cellular processes
SAK_0587	3	0.45	funct	0.93	funct	0.68	func	isoleucyl-tRNA synthetase	Protein synthesis
SAK_0588	4	1.14	funct	0.22	funct	0.89	func	hypothetical protein	conserved hypothetical protein
SAK_0590	3	1.55	funct	0.84	funct	0.7	func	ATP-dependent Clp protease, ATP-binding subunit ClpE	Protein fate
SAK_0591	2	1.31	funct	1.1	funct	0.65	func	hypothetical protein	conserved hypothetical protein
SAK_0592	2	2.64	funct	5.79	funct	0.81	func	polar amino acid ABC transporter permease	Transport and binding proteins
SAK_0593	2	2.91	funct	5.9	funct	0.78	func	polar amino acid ABC transporter ATP-binding protein	Transport and binding proteins
SAK_0594	2	3.76	funct	2.92	funct	0.47	func	phosphoglucomutase/phosphomannomutase family protein	Energy metabolism
SAK_0595	2	1.31	funct	0.66	funct	0.9	func	methylenetetrahydrofolate dehydrogenase/methenyltetrahydrofolate cyclohydrolase	Biosynthesis of cofactors, prosthetic groups, and carriers
SAK_0596	2	1.63	funct	0.86	funct	0.87	func	hypothetical protein	Energy metabolism
SAK_0597	3	1.99	funct	1.15	funct	1.13	func	exodeoxyribonuclease VII large subunit	DNA metabolism
SAK_0598	1	1.84	funct	1.04	funct	1.33	func	exodeoxyribonuclease VII small subunit	DNA metabolism
SAK_0599	2	1.8	funct	1.02	funct	1.27	func	geranyltranstransferase	Biosynthesis of cofactors, prosthetic groups, and carriers
SAK_0600	3	1.43	funct	1.06	funct	1.65	func	hemolysin A	Cellular processes
SAK_0602	1	1.02	funct	1.03	funct	1.62	func	DNA repair protein RecN	DNA metabolism
SAK_0603	5	1.33	funct	1.2	funct	1.37	func	DegV family protein	Unknown function
SAK_0604	2	1.41	funct	1.44	funct	1.71	func	GDSL family lipase/acylhydrolase	Unknown function
SAK_0605	4	1.11	funct	1.69	funct	1.8	func	hypothetical protein	conserved hypothetical protein
SAK_0606	2	1.84	funct	0.75	funct	0.88	func	DNA-binding protein HU	DNA metabolism
SAK_0656	2	9.02	funct	1.19	funct	0.88	func	hypothetical protein	hypothetical protein
SAK_0657	2	1.22	funct	4.81	funct	0.45	func	dihydroorotate dehydrogenase 1A	Purines, pyrimidines, nucleosides, and nucleotides
SAK_0658	2	1.04	funct	0.55	pseudo	1.08	func	beta-lactam resistance factor	Cell envelope
SAK_0659	2	0.74	funct	0.59	funct	1.22	func	FemAB family protein	Cell envelope
SAK_0660	2	0.57	pseudo	0.67	funct	1.48	func	FemAB family protein	Cell envelope
SAK_0661	1	1.03	funct	0.82	funct	1.58	pseudo	Cof-like hydrolase	Unknown function
SAK_0662	8	1.81	funct	1.32	funct	1.58	func	HD domain-containing protein	Unknown function
SAK_0663	3	1.28	funct	6.15	funct	1.02	func	hypothetical protein	conserved hypothetical protein
SAK_0664	6	0.5	funct	0.72	funct	0.74	func	calcium-transporting ATPase	Transport and binding proteins
SAK_0667	3	1.06	funct	2.9	funct	0.68	func	iron-sulfur cluster-binding protein, putative	Energy metabolism
SAK_0668	2	1.26	funct	2.37	funct	1.5	func	peptide chain release factor 2	Protein synthesis
SAK_0669	3	1	funct	1.96	funct	1.18	func	cell division ATP binding protein FtsE	Cellular processes
SAK_0670	2	0.94	funct	1.87	funct	1.06	func	cell division protein FtsX, putative	Cellular processes
SAK_0672	1	0.86	funct	0.42	funct	0.83	func	hypothetical protein	conserved hypothetical protein
SAK_0673	2	1.05	funct	0.36	funct	0.8	func	metallo-beta-lactamase family protein	Unknown function
SAK_0674	3	1.76	funct	0.36	funct	0.32	func	acetoin reductase	Energy metabolism
SAK_0675	2	1.38	funct	0.99	funct	0.91	func	bifunctional ATP-dependent DNA helicase/DNA polymerase III subunit epsilon	DNA metabolism
SAK_0676	3	1.12	funct	0.83	funct	0.77	func	aspartate aminotransferase	Amino acid biosynthesis
SAK_0677	2	1.16	funct	0.84	funct	0.87	func	asparaginyl-tRNA synthetase	Protein synthesis
SAK_0678	1	0.79	funct	1.14	funct	0.84	func	hypothetical protein	conserved hypothetical protein
SAK_0679	3	0.6	funct	1.15	funct	0.81	func	inosine-uridine preferring nucleoside hydrolase family protein	Purines, pyrimidines, nucleosides, and nucleotides
SAK_0680	2	1.98	funct	0.78	funct	0.81	func	organic hydroperoxide resistance protein, putative	Cellular processes
SAK_0681	2	1.88	funct	1.2	funct	0.91	func	hypothetical protein	Unknown function
SAK_0682	2	2.01	funct	1.56	funct	1.09	func	hypothetical protein	conserved hypothetical protein
SAK_0683	3	1.65	funct	1.51	funct	1.25	func	hypothetical protein	conserved hypothetical protein
SAK_0684	3	1.54	funct	1.29	funct	1.21	func	dipeptidase A	Protein fate
SAK_0685	2	1.67	funct	2.27	funct	0.45	func	zinc ABC transporter, zinc-binding protein AdcA	Transport and binding proteins
SAK_0686	3	0.93	funct	1.03	funct	0.81	func	50S ribosomal protein L31 type B	Protein synthesis
SAK_0687	2	1.1	funct	0.71	funct	0.88	func	DHH family protein	Unknown function
SAK_0688	2	0.81	funct	0.61	funct	0.88	func	adenosine deaminase	Purines, pyrimidines, nucleosides, and nucleotides
SAK_0689	1	0.97	funct	1.34	funct	1.17	func	flavodoxin	Energy metabolism
SAK_0690	1	0.8	funct	0.83	funct	1.59	func	hypothetical protein	Amino acid biosynthesis
SAK_0691	2	0.65	funct	1.38	pseudo	1.4	func	voltage-gated chloride channel family protein	Transport and binding proteins
SAK_0692	3	0.49	funct	1.02	funct	0.86	func	50S ribosomal protein L19	Protein synthesis
SAK_0706	2	1.02	funct	1.57	funct	1.14	func	cell cycle protein FtsW	Cellular processes
SAK_0707	5	1.48	funct	1.93	funct	1.2	func	HAD-superfamily hydrolase, subfamily IA, variant 1 family protein	Unknown function
SAK_0708	3	0.83	funct	1.88	funct	1.42	func	DNA gyrase subunit B	DNA metabolism
SAK_0709	2	0.89	funct	1.95	funct	1.02	func	septation ring formation regulator EzrA	Cellular processes
SAK_0710	3	1.36	funct	1	funct	0.92	func	phosphoserine phosphatase SerB	Amino acid biosynthesis
SAK_0711	1	2.12	funct	0.64	funct	0.64	func	NUDIX family hydrolase	Unknown function
SAK_0712	1	2.08	funct	0.61	funct	0.91	func	hypothetical protein	conserved hypothetical protein
SAK_0713	3	1.35	funct	1.2	funct	0.89	func	phosphopyruvate hydratase	Energy metabolism
SAK_0714	1	0.8	pseudo	0.66	funct	0.93	func	hypothetical protein	conserved domain protein
SAK_0715	2	2.27	funct	0.67	funct	0.97	func	3-phosphoshikimate 1-carboxyvinyltransferase	Amino acid biosynthesis
SAK_0716	2	1.78	funct	0.7	funct	1	func	shikimate kinase	Amino acid biosynthesis
SAK_0717	1	1.92	funct	0.77	funct	0.87	func	transcriptional regulator, putative	Regulatory functions

SAK_0718	2	0.94	funct	1.3	funct	0.97	func	23S rRNA (uracil-5-)-methyltransferase RumA	Protein synthesis
SAK_0786	1	0.7	funct	0.49	funct	1.51	func	beta-lactamase, putative	Cellular processes
SAK_0787	3	0.5	funct	0.77	funct	0.83	func	ABC transporter, ATP-binding protein	Transport and binding proteins
SAK_0813	3	2.32	funct	1.37	funct	1.27	func	hypothetical protein	conserved hypothetical protein
SAK_0814	2	0.69	funct	3.12	funct	2.56	func	DNA-entry nuclease, putative	Cellular processes
SAK_0815	1	1.65	funct	0.58	funct	0.41	func	DedA family protein	Unknown function
SAK_0816	1	0.65	funct	0.66	funct	0.23	func	ABC transporter, ATP-binding protein	Transport and binding proteins
SAK_0817	2	0.39	funct	0.4	funct	0.23	func	hypothetical protein	Cell envelope
SAK_0818	2	1.2	funct	0.6	funct	0.84	func	hypothetical protein	conserved hypothetical protein
SAK_0819	1	1.34	funct	0.11	pseudo	0.87	func	LysR family transcriptional regulator	Regulatory functions
SAK_0820	2	3.39	funct	0.79	pseudo	0.89	func	hypothetical protein	Cell envelope
SAK_0821	2	3.2	funct	0.67	funct	0.85	func	D-lactate dehydrogenase	Energy metabolism
SAK_0824	1	1.3	funct	0.36	pseudo	1.06	func	beta-D-glucuronidase	Energy metabolism
SAK_0825	1	1.12	pseudo	0.49	funct	0.86	func	GntR family transcriptional regulator	Regulatory functions
SAK_0826	2	7.83	pseudo	4.77	pseudo	0.35	func	2-dehydro-3-deoxyphosphogluconate aldolase/4-hydroxy-2-oxoglutarate aldolase	Amino acid biosynthesis
SAK_0827	3	6.06	pseudo	4.6	funct	0.37	func	glucuronate isomerase	Energy metabolism
SAK_0828	2	5.02	funct	3.94	funct	0.4	func	mannonate dehydratase	Energy metabolism
SAK_0829	2	7.57	funct	4.42	funct	0.41	func	D-mannonate oxidoreductase	Energy metabolism
SAK_0831	4	4.76	pseudo	1.93	pseudo	0.63	func	glycosy hydrolase family protein	Energy metabolism
SAK_0832	2	1.22	funct	0.52	funct	0.85	func	Xaa-Pro dipeptidase	Protein fate
SAK_0833	9	1.63	funct	0.95	funct	0.42	func	catabolite control protein A	Regulatory functions
SAK_0834	3	1.8	pseudo	1.36	pseudo	0.94	func	cytoplasmic alpha-amylase	Energy metabolism
SAK_0835	2	0.97	funct	1.09	funct	1.15	func	glycosyl transferase, group 1 family protein	Cell envelope
SAK_0836	1	0.85	funct	1.1	funct	1.25	func	glycosyl transferase, group 1 family protein	Cell envelope
SAK_0837	2	0.54	funct	0.88	funct	0.9	func	threonyl-tRNA synthetase	Protein synthesis
SAK_0839	2	1.5	funct	1.31	funct	0.92	func	hypothetical protein	conserved hypothetical protein
SAK_0840	1	1.23	funct	6.05	pseudo	2.04	func	peptidase propeptide/YPEB domain-containing protein	Unknown function
SAK_0842	3	0.5	funct	1.73	funct	0.51	func	polar amino acid ABC transporter permease	Transport and binding proteins
SAK_0843	3	0.66	pseudo	2.05	funct	0.64	pseudo	polar amino acid ABC transporter amino acid-binding protein	Transport and binding proteins
SAK_0844	2	1.05	funct	2.41	funct	0.63	func	polar amino acid ABC transporter ATP-binding protein	Transport and binding proteins
SAK_0845	2	1.86	funct	1.47	funct	1.38	func	DNA-binding response regulator	Signal transduction
SAK_0846	2	1.48	funct	0.91	funct	1.44	func	sensory box histidine kinase	Signal transduction
SAK_0847	2	1.57	funct	0.62	funct	1.25	func	metallo-beta-lactamase family protein	Unknown function
SAK_0848	2	1.64	funct	0.45	funct	1.15	func	hypothetical protein	conserved hypothetical protein
SAK_0849	3	1.21	funct	1.29	funct	0.83	func	ribonuclease III	Transcription
SAK_0850	3	1.05	funct	1.92	funct	1.22	func	chromosome segregation protein SMC	Cellular processes
SAK_0852	2	1.63	funct	1.43	pseudo	1.16	func	Cof-like hydrolase	Unknown function
SAK_0853	2	1.55	funct	0.91	funct	0.99	func	signal recognition particle-docking protein FtsY	Protein fate
SAK_0854	3	0.96	funct	0.37	funct	1.32	func	hypothetical protein	conserved hypothetical protein
SAK_0855	2	1.14	funct	0.5	funct	1.25	func	permease, putative	Transport and binding proteins
SAK_0856	2	1.6	funct	0.66	funct	1.39	func	hypothetical protein	hypothetical protein
SAK_0857	1	9.98	funct	20.93	funct	1.14	func	luciferase family protein	Unknown function
SAK_0858	2	0.58	funct	0.63	funct	0.81	func	S1 RNA-binding domain-containing protein	Unknown function
SAK_0859	3	0.84	funct	0.83	funct	0.69	func	hypothetical protein	Unknown function
SAK_0860	2	2.88	funct	1.77	funct	1.17	func	PspC domain-containing protein	Unknown function
SAK_0862	2	3.01	funct	1.28	funct	0.95	func	HPr kinase/phosphorylase	Regulatory functions
SAK_0863	1	2.2	funct	1.35	funct	1.34	func	prolipoprotein diacylglyceryl transferase	Protein fate
SAK_0864	2	1.62	funct	1.36	funct	1.26	func	hypothetical protein	conserved hypothetical protein
SAK_0865	2	1.88	funct	1.39	funct	1.44	func	hypothetical protein	conserved hypothetical protein
SAK_0866	2	0.76	funct	1.96	funct	0.7	func	hypothetical protein	conserved hypothetical protein
SAK_0867	2	1.24	funct	0.91	funct	0.96	func	U32 family peptidase	Protein fate
SAK_0868	3	0.87	funct	0.79	funct	0.79	func	U32 family peptidase	Protein fate
SAK_0869	4	1.01	funct	0.97	funct	1.21	func	hypothetical protein	conserved hypothetical protein
SAK_0870	2	7.62	funct	0.9	funct	0.39	func	hypothetical protein	Cell envelope
SAK_0871	1	0.89	funct	0.66	funct	0.39	func	Mn2+/Fe2+ transporter	Transport and binding proteins
SAK_0872	1	0.8	funct	1.42	funct	1.41	func	riboflavin biosynthesis protein RibD	Biosynthesis of cofactors, prosthetic groups, and carriers
SAK_0873	2	1.39	funct	1.96	funct	1.84	func	riboflavin synthase subunit alpha	Biosynthesis of cofactors, prosthetic groups, and carriers
SAK_0874	2	1.61	funct	3.19	funct	2.88	func	3,4-dihydroxy-2-butanone-4-phosphate synthase/GTP cyclohydrolase II	Biosynthesis of cofactors, prosthetic groups, and carriers
SAK_0875	2	1.54	funct	3.11	funct	2.66	func	6,7-dimethyl-8-ribityllumazine synthase	Biosynthesis of cofactors, prosthetic groups, and carriers
SAK_0877	1	0.76	funct	1.7	funct	0.98	func	HAD-superfamily hydrolase, subfamily IA, variant 1 family protein	Unknown function
SAK_0878	3	1.14	funct	1.65	pseudo	0.94	func	phosphoglycerate mutase family protein	Unknown function
SAK_0879	3	0.79	funct	1.2	funct	0.96	func	YbaK/EbsC family protein	Unknown function
SAK_0880	1	0.95	funct	0.75	funct	0.71	func	hypothetical protein	conserved domain protein
SAK_0881	4	1.45	funct	1.83	funct	0.53	func	glycosy hydrolase family protein	Cell envelope
SAK_0882	3	0.73	funct	1.05	funct	0.99	func	hypothetical protein	conserved hypothetical protein
SAK_0883	1	2.15	0	1.18	funct	0.9	func	putative lipoprotein	Cell envelope
SAK_0884	1	0.64	pseudo	0.37	pseudo	0.76	func	oligoendopeptidase F	Protein fate
SAK_0885	3	0.55	funct	2.02	funct	1.3	func	phosphoenolpyruvate carboxylase	Energy metabolism
SAK_0886	7	0.63	funct	1.41	funct	1	pseudo ?	cell cycle protein FtsW	Cellular processes
SAK_0887	1	0.76	funct	0.45	funct	1.04	func	elongation factor Tu	Protein synthesis
SAK_0888	2	0.69	funct	0.34	funct	0.96	func	triosephosphate isomerase	Energy metabolism
SAK_0889	4	0.77	funct	0.47	funct	1.01	func	phosphoglyceromutase	Energy metabolism
SAK_0890	2	0.99	funct	0.59	funct	1.16	func	penicillin-binding protein 2b	Cell envelope
SAK_0891	3	1.07	funct	0.62	funct	1.04	func	recombination protein RecR	DNA metabolism
SAK_0892	3	0.71	funct	1.67	funct	0.85	func	D-alanyl-alanine synthetase A	Cell envelope
SAK_0893	3	1.66	funct	1.91	funct	1.25	func	UDP-N-acetylmuramoyl-tripeptide--D-alanyl-D-alanine ligase	Cell envelope
SAK_0894	3	12.56	funct	15.17	funct	0.5	func	major facilitator family transporter	Transport and binding proteins
SAK_0895	3	3.25	funct	6.08	funct	1.58	func	hypothetical protein	Cell envelope

SAK_0896	4	5.58	funct	9.53	funct	1.97	func	cell wall surface anchor family protein	Cell envelope
SAK_0897	2	0.73	funct	1.08	funct	0.87	func	peptide chain release factor 3	Protein synthesis
SAK_0899	1	0.46	funct	0.34	pseudo	0.66	func	amino acid ABC transporter, ATP-binding protein, putative	Transport and binding proteins
SAK_0900	1	0.4	funct	0.41	funct	0.89	func	amino acid ABC transporter, permease protein, putative	Transport and binding proteins
SAK_0901	1	0.46	funct	0.4	funct	0.97	func	ABC transporter, substrate-binding protein	Transport and binding proteins
SAK_0902	3	0.37	funct	0.37	funct	0.99	func	DEAD-box ATP dependent DNA helicase	Transcription
SAK_0903	4	1.24	funct	1.5	funct	0.96	func	GIY-YIG domain-containing protein	Unknown function
SAK_0904	3	1.42	funct	1.29	funct	0.96	func	hypothetical protein	conserved hypothetical protein
SAK_0905	2	1.48	funct	1	funct	0.83	func	acyltransferase family protein	Unknown function
SAK_0908	2	1.32	pseudo	0.6	pseudo	0.97	func	Cof-like hydrolase family protein	Unknown function
SAK_0909	1	1.07	pseudo	0.74	funct	1.01	func	LacI family sugar-binding transcriptional regulator	Regulatory functions
SAK_0910	2	0.61	funct	0.19	funct	1.12	func	hypothetical protein	Unknown function
SAK_0911	2	2.13	funct	1	funct	1.2	func	hypothetical protein	conserved hypothetical protein
SAK_0912	2	1.61	funct	0.95	funct	1.06	func	DNA polymerase III subunit delta	DNA metabolism
SAK_0913	2	1.72	funct	0.57	funct	0.96	func	superoxide dismutase, Mn	Cellular processes
SAK_0916	2	1.26	funct	1.3	funct	0.86	func	glycosy hydrolase family protein	Energy metabolism
SAK_0917	2	0.87	funct	0.95	funct	1.17	func	hypothetical protein	conserved hypothetical protein
SAK_0918	1	0.29	funct	1.28	funct	0.39	func	glycerate kinase	Energy metabolism
SAK_0919	1	0.42	funct	1.2	funct	0.32	func	GntP family permease	Transport and binding proteins
SAK_0920	2	0.52	pseudo	1.29	pseudo	0.47	func	hypothetical protein	conserved hypothetical protein
SAK_0922	2	1.06	funct	0.58	funct	0.85	func	S-adenosylmethionine:tRNA ribosyltransferase-isomerase	Protein synthesis
SAK_0923	3	0.81	funct	0.15	funct?	0.27	func	hypothetical protein	Cell envelope
SAK_0924	5	0.3	funct	1	funct	1.2	func	glucosamine-6-phosphate isomerase	Central intermediary metabolism
SAK_0925	3	16.85	funct	14.75	funct	0.93	func	glutathione S-transferase domain-containing protein	Unknown function
SAK_0926	1	1.05	funct	1.49	funct	0.92	func	RNA pseudouridine synthase family protein	Protein synthesis
SAK_0927	5	0.52	funct	0.73	funct	0.69	func	major facilitator family protein	Transport and binding proteins
SAK_0929	1	0.63	funct	0.59	funct	0.91	func	group B oligopeptidase PepB	Protein fate
SAK_0931	1	0.41	funct	0.68	funct	1.88	func	O-methyltransferase family protein	Unknown function
SAK_0932	7	1.06	funct	1.87	funct	2.45	func	foldase protein PrsA	Protein fate
SAK_0933	4	0.63	funct	1.56	funct	0.98	func	hypothetical protein	conserved hypothetical protein
SAK_0934	5	0.82	funct	1.41	funct	0.78	func	alanyl-tRNA synthetase	Protein synthesis
SAK_0935	2	0.75	pseudo	1.75	funct	1.01	func	hypothetical protein	Cell envelope
SAK_0939	1	2.38	funct	3.09	pseudo	0.92	func	hypothetical protein	Cell envelope
SAK_0940	2	0.37	pseudo	0.34	pseudo	0.67	func	hypothetical protein	Transport and binding proteins
SAK_0942	1	2.03	funct	0.94	funct	1.93	func	ribonucleotide-diphosphate reductase subunit beta	Purines, pyrimidines, nucleosides, and nucleotides
SAK_0943	2	1.6	funct	1.02	funct	2.02	func	ribonucleotide-diphosphate reductase subunit alpha	Purines, pyrimidines, nucleosides, and nucleotides
SAK_0944	2	1.46	funct	0.62	funct	1.07	func	glutaredoxin-like protein NrdH	Energy metabolism
SAK_0945	3	1.17	funct	1.24	funct	0.88	func	phosphocarrier protein HPr	Signal transduction
SAK_0946	3	1.02	funct	1.14	funct	0.91	func	phosphoenolpyruvate-protein phosphotransferase	Signal transduction
SAK_0947	2	1.7	funct	1.3	funct	1.03	func	glyceraldehyde-3-phosphate dehydrogenase, NADP-dependent	Energy metabolism
SAK_0948	1	5.6	funct	16.38	funct	1.43	func	polysaccharide deacetylase family protein	Energy metabolism
SAK_0949	2	1.82	funct	0.81	funct	1.01	func	DEAD-box ATP dependent DNA helicase	Transcription
SAK_0950	2	1.51	funct	0.46	pseudo	1.16	func	uridine kinase	Purines, pyrimidines, nucleosides, and nucleotides
SAK_0951	1	1.26	funct	0.95	funct	1.06	func	GAF domain-containing protein	Unknown function
SAK_0952	2	0.97	funct	1	funct	1.12	func	DNA polymerase III subunits gamma and tau	DNA metabolism
SAK_0953	4	1.97	funct	0.39	funct	0.95	func	biotin--protein ligase	Protein fate
SAK_0954	2	1.2	funct	0.78	funct	0.75	func	S-adenosylmethionine synthetase	Central intermediary metabolism
SAK_0956	1	0.84	funct	1.01	funct	2.49	func	hypothetical protein	conserved hypothetical protein
SAK_0966	1	0.33	funct	1.37	funct	0.57	func	UDP-N-acetylglucosamine 1-carboxyvinyltransferase	Cell envelope
SAK_0967	2	1.7	pseudo	1.38	funct	1.5	func	acetyltransferase	Unknown function
SAK_0968	2	1.09	funct	1.41	funct	1.58	func	DRTGG domain/CBS domain-containing protein	Unknown function
SAK_0969	2	1.23	funct	1.15	funct	1.3	func	methionine aminopeptidase	Protein fate
SAK_0970	3	1.23	funct	1.19	funct	1.16	func	ribonuclease BN, putative	Unknown function
SAK_0971	2	1.83	funct	1.09	pseudo	0.8	func	GtrA family protein	Unknown function
SAK_0972	5	0.39	funct	0.96	funct	0.64	func	hypothetical protein	conserved hypothetical protein
SAK_0973	2	1.4	funct	0.9	funct	0.95	func	NAD-dependent DNA ligase LigA	DNA metabolism
SAK_0974	2	1.28	funct	0.81	funct	1.01	func	putative lipid kinase	conserved hypothetical protein TIGR00147
SAK_0975	4	1.33	funct	0.84	funct	1.07	func	pullulanase, type I	Energy metabolism
SAK_0977	4	0.4	funct	0.65	funct	0.4	func	glucose-1-phosphate adenylyltransferase	Energy metabolism
SAK_0978	3	0.43	pseudo	0.68	pseudo	0.41	func	glucose-1-phosphate adenylyltransferase, GlgD subunit	Energy metabolism
SAK_0979	2	0.43	funct	0.56	pseudo	0.44	func	glycogen synthase	Energy metabolism
SAK_0980	3	0.51	funct	0.23	funct	1.38	func	F0F1 ATP synthase subunit C	Energy metabolism
SAK_0981	3	0.52	funct	0.26	funct	1.38	func	F0F1 ATP synthase subunit A	Energy metabolism
SAK_0982	3	0.51	funct	0.26	funct	1.3	func	F0F1 ATP synthase subunit B	Energy metabolism
SAK_0983	3	0.44	funct	0.29	funct	1.22	func	F0F1 ATP synthase subunit delta	Energy metabolism
SAK_0984	3	0.61	funct	0.36	funct	1.44	func	F0F1 ATP synthase subunit alpha	Energy metabolism
SAK_0985	1	0.54	funct	0.35	funct	1.51	func	F0F1 ATP synthase subunit gamma	Energy metabolism
SAK_0986	2	0.67	funct	0.44	funct	1.4	func	F0F1 ATP synthase subunit beta	Energy metabolism
SAK_0987	2	0.68	funct	0.48	funct	1.22	func	F0F1 ATP synthase subunit epsilon	Energy metabolism
SAK_0988	2	0.8	funct	1.1	funct	0.84	func	hypothetical protein	conserved hypothetical protein TIGR02327
SAK_0989	1	0.75	funct	1.08	funct	1.02	func	UDP-N-acetylglucosamine 1-carboxyvinyltransferase	Cell envelope
SAK_0990	2	0.41	funct	1.09	funct	0.91	func	hypothetical protein	conserved hypothetical protein

SAK_0991	3	1.74	funct	1.5	funct	1.23	func	DNA-entry nuclease	Cellular processes
SAK_0992	2	0.3	funct	0.55	funct	1.19	func	phenylalanyl-tRNA synthetase subunit alpha	Protein synthesis
SAK_0993	5	0.31	pseudo	0.58	funct	1.11	func	acetyltransferase	Unknown function
SAK_0994	4	0.25	funct	0.45	funct	1.18	func	phenylalanyl-tRNA synthetase subunit beta	Protein synthesis
SAK_0995	5	3.12	funct	0.38	funct	0.27	func	hypothetical protein	conserved hypothetical protein
SAK_0996	1	1.66	funct	2.18	funct	1.63	func	exonuclease RexB	DNA metabolism
SAK_0997	1	1.86	funct	2.24	funct	1.97	pseudo	exonuclease RexA	DNA metabolism
SAK_0998	3	2.04	pseudo	2.01	funct	1.48	func	CorA family metal ion transporter	Transport and binding proteins
SAK_0999	3	0.68	funct	0.86	funct	1	func	tRNA modification GTPase TrmE	Protein synthesis
SAK_1000	1	2.3	funct	1.19	funct	1.55	func	ABC transporter, ATP-binding protein	Transport and binding proteins
SAK_1001	3	1.04	funct	0.47	funct	0.68	func	acetoin dehydrogenase, TPP-dependent, E1 component, alpha subunit, putative	Energy metabolism
SAK_1002	2	0.81	funct	0.75	funct	0.77	func	acetoin dehydrogenase, TPP-dependent, E1 component, beta subunit, putative	Energy metabolism
SAK_1003	1	0.63	funct	0.85	funct;17 AA ds ttes pseudorf unctt	1.14	func	branched-chain alpha-keto acid dehydrogenase subunit E2	Energy metabolism
SAK_1004	3	0.76	funct	0.99	funct;17 AA ds ttes pseudorf unctt	1.5	func	acetoin dehydrogenase, TPP-dependent, E3 component, dihydrolipoamide dehydrogenase, putative	Energy metabolism
SAK_1005	2	1.25	funct	2.24	pseudo	1.99	func	lipoate-protein ligase A	Protein fate
SAK_1006	3	1.55	funct	0.84	funct	1.08	func	glutamine amidotransferase domain-containing protein	Unknown function
SAK_1007	3	1.2	funct	0.73	funct	1	func	mur ligase family protein	Cell envelope
SAK_1008	3	2.12	funct	0.94	funct	0.97	func	hypothetical protein	conserved hypothetical protein TIGR00159
SAK_1009	1	1.56	funct	1.26	funct	1.27	func	hypothetical protein	conserved hypothetical protein
SAK_1010	2	2.17	funct	1.4	funct	1.2	func	phosphoglucosamine mutase	Cell envelope
SAK_1011	2	1.57	funct	1.31	funct	2.21	func	hypothetical protein	conserved hypothetical protein
SAK_1012	2	1.63	funct	1.03	funct	2	func	hypothetical protein	conserved hypothetical protein
SAK_1013	6	0.94	funct	0.65	funct	1.03	func	coproporphyrinogen III oxidase	Biosynthesis of cofactors, prosthetic groups, and carriers
SAK_1014	5	0.97	funct	0.62	funct	1.08	func	thioesterase	Fatty acid and phospholipid metabolism
SAK_1015	3	1.09	funct	0.65	funct	1.14	func	HAD family hydrolase	Unknown function
SAK_1016	2	1.27	funct	0.75	funct	1.02	func	hypothetical protein	Cell envelope
SAK_1017	1	0.72	funct	0.41	funct	1.31	func	hypothetical protein	Mobile and extrachromosomal element functions
SAK_1019	1	0.47	funct	0.49	funct	1.22	func	CRISPR-associated Cas2 family protein	Mobile and extrachromosomal element functions
SAK_1021	3	4.27	funct	2.26	funct	1.23	func	nucleoside diphosphate kinase	Purines, pyrimidines, nucleosides, and nucleotides
SAK_1022	3	2.55	funct	1.46	funct	0.99	func	GTP-binding protein LepA	Unknown function
SAK_1024	2	1.47	funct	0.76	funct	1.53	func	HD domain-containing protein	Unknown function
SAK_1025	1	1.49	funct	0.79	pseudo	1.24	func	acetyltransferase	Unknown function
SAK_1026	3	1.65	funct	0.64	funct	1.14	func	methionine sulfoxide reductase B	Protein fate
SAK_1027	2	2.59	pseudo	0.74	funct	0.94	func	cation transporter HAD ATPase	Transport and binding proteins
SAK_1028	2	1.75	pseudo	0.86	funct	1.04	func	hypothetical protein	conserved hypothetical protein
SAK_1029	3	1.13	funct	1.05	pseudo	0.75	func	hexapaptide repeat-containing transferase	Unknown function
SAK_1031	4	2.06	funct	1.62	funct	0.73	func	hypothetical protein	Cell envelope
SAK_1032	1	1.01	funct	2.66	funct	1.35	func	hypothetical protein	Cell envelope
SAK_1033	3	1	funct	2.5	funct	1.87	func	ABC transporter, ATP-binding protein	Transport and binding proteins
SAK_1034	4	0.95	funct	2.25	funct	1.84	func	GntR family transcriptional regulator	Regulatory functions
SAK_1035	2	1.73	funct	0.84	funct	1.04	func	DNA polymerase III DnaE	DNA metabolism
SAK_1036	6	1.19	funct	0.57	funct	0.95	func	6-phosphofructokinase	Energy metabolism
SAK_1037	2	1.12	funct	0.57	funct	1.13	func	pyruvate kinase	Energy metabolism
SAK_1038	1	3.71	funct	4.4	funct	0.91	func	signal peptidase I	Protein fate
SAK_1040	4	0.8	funct	0.57	funct	1.34	func	glucosamine--fructose-6-phosphate aminotransferase	Central intermediary metabolism
SAK_1041	1	1.12	funct	1.48	funct	0.63	func	PhnA protein	Unknown function
SAK_1042	3	0.61	funct	0.58	funct	1.25	func	polar amino acid ABC transporter permease	Transport and binding proteins
SAK_1043	3	0.4	funct	0.56	funct	1.34	func	polar amino acid ABC transporter ATP-binding protein	Transport and binding proteins
SAK_1044	2	0.32	funct	0.59	funct	1.33	func	polar amino acid ABC transporter amino acid-binding protein	Transport and binding proteins
SAK_1045	4	0.78	funct	1.34	funct	1.45	func	30S ribosomal protein S20	Protein synthesis
SAK_1046	3	0.93	funct	1.73	funct	1.52	func	pantothenate kinase	Biosynthesis of cofactors, prosthetic groups, and carriers
SAK_1047	3	1.28	funct	1.51	funct	0.78	func	methyltransferase domain-containing protein	Unknown function
SAK_1048	1	1.55	0	1.98	0	0.72	func	cytidine deaminase	Purines, pyrimidines, nucleosides, and nucleotides
SAK_1050	3	0.61	pseudo	0.91	pseudo	0.63	func	carbohydrate ABC transporter periplasmic-binding protein	Transport and binding proteins
SAK_1051	3	0.5	funct	0.74	pseudo	0.56	func	carbohydrate ABC transporter permease	Transport and binding proteins
SAK_1053	5	0.25	funct	0.86	funct	2.41	func	NADH oxidase, water-forming	Energy metabolism
SAK_1054	4	2.21	funct	0.86	funct	0.67	func	L-lactate dehydrogenase	Energy metabolism
SAK_1055	1	0.92	funct	1.73	funct	1.14	func	DNA gyrase subunit A	DNA metabolism
SAK_1056	3	0.68	funct	1.38	funct	1.2	func	sortase family protein	Cell envelope
SAK_1057	3	0.79	funct	1.58	funct	1.27	func	glyoxylase family protein	Unknown function
SAK_1058	6	1.28	funct	0.86	funct	1.35	func	hypothetical protein	conserved hypothetical protein
SAK_1059	2	1.7	pseudo	0.58	pseudo	0.61	func	Na+/H+ antiporter	Transport and binding proteins
SAK_1062	2	1.39	funct	0.76	funct	0.87	func	GMP synthase	Purines, pyrimidines, nucleosides, and nucleotides
SAK_1063	2	1.96	funct	0.49	funct	0.83	func	GntR family transcriptional regulator	Regulatory functions
SAK_1064	3	1.27	funct	0.9	funct	1.34	func	tRNA (uracil-5-)-methyltransferase Gid	Unknown function
SAK_1065	3	1.08	funct	1.21	funct	2.62	func	acetyltransferase	Unknown function
SAK_1066	2	0.88	pseudo	7.39	funct	1.92	func	NPLA family lipoprotein	Cell envelope

SAK_1067	4	2	pseudo	0.19	pseudo	0.98	func	hypothetical protein	conserved hypothetical protein
SAK_1068	1	1.17	funct	2.47	funct	0.99	pseudo	nisin resistance protein Nsr, putative	Cellular processes
SAK_1069	2	1.44	funct	0.82	funct	0.96	func	ABC transporter, ATP-binding protein	Transport and binding proteins
SAK_1070	3	1.24	funct	0.87	funct	0.98	func	ABC transporter, permease protein, putative	Transport and binding proteins
SAK_1071	1	3.64	funct	0.67	pseudo	0.13	func	DNA-binding response regulator	Signal transduction
SAK_1072	1	3.71	funct	0.79	funct	0.58	pseudo	sensor histidine kinase	Signal transduction
SAK_1073	1	1.84	funct	5.52	pseudo	1.64	func	site-specific tyrosine recombinase XerS	DNA metabolism
SAK_1074	1	1.8	pseudo	0.71	pseudo	0.57	func	ABC transporter, substrate-binding protein	Transport and binding proteins
SAK_1075	3	2.08	funct	1.03	funct	0.72	func	hypothetical protein	Cell envelope
SAK_1076	4	1.67	funct	0.75	funct	0.72	func	satD protein	Unknown function
SAK_1077	6	1.97	funct	0.75	funct	1.11	func	signal recognition particle protein	Protein fate
SAK_1078	5	1.84	funct	1.08	funct	1.16	func	putative DNA-binding protein	Unknown function
SAK_1079	3	1.1	funct	2.16	funct	1.83	func	sensor histidine kinase CiaH	Signal transduction
SAK_1080	3	2.64	funct	2.77	funct	2.12	func	DNA-binding response regulator CiaR	Signal transduction
SAK_1081	3	0.72	funct	0.66	funct	1.23	func	aminopeptidase N	Protein fate
SAK_1082	1	0.46	funct	0.7	funct	1.77	func	phosphate transport system regulatory protein PhoU	Regulatory functions
SAK_1083	2	0.49	funct	0.78	funct	1.83	func	phosphate ABC transporter, ATP-binding protein PstB, putative	Transport and binding proteins
SAK_1084	3	0.7	funct	0.67	funct	1.53	func	phosphate ABC transporter, ATP-binding protein PstB	Transport and binding proteins
SAK_1085	3	0.75	funct	0.65	funct	1.33	func	phosphate ABC transporter, permease protein PtsA	Transport and binding proteins
SAK_1086	2	0.86	funct	0.6	funct	1.22	func	phosphate ABC transporter, permease protein PstC	Transport and binding proteins
SAK_1087	1	0.83	funct	0.61	funct	1.33	func	phosphate ABC transporter, phosphate-binding protein	Transport and binding proteins
SAK_1088	3	1.75	pseudo	1.23	pseudo	1.4	func	NOL1/NOP2/sun family putative RNA methylase	Protein synthesis
SAK_1089	3	0.38	funct	0.79	funct	1.23	func	inositol monophosphatase family protein	Unknown function
SAK_1090	2	0.5	funct	0.84	funct	1.28	func	hypothetical protein	conserved hypothetical protein
SAK_1091	2	0.75	funct	0.94	funct	1.28	func	transcriptional regulator Spx	Regulatory functions
SAK_1092	2	0.52	funct	0.89	funct	1.12	func	bifunctional riboflavin kinase/FMN adenylyltransferase	Biosynthesis of cofactors, prosthetic groups, and carriers
SAK_1093	3	0.65	funct	0.84	funct	1.17	func	tRNA pseudouridine synthase B	Protein synthesis
SAK_1094	2	0.99	funct	1.31	funct	1.82	func	acetyltransferase	Unknown function
SAK_1095	1	0.89	funct	1.62	pseudo	1.99	func	hypothetical protein	conserved hypothetical protein
SAK_1096	4	1.02	funct	1.43	funct	1.96	func	hypothetical protein	conserved hypothetical protein
SAK_1097	2	1.28	funct	2.45	funct	1.33	func	CAAX amino terminal protease family protein	Unknown function
SAK_1098	2	1.29	funct	1.59	funct	1.62	func	ABC transporter, permease protein, putative	Transport and binding proteins
SAK_1100	3	0.63	funct	0.71	funct	0.88	func	DNA topoisomerase I	DNA metabolism
SAK_1102	2	0.91	funct	3.39	funct	1.54	func	iron chelate uptake ABC transporter, iron chelate-binding protein	Transport and binding proteins
SAK_1103	3	0.78	funct	2.73	funct	1.8	func	iron chelate uptake ABC transporter, ATP-binding protein	Transport and binding proteins
SAK_1104	1	0.75	funct	2.79	funct	1.72	pseudo	iron chelate uptake ABC transporter, permease protein	Transport and binding proteins
SAK_1105	1	1.05	funct	2.36	funct	1.45	func	iron chelate uptake ABC transporter, permease protein	Transport and binding proteins
SAK_1106	2	1	funct	1.27	funct	1.26	func	maltose O-acetyltransferase	Energy metabolism
SAK_1107	2	1.1	funct	1.21	funct	1.37	func	ribonuclease HII	Transcription
SAK_1108	2	1.4	funct	1.02	funct	1.17	func	ribosomal biogenesis GTPase	Unknown function
SAK_1109	2	0.58	funct	0.65	funct	1.73	func	hypothetical protein	Unknown function
SAK_1110	1	19.95	funct	6.62	funct	1.05	func	carbon starvation protein CstA	Cellular processes
SAK_1111	2	1.39	funct	2.9	funct	1.39	func	DNA-binding response regulator	Signal transduction
SAK_1112	3	1.59	funct	3.04	funct	1.33	func	sensor histidine kinase	Signal transduction
SAK_1128	1	0.26	pseudo	1.51	pseudo	0.57	func	hypothetical protein	conserved hypothetical protein
SAK_1129	1	0.65	funct	1.04	0	0.75	func	hypothetical protein	conserved hypothetical protein
SAK_1130	1	1.08	funct	0.5	funct	0.85	func	hypothetical protein	conserved domain protein
SAK_1132	4	0.94	funct	0.43	funct	0.73	func	carbamoyl phosphate synthase large subunit	Purines, pyrimidines, nucleosides, and nucleotides
SAK_1133	2	0.72	funct	0.5	funct	0.57	func	carbamoyl phosphate synthase small subunit	Purines, pyrimidines, nucleosides, and nucleotides
SAK_1134	2	0.61	funct	0.76	funct	0.65	func	aspartate carbamoyltransferase catalytic subunit	Purines, pyrimidines, nucleosides, and nucleotides
SAK_1135	4	0.3	funct	0.68	funct	0.64	func	dihydroorotase	Purines, pyrimidines, nucleosides, and nucleotides
SAK_1136	2	0.37	funct	0.69	funct	0.73	func	orotate phosphoribosyltransferase	Purines, pyrimidines, nucleosides, and nucleotides
SAK_1137	1	0.39	funct	0.71	funct	0.74	func	orotidine 5'-phosphate decarboxylase	Purines, pyrimidines, nucleosides, and nucleotides
SAK_1138	2	0.48	pseudo	1.12	pseudo	1.17	func	hypothetical protein	Cell envelope
SAK_1139	6	1.04	pseudo	1.32	funct	1.27	func	ABC transporter, ATP-binding protein	Transport and binding proteins
SAK_1140	2	1.51	funct	0.62	funct	0.67	func	ATP cone domain-containing protein	Unknown function
SAK_1143	2	0.64	funct	0.94	funct	1.25	func	cardiolipin synthase, putative	Fatty acid and phospholipid metabolism
SAK_1144	1	0.82	funct	1.4	funct	1.06	func	formate--tetrahydrofolate ligase	Central intermediary metabolism
SAK_1145	2	0.62	funct	0.62	pseudo	1.35	func	lipoate-protein ligase A	Protein fate
SAK_1146	3	0.6	funct	0.68	funct	1.39	func	hypothetical protein	conserved hypothetical protein
SAK_1147	2	0.61	funct	0.54	funct	1.33	func	hypothetical protein	Unknown function
SAK_1148	1	0.6	funct	0.46	funct	1.09	func	GcvH family protein	Unknown function
SAK_1149	1	0.49	funct	0.45	funct	1.11	func	luciferase family protein	Unknown function
SAK_1150	2	0.51	funct	0.46	pseudo	0.82	func	oxidoreductase, FAD/FMN-binding	Unknown function
SAK_1151	2	0.71	funct	0.34	funct	0.71	func	hypothetical protein	conserved hypothetical protein
SAK_1152	1	0.62	funct	0.53	funct	1.11	func	phosphopantothenate--cysteine ligase	Biosynthesis of cofactors, prosthetic groups, and carriers
SAK_1153	2	0.51	funct	0.52	funct	1.08	func	phosphopantothenoylcysteine decarboxylase	Biosynthesis of cofactors, prosthetic groups, and carriers
SAK_1154	2	0.94	funct	0.32	funct	0.61	func	hypothetical protein	Cell envelope

SAK_1155	2	1.31	funct	0.88	funct	0.58	func	phosphoglucomutase/phosphomannomutase family protein	Energy metabolism
SAK_1156	1	0.43	pseudo	0.87	pseudo	1.2	func	ABC transporter, ATP-binding/permease protein	Transport and binding proteins
SAK_1158	3	0.89	funct	1.18	funct	1.49	func	hypothetical protein	Cell envelope
SAK_1159	2	0.91	funct	1.27	pseudo	1.61	func	hypothetical protein	conserved hypothetical protein
SAK_1160	2	1.14	funct	1.38	funct	1.7	func	serine hydroxymethyltransferase	Purines, pyrimidines, nucleosides, and nucleotides
SAK_1161	3	0.95	funct	1.6	funct	1.9	func	Sua5/YciO/YrdC/YwlC family protein	Unknown function
SAK_1162	1	0.81	funct	1.47	funct	1.64	func	HemK family modification methylase	Unknown function
SAK_1163	2	0.8	funct	1.49	funct	1.75	func	peptide chain release factor 1	Protein synthesis
SAK_1164	2	1.17	funct	1.44	funct	1.6	func	thymidine kinase	Purines, pyrimidines, nucleosides, and nucleotides
SAK_1165	4	3.63	funct	1.06	funct	0.83	func	4-oxalocrotonate tautomerase	Energy metabolism
SAK_1166	1	1.63	funct	1.22	funct	0.36	func	ApbE family protein	Biosynthesis of cofactors, prosthetic groups, and carriers
SAK_1167	2	1.43	funct	0.99	funct	0.28	func	NADPH-dependent FMN reductase domain-containing protein	Unknown function
SAK_1168	5	1.62	pseudo	0.97	pseudo	0.26	func	NADPH-dependent FMN reductase domain-containing protein	Unknown function
SAK_1169	2	0.83	funct	0.62	funct	0.63	func	formate/nitrite transporter family protein	Transport and binding proteins
SAK_1170	3	0.48	funct	2.11	funct	1.08	func	xanthine permease	Transport and binding proteins
SAK_1171	3	0.92	funct	2.04	funct	1.14	func	xanthine phosphoribosyltransferase	Purines, pyrimidines, nucleosides, and nucleotides
SAK_1172	4	0.5	funct	1.32	funct	1.41	func	guanosine 5'-monophosphate oxidoreductase	Purines, pyrimidines, nucleosides, and nucleotides
SAK_1173	3	0.62	pseudo	0.83	funct	0.55	func	drug:H+ antiporter-2 (DHA2) family protein	Transport and binding proteins
SAK_1174	2	0.45	funct	0.5	funct	0.88	func	hypothetical protein	conserved hypothetical protein
SAK_1175	2	0.63	pseudo	1.64	pseudo	1.03	func	K+ uptake permease (KUP) family protein	Transport and binding proteins
SAK_1176	1	0.52	pseudo	4.22	funct	0.96	func	short chain dehydrogenase/reductase family oxidoreductase	Unknown function
SAK_1177	4	0.67	pseudo	0.5	pseudo	0.78	func	phosphotransacetylase	Energy metabolism
SAK_1178	2	0.72	funct	1.29	funct	1.08	func	ribosomal large subunit pseudouridine synthase A	Protein synthesis
SAK_1179	2	0.71	funct	1.34	funct	1.03	func	inorganic polyphosphate/ATP-NAD kinase	Biosynthesis of cofactors, prosthetic groups, and carriers
SAK_1180	3	0.89	funct	1.46	funct	1.03	func	RelA/SpoT domain-containing protein	Unknown function
SAK_1181	3	0.93	funct	0.49	funct	1.12	func	hypothetical protein	Regulatory functions
SAK_1182	2	1.44	funct	0.85	funct	1.18	func	ribose-phosphate pyrophosphokinase	Purines, pyrimidines, nucleosides, and nucleotides
SAK_1183	1	1.17	funct	0.85	funct	1.1	func	cysteine desulfurase, putative	Biosynthesis of cofactors, prosthetic groups, and carriers
SAK_1185	3	1.79	funct	0.91	funct	0.68	func	redox-sensing transcriptional repressor Rex	Unknown function
SAK_1187	2	0.94	funct	1.12	funct	1.04	func	hypothetical protein	Cell envelope
SAK_1188	3	0.7	pseudo	0.87	pseudo	1.44	func	6-phospho-beta-glucosidase	Energy metabolism
SAK_1189	2	1.7	funct	0.76	funct	1.42	func	GDSL family lipase/acylhydrolase	Unknown function
SAK_1190	2	1.85	pseudo	0.59	pseudo	1.16	func	Cof-like hydrolase family protein	Unknown function
SAK_1191	2	2.99	pseudo	3.94	pseudo	0.9	func	AraC family transcriptional regulator	Regulatory functions
SAK_1192	2	1.86	pseudo	0.98	pseudo	0.85	func	chloride channel (ClC) family protein	Transport and binding proteins
SAK_1193	5	1.17	funct	1.02	funct	0.8	func	spermidine/putrescine ABC transporter, spermidine/putrescine-binding protein	Transport and binding proteins
SAK_1194	5	1.17	funct	0.86	funct	0.71	func	spermidine/putrescine ABC transporter, permease protein PotC	Transport and binding proteins
SAK_1195	3	1.4	funct	0.89	funct	0.72	func	spermidine/putrescine ABC transporter, permease protein PotB	Transport and binding proteins
SAK_1196	3	1.59	funct	0.86	funct	0.76	func	spermidine/putrescine ABC transporter, ATP-binding protein	Transport and binding proteins
SAK_1197	3	1.6	funct	0.82	funct	0.63	func	UDP-N-acetylenolpyruvoylglucosamine reductase	Cell envelope
SAK_1198	2	0.95	funct	1.11	funct	1.29	func	2-amino-4-hydroxy-6-hydroxymethyldihydropteridine pyrophosphokinase	Biosynthesis of cofactors, prosthetic groups, and carriers
SAK_1199	4	1.02	funct	0.92	funct	1.23	func	dihydroneopterin aldolase	Biosynthesis of cofactors, prosthetic groups, and carriers
SAK_1200	1	1.06	funct	0.91	funct	1.33	func	dihydropteroate synthase	Biosynthesis of cofactors, prosthetic groups, and carriers
SAK_1201	2	0.88	funct	0.83	funct	1.27	func	GTP cyclohydrolase I	Biosynthesis of cofactors, prosthetic groups, and carriers
SAK_1202	2	0.93	funct	0.78	funct	1.3	func	folylpolyglutamate synthase	Biosynthesis of cofactors, prosthetic groups, and carriers
SAK_1204	2	1.07	funct	0.66	funct	0.85	func	homoserine kinase	Amino acid biosynthesis
SAK_1206	5	2.33	pseudo	0.57	pseudo	0.8	func	polysaccharide deacetylase family protein	Energy metabolism
SAK_1211	6	2.18	funct	0.67	funct	0.58	func	aldehyde dehydrogenase family protein	Energy metabolism
SAK_1212	3	1.98	funct	1.1	funct	0.89	func	hypothetical protein	Cell envelope
SAK_1213	2	2.82	funct	2.69	funct	1.51	func	hypothetical protein	conserved hypothetical protein
SAK_1217	2	0.63	funct	0.01	funct	0.79	func	thiol peroxidase	Cellular processes
SAK_1218	2	0.67	pseudo	1.44	pseudo	0.84	func	hypothetical protein	conserved hypothetical protein
SAK_1220	1	0.46	funct	0.71	funct	0.89	func	transcriptional regulator	Regulatory functions
SAK_1221	3	0.77	funct	0.39	funct	1.03	func	alkaline shock protein 23, putative	Cellular processes
SAK_1222	2	0.48	funct	0.37	funct	1.1	func	hypothetical protein	Unknown function
SAK_1223	1	0.49	funct	0.35	funct	1.27	func	alkaline shock protein 23, putative	Cellular processes
SAK_1224	1	0.47	funct	0.32	funct	1.15	func	hypothetical protein	conserved hypothetical protein
SAK_1225	2	0.41	funct	0.27	funct	1.06	func	hypothetical protein	conserved hypothetical protein
SAK_1226	1	0.63	funct	0.18	pseudo	0.98	func	transglycosylase associated protein	Unknown function
SAK_1228	2	0.84	funct	0.65	funct	0.86	func	ATP-dependent DNA helicase PcrA	DNA metabolism
SAK_1229	2	0.23	funct	0.11	funct	0.56	func	hypothetical protein	Unknown function
SAK_1230	2	0.83	funct	0.98	funct	0.2	func	uracil permease	Transport and binding proteins
SAK_1231	4	4.16	funct	1.02	funct	0.64	func	amino acid carrier protein	Transport and binding proteins
SAK_1232	2	1.12	funct	0.65	funct	0.8	func	cation diffusion facilitator (CDF) family protein	Transport and binding proteins
SAK_1233	1	1.3	pseudo	0.86	funct	1.25	func	hypothetical protein	Unknown function
SAK_1234	2	1.18	funct	0.87	funct	1.08	func	hypothetical protein	Unknown function

SAK_1235	2	1.98	funct	0.97	funct	1.15	func	hypothetical protein	conserved hypothetical protein
SAK_1237	5	0.77	funct	0.98	funct	0.88	func	30S ribosomal protein S1	Protein synthesis
SAK_1240	2	0.67	funct	1.6	funct	1.18	func	hypothetical protein	conserved hypothetical protein
SAK_1241	7	0.59	funct	0.91	funct	0.72	func	branched-chain amino acid aminotransferase	Amino acid biosynthesis
SAK_1242	2	0.66	funct	0.56	funct	0.59	func	DNA topoisomerase IV subunit A	DNA metabolism
SAK_1243	2	0.41	funct	0.47	funct	0.68	func	DNA topoisomerase IV subunit B	DNA metabolism
SAK_1245	2	3.06	funct	2.91	funct	1.47	func	uracil-DNA glycosylase	DNA metabolism
SAK_1246	2	2.42	funct	2.32	funct	1.23	func	hypothetical protein	conserved hypothetical protein
SAK_1247	2	2.27	funct	5.71	funct	3.35	func	N-acylneuraminate cytidylyltransferase	Cell envelope
SAK_1248	2	1.99	funct	4.14	funct	2.53	func	neuD protein	Cell envelope
SAK_1249	3	2.34	funct	5.17	funct	2.63	func	UDP-N-acetylglucosamine-2-epimerase NeuC	Cell envelope
SAK_1250	2	2.95	funct	5.42	funct	2.64	func	N-acetyl neuramic acid synthetase NeuB	Cell envelope
SAK_1251	2	3.21	funct	5.9	funct	2.53	func	polysaccharide biosynthesis protein CpsL	Cell envelope
SAK_1256	2	4.33	funct	7.01	funct	3.93	func	polysaccharide biosynthesis protein CpsG	Cell envelope
SAK_1257	3	3.37	funct	5.49	funct	3.9	func	polysaccharide biosynthesis protein CpsF	Cell envelope
SAK_1259	2	1.91	funct	3.75	funct	3.37	func	tyrosine-protein kinase CpsD	Cell envelope
SAK_1261	2	2.03	funct	3.82	funct	2.9	func	protein-tyrosine phosphatase CpsB	Cell envelope
SAK_1262	2	2.52	funct	4.15	funct	2.99	func	regulatory protein CpsX	Regulatory functions
SAK_1263	2	0.81	funct	1.03	funct	1.23	func	transcriptional regulator CpsY	Regulatory functions
SAK_1265	1	0.8	funct	0.63	funct	0.84	func	purine nucleoside phosphorylase	Purines, pyrimidines, nucleosides, and nucleotides
SAK_1267	1	0.81	pseudo	0.79	pseudo	0.81	func	purine nucleoside phosphorylase	Purines, pyrimidines, nucleosides, and nucleotides
SAK_1269	1	1.23	pseudo	0.55	0	1.24	func	phosphopentomutase	Purines, pyrimidines, nucleosides, and nucleotides
SAK_1270	2	0.86	funct	0.74	funct	0.57	func	ribose-5-phosphate isomerase A	Energy metabolism
SAK_1271	1	1.25	funct	0.83	funct	0.85	func	hypothetical protein	conserved hypothetical protein
SAK_1273	3	1.36	funct	1.5	funct	1.01	func	metallo-beta-lactamase family protein	Unknown function
SAK_1274	1	0.48	pseudo	2.02	funct	0.93	func	ABC transporter, ATP-binding protein	Transport and binding proteins
SAK_1277	3	0.87	funct	0.84	funct	0.88	func	fibronectin/fibrinogen binding protein	Cellular processes
SAK_1278	4	0.67	funct	0.76	funct	0.85	func	alpha-acetolactate decarboxylase	Energy metabolism
SAK_1279	2	0.6	funct	0.61	funct	0.81	func	acetolactate synthase	Energy metabolism
SAK_1281	1	1.15	funct	0.94	funct	1.42	func	hypothetical protein	Cell envelope
SAK_1282	3	1.21	funct	0.61	funct	1.25	func	NUDIX family hydrolase	Unknown function
SAK_1283	3	1.56	funct	0.54	funct	1.17	func	mutator mutT protein	DNA metabolism
SAK_1284	2	2.28	funct	3.48	funct	2.12	func	hyaluronate lyase	Cellular processes
SAK_1285	2	1.28	funct	0.91	funct	0.86	func	dTDP-glucose 4,6-dehydratase	Cell envelope
SAK_1286	2	1.26	funct	0.81	funct	0.97	func	dTDP-4-keto-6-deoxyglucose-3,5-epimerase	Cell envelope
SAK_1287	1	1.33	funct	0.76	funct	0.96	func	glucose-1-phosphate thymidylyltransferase	Cell envelope
SAK_1288	3	2.06	funct	1.29	funct	1.08	func	oxidoreductase, FAD-binding	Unknown function
SAK_1289	2	2.24	funct	1.43	funct	1.11	func	NIF3 family protein	Unknown function
SAK_1290	2	1.77	funct	1.38	funct	1.05	func	hypothetical protein	conserved hypothetical protein
SAK_1291	2	3.48	funct	1.91	funct	1	func	DNA replication protein DnaD, putative	DNA metabolism
SAK_1292	3	2.79	funct	0.97	funct	0.92	func	adenine phosphoribosyltransferase	Purines, pyrimidines, nucleosides, and nucleotides
SAK_1293	3	0.48	pseudo	1.72	funct	0.55	func	hydrophobic W repeat-containing protein	Unknown function
SAK_1294	2	1.21	funct	1.72	funct	1.38	func	single-stranded-DNA-specific exonuclease RecJ	DNA metabolism
SAK_1295	1	0.89	funct	0.85	funct	1.3	func	short chain dehydrogenase/reductase family oxidoreductase	Unknown function
SAK_1296	2	0.72	funct	0.92	funct	1.57	func	ribonuclease Z	Transcription
SAK_1297	3	0.73	funct	1	funct	1.54	func	hypothetical protein	conserved hypothetical protein
SAK_1298	3	1.21	funct	1.58	funct	1.58	func	GTP-binding protein	Unknown function
SAK_1299	2	1.68	funct	1.17	funct	1.31	func	tRNA delta(2)-isopentenylpyrophosphate transferase	Protein synthesis
SAK_1300	2	3.68	funct	0.63	funct	1.05	func	hypothetical protein	hypothetical protein
SAK_1303	2	1.5	funct	1.08	funct	1.41	func	hypothetical protein	Unknown function
SAK_1304	1	1.41	funct	0.9	funct	1.56	func	hypothetical protein	DNA metabolism
SAK_1305	1	1.52	funct	0.44	funct	0.85	func	dipeptidase PepV	Protein fate
SAK_1306	7	1.28	funct	0.39	funct	0.85	func	nitroreductase family protein	Unknown function
SAK_1307	4	1.11	funct	1.11	funct	1.04	func	glycerophosphoryl diester phosphodiesterase family protein	Fatty acid and phospholipid metabolism
SAK_1309	4	1.67	funct	1.82	funct	0.89	func	hypothetical protein	conserved hypothetical protein
SAK_1310	2	0.35	funct	0.44	funct	1.03	func	MATE efflux family protein	Transport and binding proteins
SAK_1311	1	0.23	pseudo	0.28	pseudo	1.03	func	hypothetical protein	Unknown function
SAK_1312	3	0.24	funct	0.2	funct	0.94	func	hypothetical protein	conserved hypothetical protein
SAK_1313	2	1.27	funct	0.55	funct	1.21	func	hypothetical protein	conserved hypothetical protein
SAK_1334	4	0.18	funct	0.72	funct	0.68	func	50S ribosomal protein L7/L12	Protein synthesis
SAK_1335	7	0.17	funct	0.86	funct	0.73	func	50S ribosomal protein L10	Protein synthesis
SAK_1336	2	2.06	funct	16.38	funct	1.64	func	ATP-dependent Clp protease, ATP-binding subunit ClpL	Protein fate
SAK_1337	1	0.79	funct	1.43	funct	1.32	func	homocysteine methyltransferase	Amino acid biosynthesis
SAK_1338	2	1.48	pseudo	1.35	funct	0.99	func	amino acid permease	Transport and binding proteins
SAK_1340	1	1.15	funct	0.76	funct	0.96	func	hypothetical protein	hypothetical protein
SAK_1341	1	0.18	funct	0.1	funct	0.61	func	TetR family transcriptional regulator	Regulatory functions
SAK_1342	1	0.9	funct	0.46	funct	0.95	func	ribosome biogenesis GTP-binding protein YsxC	Unknown function
SAK_1343	3	0.98	funct	0.47	funct	0.93	func	ATP-dependent protease ATP-binding subunit ClpX	Protein fate
SAK_1345	2	0.83	funct	0.46	funct	0.93	func	dihydrofolate reductase	Biosynthesis of cofactors, prosthetic groups, and carriers
SAK_1346	3	0.66	funct	0.57	funct	0.82	func	thymidylate synthase	Purines, pyrimidines, nucleosides, and nucleotides
SAK_1347	2	1.84	funct	0.48	funct	0.85	func	hydroxymethylglutaryl-CoA synthase	Biosynthesis of cofactors, prosthetic groups, and carriers
SAK_1348	2	0.86	funct	0.51	funct	0.92	func	hydroxymethylglutaryl-CoA reductase, degradative	Energy metabolism
SAK_1349	2	0.25	funct	0.12	funct	0.94	func	hypothetical protein	conserved hypothetical protein
SAK_1350	2	0.35	funct	0.12	funct	0.71	func	hemolysin III	Cellular processes

SAK_1351	2	0.55	funct	0.23	funct	0.71	func	hypothetical protein	conserved hypothetical protein TIGR00147
SAK_1353	2	0.75	funct	1.3	funct	0.74	func	hypothetical protein	conserved hypothetical protein
SAK_1354	3	0.68	funct	1.22	pseudo	0.8	func	isopentenyl pyrophosphate isomerase	Biosynthesis of cofactors, prosthetic groups, and carriers
SAK_1355	1	0.64	funct	1.18	funct	0.92	func	phosphomevalonate kinase	Central intermediary metabolism
SAK_1356	2	0.88	funct	0.96	funct	0.8	func	diphosphomevalonate decarboxylase	Central intermediary metabolism
SAK_1357	2	0.91	funct	0.97	funct	0.75	func	mevalonate kinase	Central intermediary metabolism
SAK_1359	4	1.99	funct	2.82	funct	2.47	func	DNA-binding response regulator	Signal transduction
SAK_1360	3	2.97	funct	2.81	funct	2.49	func	RelA/SpoT domain-containing protein	Unknown function
SAK_1367	2	0.33	pseudo	0.03	pseudo	0.63	func	hypothetical protein	Cell envelope
SAK_1368	4	2.13	funct	2.9	funct	1.03	func	ABC transporter, ATP-binding/permease protein	Transport and binding proteins
SAK_1369	2	1.96	funct	2.2	funct	1.07	func	ABC transporter, ATP-binding/permease protein	Transport and binding proteins
SAK_1370	2	1.66	pseudo	2.37	funct	1.61	func	acetyltransferase	Unknown function
SAK_1371	2	1.45	funct	2.51	funct	1.82	func	ABC transporter, ATP-binding protein	Transport and binding proteins
SAK_1372	1	1.3	funct	2.65	funct	1.78	func	tRNA CCA-pyrophosphorylase	Transcription
SAK_1373	6	1.51	funct	2.91	funct	1.29	func	DegV family protein	Unknown function
SAK_1374	1	1.76	funct	3.67	funct	1.55	func	hypothetical protein	conserved hypothetical protein
SAK_1375	3	1.81	funct	5.65	funct	1.63	func	hypothetical protein	conserved domain protein
SAK_1376	2	2.29	funct	1.13	funct	1.69	func	hypothetical protein	conserved hypothetical protein
SAK_1377	6	0.64	pseudo	1.13	funct	0.47	func	PTS system, fructose-specific IIABC component	Signal transduction
SAK_1378	3	0.91	funct	0.97	funct	0.63	func	1-phosphofructokinase, putative	Energy metabolism
SAK_1379	3	0.7	funct	0.75	funct	0.56	func	DeoR family transcriptional regulator	Regulatory functions
SAK_1382	2	1.66	pseudo	1.16	funct	1.08	func	2-dehydropantoate 2-reductase	Biosynthesis of cofactors, prosthetic groups, and carriers
SAK_1383	1	1.86	funct	0.92	funct	1.05	func	hypothetical protein	Cell envelope
SAK_1384	3	0.97	funct	1.22	funct	1.57	func	pyridine nucleotide-disulphide oxidoreductase family protein	Unknown function
SAK_1385	3	0.92	funct	1.13	funct	1.52	func	tRNA (guanine-N(1)-)-methyltransferase	Protein synthesis
SAK_1386	3	1.18	funct	1.21	funct	1.48	func	16S rRNA-processing protein RimM	Transcription
SAK_1389	3	2.1	pseudo	0.98	pseudo	1.73	func	RofA family transcriptional regulator	Regulatory functions
SAK_1390	3	0.38	funct	0.8	funct	0.71	func	hypothetical protein	conserved hypothetical protein
SAK_1391	2	0.42	funct	0.74	funct	0.71	func	30S ribosomal protein S16	Protein synthesis
SAK_1392	4	1.36	funct	0.15	funct	1.99	func	ABC transporter, permease protein, putative	Transport and binding proteins
SAK_1393	2	1.07	funct	0.08	funct	2.83	func	ABC transporter, ATP-binding protein	Transport and binding proteins
SAK_1394	2	1.53	pseudo	0.08	funct	2.2	func	RND family efflux transporter MFP subunit	Transport and binding proteins
SAK_1395	4	1.97	funct	1.7	funct	0.96	func	carbamoyl-phosphate synthase, large subunit, putative	Purines, pyrimidines, nucleosides, and nucleotides
SAK_1396	2	1.63	funct	1.39	pseudo	0.96	func	carbamoyl phosphate synthase small subunit	Purines, pyrimidines, nucleosides, and nucleotides
SAK_1397	3	1.74	funct	1.21	funct	0.96	func	bifunctional pyrimidine regulatory protein PyrR uracil phosphoribosyltransferase	Regulatory functions
SAK_1398	1	1.14	funct	0.98	funct	0.96	func	ribosomal large subunit pseudouridine synthase A	Protein synthesis
SAK_1399	1	1.05	funct	1.16	funct	1.19	func	signal peptidase II	Protein fate
SAK_1400	2	1.06	funct	1.04	funct	1.12	func	LysR family transcriptional regulator	Regulatory functions
SAK_1401	2	0.74	funct	0.75	funct	0.79	func	50S ribosomal protein L27	Protein synthesis
SAK_1402	2	0.64	funct	0.62	funct	0.74	func	hypothetical protein	conserved hypothetical protein
SAK_1403	3	0.79	funct	0.68	funct	0.71	func	50S ribosomal protein L21	Protein synthesis
SAK_1404	2	1.58	funct	0.82	pseudo	0.81	func	CapA domain-containing protein	Unknown function
SAK_1405	2	1.08	funct	1.13	pseudo	0.84	func	thiamine biosynthesis protein ThiI	Biosynthesis of cofactors, prosthetic groups, and carriers
SAK_1406	3	1.25	funct	0.9	funct	0.96	func	aminotransferase, class V	Unknown function
SAK_1407	2	1.03	funct	0.45	funct	1.16	func	hypothetical protein	conserved hypothetical protein
SAK_1409	2	1	funct	0.51	funct	1.1	func	hypothetical protein	conserved hypothetical protein
SAK_1410	2	0.82	funct	1.94	funct	1.59	func	chorismate synthase	Amino acid biosynthesis
SAK_1411	3	0.89	funct	1.95	funct	1.53	func	3-dehydroquinate synthase	Amino acid biosynthesis
SAK_1412	2	0.75	funct	1.86	funct	1.27	func	3-dehydroquinate dehydratase	Amino acid biosynthesis
SAK_1413	3	1.03	funct	1.82	funct	1.44	func	hypothetical protein	conserved hypothetical protein
SAK_1414	3	0.79	funct	0.58	funct	1.08	func	sulfatase family protein	Unknown function
SAK_1415	4	1.1	funct	1.28	funct	0.62	func	50S ribosomal protein L20	Protein synthesis
SAK_1416	2	0.92	funct	0.82	funct	0.71	func	50S ribosomal protein L35	Protein synthesis
SAK_1417	3	1.12	funct	0.48	funct	0.68	func	translation initiation factor IF-3	Protein synthesis
SAK_1418	3	2.51	funct	1.12	funct	1.01	func	cytidylate kinase	Purines, pyrimidines, nucleosides, and nucleotides
SAK_1419	3	2.19	funct	1	funct	1.16	func	hypothetical protein	conserved hypothetical protein
SAK_1420	2	1.53	funct	1.36	funct	0.94	func	ferredoxin, putative	Energy metabolism
SAK_1421	4	1.54	funct	0.77	funct	1.15	func	hypothetical protein	conserved hypothetical protein
SAK_1422	2	1.88	funct	0.76	funct	1.07	func	peptidase T	Protein fate
SAK_1423	2	0.9	funct	1.08	funct	0.74	func	polysaccharide biosynthesis protein	Cell envelope
SAK_1425	1	0.4	funct	4.54	funct?	1.71	func	ferrichrome ABC transporter, ATP-binding protein	Transport and binding proteins
SAK_1426	1	0.22	funct	5.08	funct	2.23	func	ferrichrome ABC transporter, ferrichrome-binding protein	Transport and binding proteins
SAK_1427	1	0.39	pseudo	3.49	pseudo	1.9	func	ferrichrome ABC transporter, permease protein	Transport and binding proteins
SAK_1428	1	0.69	pseudo	2.45	funct	1.71	func	iron compound ABC transporter, permease protein	Transport and binding proteins
SAK_1430	3	1.63	funct	0.77	funct	0.8	func	putative manganese-dependent inorganic pyrophosphatase	Central intermediary metabolism
SAK_1431	5	1.71	funct	1.47	funct	0.45	func	pyruvate formate-lyase-activating enzyme	Energy metabolism
SAK_1432	1	1.5	funct	1.58	funct	1.07	func	CBS domain-containing protein	Unknown function
SAK_1433	3	1.72	funct	2.28	funct	1.16	func	hypothetical protein	conserved hypothetical protein
SAK_1434	2	2.07	funct	2.45	funct	1	func	radical SAM family protein	Unknown function
SAK_1435	1	6.61	funct	9.74	funct	0.61	func	PAP2 family protein	Unknown function
SAK_1436	3	7.85	funct	7.06	funct	0.83	func	hypothetical protein	Cell envelope

SAK_1437	1	0.05	pseudo	0.05	funct	1.71	func	sortase family protein, putative	Protein fate
SAK_1441	5	0.06	0	0.02	pseudo	1.28	func	Cna B domain-containing protein	Unknown function
SAK_1442	3	0.12	0	0.05	pseudo	1.55	func	hypothetical protein	hypothetical protein
SAK_1443	2	0.17	funct	0.06	funct	1.34	func	signal peptidase I	Protein fate
SAK_1444	4	0.16	funct	0.05	funct	0.9	func	hypothetical protein	hypothetical protein
SAK_1445	1	0.96	funct	2.58	funct	1.11	func	glycosyl transferase, group 1 family protein	Cell envelope
SAK_1446	2	0.84	funct	1.91	funct	0.88	func	glycosyl transferase, group 2 family protein	Cell envelope
SAK_1447	5	0.74	funct	1.31	funct	0.74	func	polysaccharide biosynthesis protein	Cell envelope
SAK_1448	1	0.71	funct	1.24	funct	0.73	func	hypothetical protein	Cell envelope
SAK_1449	2	0.83	funct	1.38	funct	1	func	glycosyl transferase, group 2 family protein	Cell envelope
SAK_1450	3	0.9	funct	1.2	funct	1	func	glycosyl transferase, group 2 family protein	Cell envelope
SAK_1452	2	1.01	funct	1.04	funct	1.1	func	2-C-methyl-D-erythritol 4-phosphate cytidylyltransferase	Biosynthesis of cofactors, prosthetic groups, and carriers
SAK_1453	7	1.01	funct	1.16	funct	1.16	func	licD2 protein	Cell envelope
SAK_1454	2	0.79	funct	1.26	funct	1.58	func	hypothetical protein	hypothetical protein
SAK_1455	2	1.42	funct	1.5	funct	1.24	func	hypothetical protein	conserved hypothetical protein
SAK_1456	2	1.46	funct	1.67	funct	1.33	func	glycosyl transferase, group 2 family protein	Cell envelope
SAK_1457	4	1	funct	1.4	funct	1.12	func	glycosyl transferase, group 2 family protein	Cell envelope
SAK_1458	4	1.61	funct	1.57	funct	1.01	func	rgpAc protein	Cell envelope
SAK_1459	2	1.52	funct	1.02	funct	0.93	func	dTDP-4-dehydrorhamnose reductase	Cell envelope
SAK_1460	2	1.16	funct	1.45	funct	1.19	func	hypothetical protein	conserved hypothetical protein
SAK_1461	1	1.31	funct	1.39	funct	1.29	func	RNA polymerase sigma factor RpoD	Transcription
SAK_1462	3	1.54	funct	1.09	funct	1.07	func	DNA primase	DNA metabolism
SAK_1463	2	0.85	funct	0.46	funct	0.39	func	large conductance mechanosensitive channel protein	Cellular processes
SAK_1464	2	0.66	funct	0.82	funct	0.93	func	30S ribosomal protein S21	Protein synthesis
SAK_1466	1	2.33	funct	8.38	funct	0.84	func	polar amino acid ABC transporter amino acid-binding protein	Transport and binding proteins
SAK_1467	1	1.89	funct	1.83	funct	1.05	func	ammonium transporter (Amt) family protein	Transport and binding proteins
SAK_1469	1	0.95	funct	0.85	funct	1.06	func	hypothetical protein	Unknown function
SAK_1470	4	1.2	pseudo	0.45	pseudo	0.65	func	hypothetical protein	conserved hypothetical protein
SAK_1471	1	0.25	pseudo	0.24	pseudo	1.06	func	glycerol-3-phosphate transporter, putative	Transport and binding proteins
SAK_1472	2	0.28	funct	0.13	funct	0.27	func	glycogen/starch/alpha-glucan phosphorylase	Energy metabolism
SAK_1473	2	0.41	funct	0.11	funct	0.28	func	4-alpha-glucanotransferase	Energy metabolism
SAK_1474	2	0.32	funct	2.37	funct	0.39	func	maltose operon transcriptional repressor MalR, putative	Regulatory functions
SAK_1475	6	0.03	funct	0.14	funct	0.2	func	carbohydrate ABC transporter periplasmic-binding protein	Transport and binding proteins
SAK_1476	5	0.15	pseudo	0.22	funct	0.23	func	cyclodextrin ABC transporter, permease protein	Transport and binding proteins
SAK_1477	2	0.13	funct	0.19	funct	0.21	func	cyclodextrin ABC transporter, permease protein	Transport and binding proteins
SAK_1478	4	0.69	pseudo	1.86	pseudo	0.73	func	amino acid/peptide transporter	Transport and binding proteins
SAK_1479	1	0.49	funct	1.18	funct	2.09	func	NUDIX family hydrolase	Unknown function
SAK_1480	1	0.45	pseudo	1.01	funct	2.38	func	hypothetical protein	conserved hypothetical protein
SAK_1481	1	0.46	pseudo	0.93	funct	2.3	func	glycosyl transferase, group 1 family protein	Cell envelope
SAK_1482	1	0.89	funct	0.81	pseudo	1.9	func	preprotein translocase subunit SecA	Protein fate
SAK_1483	2	0.7	funct	0.87	funct	2.48	func	hypothetical protein	Protein fate
SAK_1485	1	0.64	funct	0.94	pseudo	3.52	func	hypothetical protein	Protein fate
SAK_1492	1	1	funct	0.83	funct	3.76	func	hypothetical protein	conserved hypothetical protein
SAK_1493	1	0.16	funct	0.24	func	23.73	func	cell wall surface anchor family protein	Cell envelope
SAK_1495	4	0.84	funct	0.58	funct	0.99	func	excinuclease ABC subunit B	DNA metabolism
SAK_1496	2	2.12	pseudo	3.73	pseudo	0.95	func	CAAX amino terminal protease family protein	Unknown function
SAK_1497	3	0.74	funct	0.86	funct	1.02	func	polar amino acid ABC transporter permease/substrate-binding protein	Transport and binding proteins
SAK_1498	2	0.97	funct	0.95	funct	0.89	func	polar amino acid ABC transporter ATP-binding protein	Transport and binding proteins
SAK_1499	2	2.15	funct	0.79	funct	0.74	func	hypothetical protein	conserved hypothetical protein
SAK_1500	1	1.92	funct	0.78	funct	0.88	func	GTPase ObgE	Unknown function
SAK_1501	2	2.47	funct	1.31	funct	0.66	func	hypothetical protein	conserved hypothetical protein
SAK_1502	2	0.56	pseudo	1.71	funct	0.62	func	aminopeptidase PepS	Protein fate
SAK_1503	4	0.86	funct	0.57	funct	1.19	func	cell wall surface anchor family protein	Cell envelope
SAK_1504	3	1.32	funct	0.76	funct	1.21	func	amidase family protein	Unknown function
SAK_1506	2	1.41	funct	0.26	funct	0.92	func	aldo/keto reductase family oxidoreductase	Unknown function
SAK_1507	2	1.84	funct	0.66	funct	1.01	func	nitroreductase family protein	Unknown function
SAK_1508	2	2.36	funct	0.63	funct	0.83	func	lactoylglutathione lyase	Energy metabolism
SAK_1509	2	1.77	funct	0.82	funct	0.77	func	glycosyl transferase, group 2 family protein	Cell envelope
SAK_1510	2	0.66	funct	0.39	funct	0.84	func	D-Serine/D-alanine/glycine:H+ symporter	Transport and binding proteins
SAK_1511	2	1.64	funct	3.82	funct	1.38	func	SsrA-binding protein	Protein synthesis
SAK_1512	3	1.64	funct	3.59	funct	1.24	func	ribonuclease R	Transcription
SAK_1513	4	1.18	funct	2.39	funct	0.95	func	preprotein translocase subunit SecG	Protein fate
SAK_1515	2	0.72	funct	0.63	pseudo	0.92	func	drug:H+ antiporter-1 (DHA1) family protein	Transport and binding proteins
SAK_1516	2	1.55	pseudo	0.12	funct	1.82	func	hypothetical protein	Cell envelope
SAK_1517	2	1.55	funct	0.17	funct	1.38	func	ABC transporter, ATP binding protein	Transport and binding proteins
SAK_1518	3	1.87	funct	0.67	funct	1.18	func	dephospho-CoA kinase	Biosynthesis of cofactors, prosthetic groups, and carriers
SAK_1519	1	1.43	funct	0.62	funct	0.74	func	formamidopyrimidine-DNA glycosylase	DNA metabolism
SAK_1521	2	0.42	funct	0.11	pseudo	0.99	func	hypothetical protein	hypothetical protein
SAK_1524	2	0.46	pseudo	1.57	funct	1	func	hypothetical protein	conserved hypothetical protein
SAK_1525	2	1.04	funct	1.17	funct	1.23	func	GTP-binding protein Era	Regulatory functions
SAK_1526	2	1.38	funct	1.04	funct	1.02	func	diacylglycerol kinase	Fatty acid and phospholipid metabolism
SAK_1527	2	1.43	funct	1.12	funct	1.03	func	putative metalloprotease	conserved hypothetical protein TIGR00043
SAK_1528	1	1.16	funct	0.77	funct	1.49	func	tetracenomycin polyketide synthesis O-methyltransferase TcmP, putative	Cellular processes
SAK_1530	2	1.03	funct	1.14	funct	1.17	func	hypothetical protein	conserved hypothetical protein
SAK_1531	2	2.37	funct	0.64	funct	0.8	func	KH domain-containing protein	Unknown function
SAK_1532	1	2.16	funct	0.28	funct	1.07	func	myosin-cross-reactive antigen	Cellular processes
SAK_1533	1	0.76	funct	1.26	funct	1	func	hypothetical protein	conserved hypothetical protein

SAK_1534	2	0.85	funct	1.21	funct	1.01	func	methionine sulfoxide reductase A	Protein fate
SAK_1535	2	0.7	funct	1.34	funct	0.79	func	hypothetical protein	conserved hypothetical protein
SAK_1536	3	0.71	funct	1.71	funct	1.13	func	ribosome recycling factor	Protein synthesis
SAK_1537	8	0.78	funct	1.49	funct	0.95	func	uridylate kinase	Purines, pyrimidines, nucleosides, and nucleotides
SAK_1538	2	1.13	funct	16.87	funct	1.37	func	nickel ABC transporter, ATP-binding protein	Transport and binding proteins
SAK_1543	3	0.35	funct	0.79	funct	0.78	func	50S ribosomal protein L1	Protein synthesis
SAK_1544	3	0.47	funct	0.71	funct	0.91	func	50S ribosomal protein L11	Protein synthesis
SAK_1546	3	0.71	funct	1.29	funct	1.48	func	major facilitator family protein	Transport and binding proteins
SAK_1547	2	0.76	funct	1.22	funct	1.36	func	M20D family peptidase	Protein fate
SAK_1548	2	0.36	funct	0.81	funct	1.5	func	LysR family transcriptional regulator	Regulatory functions
SAK_1551	4	2.06	funct	1.66	funct	1.1	func	para-aminobenzoate synthase, component I	Biosynthesis of cofactors, prosthetic groups, and carriers
SAK_1552	1	0.93	funct	1.01	funct	0.67	func	DNA translocase FtsK	Cellular processes
SAK_1553	2	1.37	funct	0.75	funct	0.75	func	peptidyl-prolyl cis-trans isomerase, cyclophilin-type	Protein fate
SAK_1554	2	1.84	funct	2.27	funct	0.72	func	metal ABC transporter, permease protein	Transport and binding proteins
SAK_1555	3	1.49	funct	1.84	funct	0.88	func	metal ABC transporter, ATP-binding protein	Transport and binding proteins
SAK_1556	3	0.62	funct	0.69	funct	0.8	func	metal ABC transporter, metal-binding lipoprotein	Transport and binding proteins
SAK_1557	3	1.31	funct	1.08	funct	1.11	func	iron-dependent repressor	Regulatory functions
SAK_1558	3	1.67	funct	3.11	funct	1.39	func	5'-methylthioadenosine/S-adenosylhomocysteine nucleosidase	Purines, pyrimidines, nucleosides, and nucleotides
SAK_1559	3	1.45	funct	3.25	funct	1.37	func	hypothetical protein	conserved hypothetical protein
SAK_1560	2	1.37	funct	3.27	funct	1.45	func	NUDIX family hydrolase	Unknown function
SAK_1561	2	1.41	funct	3.2	funct	1.43	func	UDP-N-acetylglucosamine pyrophosphorylase	Cell envelope
SAK_1562	2	8.45	funct	14.7	funct	1.75	func	hypothetical protein	conserved hypothetical protein
SAK_1563	3	6.01	funct	9.45	funct	1.57	func	glyoxalase family protein	Unknown function
SAK_1564	2	1.45	funct	1.18	funct	1.37	func	Gfo/Idh/MocA family oxidoreductase	Unknown function
SAK_1566	1	0.97	funct	1.06	funct	1.76	func	3-ketoacyl-(acyl-carrier-protein) reductase	Unknown function
SAK_1567	2	1.04	funct	1.01	funct	1.64	func	hypothetical protein	conserved hypothetical protein
SAK_1568	1	0.72	funct	0.94	funct	1.4	func	hypothetical protein	conserved domain protein
SAK_1569	2	0.93	funct	0.82	funct	1.86	func	acetyltransferase	Unknown function
SAK_1575	4	1.06	funct	0.74	pseudo	0.96	func	branched chain amino acid:H+ symporter	Transport and binding proteins
SAK_1576	2	0.81	funct	0.55	funct	1.04	func	methionyl-tRNA synthetase	Protein synthesis
SAK_1577	2	1.57	funct	0.83	pseudo	0.84	func	tellurite resistance protein TehB	Cellular processes
SAK_1578	1	0.47	funct	1.03	funct	1.12	func	hypothetical protein	Cell envelope
SAK_1579	4	0.93	funct	0.47	funct	0.79	func	PTS system putative lactose/cellobiose family IIC subunit	Signal transduction
SAK_1580	3	1.02	funct	0.59	funct	0.97	func	hypothetical protein	conserved hypothetical protein
SAK_1581	4	1.16	funct	0.79	funct	0.74	func	exodeoxyribonuclease III	DNA metabolism
SAK_1582	2	1.97	funct	1.27	funct	0.73	func	hypothetical protein	Unknown function
SAK_1583	2	1.55	funct	1.1	funct	0.84	func	methylated-DNA--protein-cysteine S-methyltransferase, putative	DNA metabolism
SAK_1584	4	1.44	funct	2.42	funct	0.98	func	D-3-phosphoglycerate dehydrogenase, putative	Amino acid biosynthesis
SAK_1585	2	1.37	funct	2.03	funct	1.1	func	acetyltransferase	Unknown function
SAK_1587	4	1.74	funct	0.82	funct	0.7	func	CutC family protein	Cellular processes
SAK_1588	2	0.95	funct	1.08	funct	1.05	func	tetrapyrrole methylase family protein	Unknown function
SAK_1589	2	0.93	funct	0.99	funct	1.11	func	DNA replication intiation control protein YabA	conserved hypothetical protein
SAK_1590	2	1.28	funct	0.98	funct	1.04	func	DNA polymerase III subunit delta'	DNA metabolism
SAK_1591	2	1.37	funct	0.95	funct	1.05	func	thymidylate kinase	Purines, pyrimidines, nucleosides, and nucleotides
SAK_1593	3	1.08	funct	1	funct	0.79	func	CBS domain-containing protein	Unknown function
SAK_1599	3	1.74	funct	2.37	funct	0.71	func	hypothetical protein	conserved hypothetical protein
SAK_1600	5	1.14	funct	1.4	funct	0.79	func	ATP-dependent Clp protease proteolytic subunit	Protein fate
SAK_1601	3	1.51	funct	1.31	funct	0.89	func	uracil phosphoribosyltransferase	Purines, pyrimidines, nucleosides, and nucleotides
SAK_1602	1	0.86	funct	0.39	funct	1.31	func	aminotransferase, classes I and II	Unknown function
SAK_1603	2	2.18	funct	1.57	funct	0.64	func	RNA methyltransferase	Protein synthesis
SAK_1604	1	0.86	funct	1.04	funct	0.8	func	amino acid-polyamine-organocation (APC) family permease	Transport and binding proteins
SAK_1606	4	0.11	funct	0.53	pseudo	0.82	func	K+ transporter (Trk) family protein TrkH, putative	Transport and binding proteins
SAK_1607	1	0.96	funct	2.33	funct	1.12	func	hypothetical protein	conserved hypothetical protein TIGR00278
SAK_1608	3	1.09	funct	1.87	funct	1.08	func	ribosomal large subunit pseudouridine synthase B	Protein synthesis
SAK_1609	5	0.97	funct	1.94	funct	1.37	func	segregation and condensation protein B	Cellular processes
SAK_1610	2	1.04	funct	2.27	funct	1.61	func	segregation and condensation protein A	Cellular processes
SAK_1611	3	1.22	funct	2.18	funct	1.6	func	site-specific tyrosine recombinase XerD-like protein	DNA metabolism
SAK_1612	3	1.51	funct	1.88	funct	1.44	func	CBS domain-containing protein	Unknown function
SAK_1613	1	1.34	funct	1.85	funct	1.18	func	hypothetical protein	Unknown function
SAK_1614	2	1.24	funct	1.9	funct	1.11	func	putative deoxyribonucleotide triphosphate pyrophosphatase/unknown domain fusion protein	DNA metabolism
SAK_1615	1	1.67	funct	2	funct	0.95	func	glutamate racemase	Cell envelope
SAK_1616	3	1.69	funct	1.77	funct	0.86	func	hypothetical protein	conserved hypothetical protein
SAK_1618	3	2.17	funct	1.94	funct	1.07	func	3H domain-containing protein	Unknown function
SAK_1619	2	1.59	funct	1.53	funct	1.54	func	hypothetical protein	Cell envelope
SAK_1620	2	1.93	funct	1.63	funct	1.61	func	hypothetical protein	conserved hypothetical protein
SAK_1621	2	0.5	funct	0.41	funct	1.29	func	RNA methyltransferase	Protein synthesis
SAK_1622	2	1.99	funct	0.47	funct	1.06	func	acylphosphatase	Fatty acid and phospholipid metabolism
SAK_1623	1	0.94	funct	1.26	funct	1.02	func	OxaA-like protein precursor	Cell envelope
SAK_1624	2	2.12	funct	5.76	funct	1.44	func	polar amino acid ABC transporter permease	Transport and binding proteins
SAK_1625	2	1.84	funct	5.44	funct	1.45	func	polar amino acid ABC transporter polar amino acid-binding protein	Transport and binding proteins

SAK_1626	1	2.3	pseudo	4.59	funct	1.02	func	amidase	Unknown function
SAK_1627	1	0.86	funct	2.01	funct	1.09	func	transcription elongation factor GreA	Transcription
SAK_1628	3	0.85	funct	1.6	funct	1.07	func	hypothetical protein	conserved hypothetical protein TIGR00247
SAK_1629	2	0.88	funct	1.31	funct	0.82	func	acetyltransferase	Unknown function
SAK_1630	2	0.63	funct	1.31	funct	1.23	func	UDP-N-acetylmuramate--L-alanine ligase	Cell envelope
SAK_1631	2	0.5	funct	1.12	funct	1.45	func	hypothetical protein	conserved hypothetical protein
SAK_1633	3	0.6	funct	1.03	funct	1.41	func	Snf2 family protein	Unknown function
SAK_1634	2	1.61	funct	1.2	funct	1.1	func	GTP-binding protein EngA	Unknown function
SAK_1635	2	1.37	funct	1.45	funct	1.33	func	primosomal protein DnaI	DNA metabolism
SAK_1636	2	1.29	funct	1.69	funct	1.46	func	hypothetical protein	DNA metabolism
SAK_1637	4	1.9	funct	1.43	funct	1.23	func	transcriptional regulator NrdR	Unknown function
SAK_1638	1	1.29	funct	2.83	funct	1.15	func	sensor histidine kinase CsrS, putative	Signal transduction
SAK_1639	3	0.77	funct	0.49	funct	1.21	func	DNA-binding response regulator CsrR	Signal transduction
SAK_1640	1	1.76	funct	0.56	funct	1.29	func	hypothetical protein	conserved hypothetical protein
SAK_1641	2	2.08	funct	1.31	funct	0.66	func	heat shock protein HtpX	Protein fate
SAK_1642	3	1.43	funct	0.74	funct	0.77	func	LemA family protein	Unknown function
SAK_1643	4	1.32	funct	0.96	funct	0.8	func	16S rRNA methyltransferase GidB	Unknown function
SAK_1644	2	0.79	funct	0.84	funct	0.94	func	K+ transporter (Trk) family protein	Transport and binding proteins
SAK_1645	1	0.96	funct	0.71	funct	0.92	func	Trk family potassium uptake protein	Transport and binding proteins
SAK_1646	1	0.55	funct	1.03	funct	0.88	func	nickel/cobalt ABC transporter permease protein	Transport and binding proteins
SAK_1647	1	0.38	funct	1.1	pseudo	0.92	func	ABC transporter, ATP-binding protein	Transport and binding proteins
SAK_1648	1	0.55	funct	1.22	funct	0.84	func	hypothetical protein	Cell envelope
SAK_1649	1	1.3	funct	1.43	funct	1.4	func	serine/threonine transporter SstT	Transport and binding proteins
SAK_1651	1	1.57	funct	4	funct	0.49	func	alcohol dehydrogenase, zinc-containing	Energy metabolism
SAK_1652	3	0.7	funct	6.19	funct	0.76	func	D-methionine ABC transporter, permease protein	Transport and binding proteins
SAK_1653	3	0.63	funct	7.54	funct	0.84	func	amino acid ABC transporter, ATP-binding protein, putative	Transport and binding proteins
SAK_1654	4	0.66	funct	8.04	funct	0.91	func	hypothetical protein	Protein fate
SAK_1655	2	0.74	funct	7.5	funct	1.3	func	NLPA family lipoprotein	Cell envelope
SAK_1656	2	0.32	funct	1.33	funct	1.14	func	amino acid ABC transporter, amino acid-binding protein, putative	Transport and binding proteins
SAK_1657	2	0.29	funct	1.5	funct	1.13	func	glutamine amidotransferase class I	Unknown function
SAK_1658	2	0.18	funct	0.53	funct	1.04	func	hypothetical protein	conserved hypothetical protein TIGR01033
SAK_1659	1	0.59	pseudo	0.81	funct	0.88	func	dihydroxyacetone kinase DAK1 domain-containing protein	Fatty acid and phospholipid metabolism
SAK_1662	1	0.18	pseudo	1.6	funct	0.38	func	dihydroxyacetone kinase DAK1 domain-containing protein	Fatty acid and phospholipid metabolism
SAK_1663	2	0.1	funct	1.82	funct	0.39	func	dihydroxyacetone kinase DAK2 domain-containing protein	Fatty acid and phospholipid metabolism
SAK_1664	1	0.1	funct	2.3	funct	0.39	func	hypothetical protein	conserved hypothetical protein
SAK_1665	1	0.19	funct	2.65	funct	0.33	func	glycerol uptake facilitator protein	Transport and binding proteins
SAK_1666	2	2.41	funct	0.78	funct	0.65	func	hypothetical protein	conserved hypothetical protein
SAK_1667	2	1.04	funct	2.01	funct	1.37	func	MerR family transcriptional regulator	Regulatory functions
SAK_1668	3	0.91	funct	1.85	funct	1.2	func	hypothetical protein	conserved hypothetical protein
SAK_1669	3	1.03	funct	1.64	funct	1.13	func	hypothetical protein	hypothetical protein
SAK_1670	3	0.94	funct	1.8	funct	1.02	func	hypothetical protein	conserved hypothetical protein
SAK_1671	3	0.81	funct	1.43	funct	1.46	func	iojap-related protein	Unknown function
SAK_1673	2	0.75	funct	1.47	funct	1.46	func	HD domain-containing protein	Unknown function
SAK_1674	2	0.7	funct	1.29	funct	1.61	func	nicotinic acid mononucleotide adenylyltransferase	Biosynthesis of cofactors, prosthetic groups, and carriers
SAK_1675	1	0.46	funct	1.3	funct	1.84	func	hypothetical protein	conserved hypothetical protein TIGR00253
SAK_1676	2	0.68	funct	1.23	funct	1.61	func	GTP-binding protein YqeH	Unknown function
SAK_1677	3	1.17	funct	1.08	funct	1.27	func	HAD superfamily hydrolase	Unknown function
SAK_1678	3	1.6	funct	1.61	pseudo	1.07	func	hypothetical protein	Cell envelope
SAK_1679	2	0.98	funct	0.82	funct	0.99	func	aspartyl/glutamyl-tRNA amidotransferase subunit B	Protein synthesis
SAK_1680	2	0.76	funct	0.82	funct	1.07	func	aspartyl/glutamyl-tRNA amidotransferase subunit A	Protein synthesis
SAK_1681	2	1.06	funct	1.01	funct	1	func	aspartyl/glutamyl-tRNA amidotransferase subunit C	Protein synthesis
SAK_1682	1	3.46	funct	3.1	funct	1.1	func	pyruvate phosphate dikinase	Energy metabolism
SAK_1683	3	2.47	funct	2.04	funct	1.35	func	hypothetical protein	conserved hypothetical protein
SAK_1684	2	2.06	funct	1.94	funct	1.47	func	CBS domain-containing protein	Unknown function
SAK_1685	1	0.82	funct	0.64	funct	1.46	func	3-hydroxybutyryl-CoA dehydrogenase	Fatty acid and phospholipid metabolism
SAK_1686	3	1.62	funct	0.79	pseudo	0.75	func	isochorismatase family protein	Unknown function
SAK_1687	5	1.99	funct	1.14	funct	0.81	func	transcriptional repressor CodY	Regulatory functions
SAK_1688	1	1.57	funct	0.53	funct	0.91	func	aminotransferase AlaT	Unknown function
SAK_1689	4	8.17	funct	1.08	funct	0.28	func	hypothetical protein	Cellular processes
SAK_1690	1	0.97	funct	1.53	pseudo	1.09	func	Cof-like hydrolase family protein	Unknown function
SAK_1691	2	1.08	funct	1.33	funct	0.89	func	asparaginase family protein	Energy metabolism
SAK_1692	2	2.06	funct	1.46	funct	1.16	func	shikimate 5-dehydrogenase	Amino acid biosynthesis
SAK_1693	2	2.73	funct	1.49	funct	1.27	func	aldo/keto reductase family oxidoreductase	Unknown function
SAK_1694	1	0.95	funct	0.48	funct	0.83	func	ATP-dependent DNA helicase RecG	DNA metabolism
SAK_1695	3	0.48	funct	0.29	funct	0.89	func	immunogenic secreted protein, putative	Cell envelope
SAK_1696	2	0.84	funct	1.17	funct	2.03	func	alanine racemase	Cell envelope
SAK_1697	3	0.71	funct	1.11	funct	2.33	func	4'-phosphopantetheinyl transferase	Fatty acid and phospholipid metabolism
SAK_1698	1	0.61	funct	1.37	funct	2.19	func	phospho-2-dehydro-3-deoxyheptonate aldolase	Amino acid biosynthesis
SAK_1699	1	0.64	funct	1.2	funct	1.05	func	preprotein translocase subunit SecA	Protein fate
SAK_1700	2	0.7	funct	1.01	funct	0.7	func	mannose-6-phosphate isomerase, class I	Energy metabolism
SAK_1701	3	0.59	funct	3.52	funct	0.48	func	fructokinase	Energy metabolism
SAK_1702	6	0.46	funct	3.21	funct	0.22	func	PTS system, sucrose-specific IIABC component	Signal transduction
SAK_1703	2	0.88	funct	1.35	funct	0.77	func	sucrose-6-phosphate hydrolase	Energy metabolism

SAK_1705	2	1.39	funct	1.34	funct	1.15	func	transcription antitermination protein NusB	Transcription
SAK_1706	4	1.09	funct	1.19	funct	1.39	func	hypothetical protein	conserved hypothetical protein
SAK_1707	1	1.31	funct	1.51	funct	1.14	func	elongation factor P	Protein synthesis
SAK_1712	2	1.13	funct	1.04	funct	1.67	func	hypothetical protein	hypothetical protein
SAK_1713	3	1.7	funct	1.16	funct	0.99	func	cytidine/deoxycytidylate deaminase family protein	Unknown function
SAK_1715	2	0.44	funct	0.15	funct	0.8	func	hypothetical protein	conserved hypothetical protein
SAK_1716	7	1.16	pseudo	0.36	funct	0.75	func	drug:H+ antiporter-2 (DHA2) family protein	Transport and binding proteins
SAK_1717	2	1.06	funct	0.93	funct	0.84	func	excinuclease ABC subunit A	DNA metabolism
SAK_1718	4	0.71	funct	0.99	funct	0.87	func	hypothetical protein	conserved hypothetical protein
SAK_1719	5	0.77	funct	0.85	funct	0.84	func	CorA metal ion transporter (MIT) family protein	Transport and binding proteins
SAK_1720	2	0.44	funct	1.33	funct	0.55	func	30S ribosomal protein S18	Protein synthesis
SAK_1721	2	0.35	funct	0.79	funct	0.67	func	single-stranded DNA-binding protein	DNA metabolism
SAK_1722	2	0.34	funct	0.8	funct	0.63	func	30S ribosomal protein S6	Protein synthesis
SAK_1723	3	1.46	funct	1.32	funct	0.91	func	A/G-specific adenine glycosylase	DNA metabolism
SAK_1724	1	1.77	funct	1.35	funct	1.06	func	DNA-binding protein	Unknown function
SAK_1725	2	2.13	funct	0.63	funct	0.96	func	thioredoxin	Energy metabolism
SAK_1727	5	1.06	funct	1.09	funct	1.1	func	MutS2 family protein	DNA metabolism
SAK_1728	2	1.08	funct	1.78	funct	0.78	func	hypothetical protein	Unknown function
SAK_1729	3	1.2	funct	1.79	funct	0.91	func	hypothetical protein	conserved hypothetical protein
SAK_1730	1	0.86	funct	1.52	funct	1.67	func	ribonuclease HIII	Transcription
SAK_1731	1	0.71	funct	1.42	funct	1.55	func	signal peptidase I	Protein fate
SAK_1732	1	0.48	funct	0.82	funct	2.15	func	RecD/TraA family helicase	Unknown function
SAK_1733	1	1.52	funct	2.85	funct	0.97	func	hypothetical protein	conserved hypothetical protein
SAK_1734	2	4.14	pseudo	1.6	funct	1	func	DNA polymerase IV	DNA metabolism
SAK_1735	2	0.37	funct	0.33	funct	0.16	func	formate acetyltransferase 1	Energy metabolism
SAK_1737	1	0.99	funct	0.52	funct	0.85	func	hypothetical protein	Cellular processes
SAK_1738	2	1.04	funct	0.48	funct	0.79	func	hypothetical protein	conserved hypothetical protein
SAK_1740	2	1.16	funct	0.51	funct	0.47	func	glycerol facilitator/aquaporin	Transport and binding proteins
SAK_1741	2	2.14	funct	0.31	funct	0.75	func	universal stress family protein	Cellular processes
SAK_1742	1	1.05	funct	0.14	funct	0.83	func	major facilitator family protein	Transport and binding proteins
SAK_1743	1	1.55	funct	0.95	funct	1.17	func	transcriptional regulator, putative	Regulatory functions
SAK_1744	4	1.38	funct	0.72	pseudo	1.86	func	x-prolyl-dipeptidyl aminopeptidase	Protein fate
SAK_1745	2	1.29	funct	0.81	funct	1.83	func	hypothetical protein	hypothetical protein
SAK_1746	3	1.16	funct	0.23	funct	1	func	polyprenyl synthetase family protein	Biosynthesis of cofactors, prosthetic groups, and carriers
SAK_1747	2	0.38	funct	0.36	funct	1.27	func	ABC transporter, ATP-binding protein CydC	Transport and binding proteins
SAK_1748	2	0.39	funct	0.3	funct	1.09	func	ABC transporter, ATP-binding protein CydD	Transport and binding proteins
SAK_1749	3	0.46	funct	0.18	funct	0.66	func	cytochrome d ubiquinol oxidase, subunit II	Energy metabolism
SAK_1750	1	0.36	funct	0.12	funct	0.57	func	cytochrome d ubiquinol oxidase, subunit II	Energy metabolism
SAK_1751	2	0.28	funct	0.07	funct	0.67	func	pyridine nucleotide-disulphide oxidoreductase family protein	Unknown function
SAK_1767	2	0.94	funct	0.48	funct	1.43	func	PhzF family phenazine biosynthesis protein	Cellular processes
SAK_1768	2	1.14	funct	0.38	funct	1.1	pseudo	acetyltransferase	Unknown function
SAK_1770	2	0.27	funct	0.36	funct	1.07	func	hypothetical protein	conserved hypothetical protein TIGR00103
SAK_1771	2	1.17	pseudo	2.41	funct	1.39	func	cyclopropane-fatty-acyl-phospholipid synthase	Fatty acid and phospholipid metabolism
SAK_1772	3	1.4	funct	1.48	pseudo	0.95	func	MerR family transcriptional regulator	Regulatory functions
SAK_1773	1	1.29	funct	1.21	funct	1.09	func	DNA polymerase III subunit epsilon	Unknown function
SAK_1774	5	0.95	funct	3.23	funct	1.31	func	hypothetical protein	conserved hypothetical protein
SAK_1775	3	0.75	funct	0.6	funct	0.73	func	hypothetical protein	conserved hypothetical protein TIGR00275
SAK_1776	4	1.44	funct	2.08	funct	0.91	func	hypothetical protein	conserved hypothetical protein
SAK_1777	3	0.45	funct	2.38	funct	0.32	func	30S ribosomal protein S14	Protein synthesis
SAK_1778	1	0.84	funct	0.65	funct	0.51	func	hypothetical protein	Energy metabolism
SAK_1779	3	0.92	funct	1.07	funct	0.83	func	putative DNA-binding/iron metalloprotein/AP endonuclease	Protein fate
SAK_1780	2	0.88	funct	0.95	funct	0.8	func	ribosomal-protein-alanine acetyltransferase	Protein synthesis
SAK_1781	2	1.11	funct	0.94	funct	0.81	func	M22 peptidase	Protein fate
SAK_1782	3	1.66	funct	1.76	funct	1.12	func	hypothetical protein	conserved hypothetical protein
SAK_1783	2	1.04	funct	1.4	funct	1.02	func	metallo-beta-lactamase family protein	Unknown function
SAK_1784	3	2.8	funct	2.18	funct	1.09	func	CHAP domain-containing protein	Unknown function
SAK_1785	3	1.35	funct	1	funct	1.03	func	glutamine synthetase, type I	Amino acid biosynthesis
SAK_1786	3	1.47	funct	0.95	funct	1.01	func	transcriptional regulator GlnR	Regulatory functions
SAK_1787	2	2.32	funct	0.81	funct	0.79	func	hypothetical protein	conserved hypothetical protein
SAK_1788	5	0.85	funct	0.4	funct	0.93	func	phosphoglycerate kinase	Energy metabolism
SAK_1789	1	2.81	funct	1.45	funct	1.35	func	5'-nucleotidase	Transport and binding proteins
SAK_1790	4	1.54	funct	0.74	funct	0.94	func	glyceraldehyde-3-phosphate dehydrogenase	Energy metabolism
SAK_1791	4	0.87	funct	1	funct	0.73	func	elongation factor G	Protein synthesis
SAK_1792	3	0.49	funct	1.25	funct	0.68	func	30S ribosomal protein S7	Protein synthesis
SAK_1793	3	0.5	funct	1.09	funct	0.72	func	30S ribosomal protein S12	Protein synthesis
SAK_1794	3	1.44	funct	1.81	funct	1.2	func	pur operon repressor	Regulatory functions
SAK_1795	1	1.12	funct	1.14	funct	1.47	func	HD domain-containing protein	Unknown function
SAK_1796	3	1.33	funct	1.17	funct	1.53	func	RmuC domain-containing protein	Unknown function
SAK_1797	1	1.4	funct	1.39	funct	1.54	func	hypothetical protein	Biosynthesis of cofactors, prosthetic groups, and carriers
SAK_1798	2	1.1	funct	1.41	funct	1.13	func	ribulose-phosphate 3-epimerase	Energy metabolism
SAK_1800	1	0.7	funct	0.87	funct	1.17	func	rRNA (guanine-N1-) methyltransferase (mycinamicin-resistance protein)	Protein synthesis
SAK_1801	1	0.72	funct	0.9	funct	1.18	func	dimethyladenosine transferase	Protein synthesis
SAK_1803	1	0.77	funct	0.73	funct	0.99	func	primase-related protein	Unknown function
SAK_1804	3	0.97	funct	0.79	funct	0.97	func	TatD family hydrolase	Unknown function
SAK_1805	1	2.44	funct	2.48	funct	1.77	func	hypothetical protein	hypothetical protein
SAK_1807	4	1.72	funct	2.13	pseudo	2.11	func	hypothetical protein	conserved hypothetical protein
SAK_1808	2	1.87	funct	2.38	funct	2	func	hypothetical protein	conserved hypothetical protein
SAK_1809	1	0.81	funct	1.11	funct	1.46	func	dltD protein	Cell envelope
SAK_1810	2	1.1	funct	1.07	funct	1.54	func	D-alanine--poly(phosphoribitol) ligase subunit 2	Cell envelope
SAK_1811	3	0.94	funct	1.13	funct	1.39	func	dltB protein	Cell envelope

SAK_1812	4	1.08	funct	1.72	funct	1.74	func	D-alanine--poly(phosphoribitol) ligase subunit 1	Cell envelope
SAK_1813	4	0.78	pseudo	1.19	funct	1.48	func	sensor histidine kinase DltS	Signal transduction
SAK_1814	3	0.77	funct	0.92	pseudo	1.26	func	DNA-binding response regulator DltR	Signal transduction
SAK_1815	2	0.71	funct	1.23	funct	1.27	func	50S ribosomal protein L34	Protein synthesis
SAK_1816	1	0.56	funct	1.52	funct	0.67	func	hypothetical protein	Cell envelope
SAK_1817	2	0.31	funct	0.12	funct	1.16	func	glycine betaine/proline ABC transporter, permease/substrate-binding protein	Transport and binding proteins
SAK_1818	3	0.18	funct	0.07	funct	1.94	func	glycine betaine/proline ABC transporter, ATP-binding protein	Transport and binding proteins
SAK_1820	2	1.35	funct	1.87	funct	0.47	func	putative L-ascorbate 6-phosphate lactonase	conserved hypothetical protein
SAK_1821	2	1.3	funct	2.7	funct	0.4	func	PRD domain-containing protein	Unknown function
SAK_1826	1	0.63	funct	0.96	pseudo	0.35	func	D-isomer specific 2-hydroxyacid dehydrogenase family protein	Unknown function
SAK_1828	1	1.36	funct	1.14	funct	0.73	func	LacI family sugar-binding transcriptional regulator	Regulatory functions
SAK_1830	1	1.3	funct	2.68	funct	0.8	func	L-ribulose-5-phosphate 4-epimerase	Energy metabolism
SAK_1835	2	1.11	funct	3.87	funct	0.78	func	ascorbate-specific PTS system enzyme IIC	Signal transduction
SAK_1836	1	1.38	funct	2.02	funct	0.82	func	hypothetical protein	conserved hypothetical protein
SAK_1837	1	1.48	funct	3.41	funct	0.99	func	hypothetical protein	hypothetical protein
SAK_1839	4	0.35	funct	0.15	pseudo	0.99	func	pfoR protein	Unknown function
SAK_1841	2	0.33	funct	2.52	funct	1.35	func	bifunctional glutamate--cysteine ligase/glutathione synthetase	Biosynthesis of cofactors, prosthetic groups, and carriers
SAK_1842	3	1.24	funct	0.79	funct	1.58	func	hypothetical protein	conserved hypothetical protein
SAK_1843	2	1.42	funct	0.66	funct	1.08	func	hypothetical protein	Cellular processes
SAK_1844	3	0.65	funct	0.89	funct	0.83	func	Hsp33-like chaperonin	Protein fate
SAK_1845	1	0.5	funct	0.93	funct	0.8	func	tRNA-dihydrouridine synthase family protein	Protein synthesis
SAK_1846	2	0.52	funct	0.96	funct	0.85	func	deoxynucleoside kinase family protein	Purines, pyrimidines, nucleosides, and nucleotides
SAK_1848	2	1.43	funct	0.8	funct	0.99	func	ATP-dependent Clp protease, ATP-binding subunit ClpC	Protein fate
SAK_1849	2	1.42	funct	1.03	funct	1.03	func	transcriptional regulator CtsR	Regulatory functions
SAK_1850	3	0.62	funct	0.68	funct	0.72	func	hypothetical protein	conserved hypothetical protein
SAK_1851	4	0.41	funct	0.69	funct	0.97	func	elongation factor Ts	Protein synthesis
SAK_1852	1	0.62	funct	1.11	funct	1.17	func	30S ribosomal protein S2	Protein synthesis
SAK_1853	3	0.98	funct	0.19	funct	1.04	func	alkyl hydroperoxide reductase, subunit C	Cellular processes
SAK_1854	1	1.17	funct	0.32	funct	1.33	func	NADH dehydrogenase	Energy metabolism
SAK_1870	2	1.22	funct	0.8	funct	0.73	func	mannosyl-glycoprotein endo-beta-N-acetylglucosamidase, putative	Cell envelope
SAK_1871	3	1.46	funct	0.98	funct	0.94	func	prolyl-tRNA synthetase	Protein synthesis
SAK_1872	3	1.67	funct	0.99	funct	0.85	func	M50A family peptidase	Protein fate
SAK_1873	2	1.57	funct	1.07	funct	0.99	func	phosphatidate cytidylyltransferase	Fatty acid and phospholipid metabolism
SAK_1874	4	1.8	funct	1.16	funct	0.9	func	undecaprenyl diphosphate synthase	Cell envelope
SAK_1875	2	1.43	funct	1.01	funct	0.81	func	preprotein translocase subunit YajC	Protein fate
SAK_1876	2	1.47	funct	0.89	funct	1.07	func	hypothetical protein	conserved hypothetical protein
SAK_1880	2	1.51	pseudo	1.58	pseudo	1.71	pseudo	sensor histidine kinase, putative	Signal transduction
SAK_1881	1	1.54	funct	1.26	funct	1.22	func	response regulator	Signal transduction
SAK_1882	3	2.88	funct	0.5	funct	0.39	func	UDP-glucose 4-epimerase	Energy metabolism
SAK_1883	3	4.35	pseudo	0.06	funct	0.1	func	glucan 1,6-alpha-glucosidase	Energy metabolism
SAK_1884	7	1.03	funct	0.47	funct	0.54	func	sugar ABC transporter, ATP-binding protein	Transport and binding proteins
SAK_1885	1	1.43	funct	1.05	funct	1	func	Fis family DNA-binding protein	Unknown function
SAK_1886	1	0.35	funct	1.13	funct	1.46	func	lactose operon protein LacX	Unknown function
SAK_1887	1	0.41	funct	0.97	funct	1.35	func	tagatose 1,6-diphosphate aldolase	Energy metabolism
SAK_1896	2	1.87	funct	6.32	funct	0.92	func	lactose phosphotransferase system repressor, putative	Regulatory functions
SAK_1899	2	0.96	funct	0.64	funct	0.77	func	D-tyrosyl-tRNA(Tyr) deacylase	Protein synthesis
SAK_1900	1	0.75	funct	0.47	funct	0.97	func	GTP pyrophosphokinase	Cellular processes
SAK_1901	1	0.41	pseudo	6.03	pseudo	0.57	func	bifunctional 2',3'-cyclic nucleotide 2'-phosphodiesterase/3'-nucleotidase precursor protein	Purines, pyrimidines, nucleosides, and nucleotides
SAK_1902	4	1.11	funct	1.16	funct	1.35	func	flavoprotein NrdI	Purines, pyrimidines, nucleosides, and nucleotides
SAK_1903	3	1.03	pseudo	0.84	funct	1.23	func	M42 family peptidase	Protein fate
SAK_1904	1	0.75	funct	0.9	funct	1.48	func	hypothetical protein	conserved hypothetical protein
SAK_1906	2	1.62	funct	1.19	funct	1.24	func	AraC family DNA-binding response regulator	Signal transduction
SAK_1907	1	1.87	funct	1.2	funct	1.36	func	hypothetical protein	Signal transduction
SAK_1908	1	7.38	funct	0.58	funct	1	func	PTS system mannose/fructose/sorbose family IIDsubunit	Signal transduction
SAK_1909	2	9.32	funct	0.42	funct	1.09	func	PTS system mannose/fructose/sorbose family IIC subunit	Signal transduction
SAK_1910	2	6.92	funct	0.52	funct	1.12	func	PTS system mannose/fructose/sorbose family IIB subunit	Signal transduction
SAK_1911	1	5.63	funct	0.72	funct	1.16	func	PTS system mannose/fructose/sorbose family IIA subunit	Signal transduction
SAK_1913	1	2.88	pseudo	3.31	pseudo	1.27	func	hypothetical protein	Cell envelope
SAK_1917	2	0.79	funct	3.64	funct	1.25	func	histidine kinase	Signal transduction
SAK_1919	5	0.39	funct	0.13	funct	0.13	func	hypothetical protein	Unknown function
SAK_1920	4	0.37	funct	0.08	funct	0.1	func	PTS system, glucose-specific IIABC component, putative	Signal transduction
SAK_1921	5	1.02	pseudo	1.11	pseudo	1.11	func	sensor histidine kinase	Signal transduction
SAK_1922	2	1.61	funct	0.91	funct	1.01	func	phosphate regulon transcriptional regulatory protein PhoB, putative	Signal transduction
SAK_1923	2	1.61	funct	0.96	pseudo	1.01	func	phosphate transport system regulatory protein PhoU	Transport and binding proteins
SAK_1928	2	0.95	pseudo	1.21	pseudo	0.9	func	hypothetical protein	conserved hypothetical protein
SAK_1930	1	0.9	funct	0.94	funct	1	func	ribosomal protein L11 methyltransferase	Protein synthesis
SAK_1931	3	0.99	funct	0.89	funct	0.96	func	hypothetical protein	conserved hypothetical protein
SAK_1932	2	7.1	funct	0.7	funct	0.89	func	MerR family transcriptional regulator	Regulatory functions
SAK_1933	3	1.53	pseudo	1.13	funct	0.81	func	acetyltransferase	Unknown function
SAK_1934	1	1.54	funct	1.12	funct	0.78	func	NUDIX family hydrolase	Unknown function
SAK_1937	1	1.22	funct	1.36	funct	0.64	func	acetyltransferase	Unknown function

SAK_1938	2	1.26	funct	1.61	funct	0.71	func	recombination factor protein RarA	Unknown function
SAK_1967	1	0.26	funct	0.29	funct	2.64	func	aminoglycoside 6-adenylyltransferase, putative	Cellular processes
SAK_1968	1	0.4	funct	0.35	funct	1.83	func	hypothetical protein	conserved domain protein
SAK_1978	1	3.42	funct	2.55	funct	2.98	func	PAP2 family protein	Unknown function
SAK_1981	2	0.73	funct	0.63	funct	0.9	func	CAAX amino terminal protease family protein	Unknown function
SAK_1982	1	0.43	funct	0.26	funct	2.16	func	rhodanese-like domain-containing protein	Unknown function
SAK_1983	2	0.47	pseudo	0.24	funct	2.21	func	cAMP factor	Cellular processes
SAK_1984	2	3.15	funct	2.19	funct	0.93	func	topology modulation protein	Regulatory functions
SAK_1985	2	2.66	funct	2.3	funct	0.69	func	glycerol dehydrogenase, putative	Central intermediary metabolism
SAK_1986	1	1	funct	2.44	funct	0.47	func	hypothetical protein	conserved hypothetical protein
SAK_1990	1	0.67	funct	0.83	funct	0.83	func	azaleucine resistance protein AzlC, putative	Cellular processes
SAK_1992	3	1.65	funct	0.51	funct	1	func	DNA-binding response regulator	Signal transduction
SAK_1993	1	1.86	funct	0.6	pseudo	1.05	func	sensor histidine kinase	Signal transduction
SAK_1994	3	1.61	funct	0.69	funct	1.29	func	hypothetical protein	hypothetical protein
SAK_1995	1	0.63	funct	1.01	funct	0.64	func	leucyl-tRNA synthetase	Protein synthesis
SAK_1996	1	0.76	funct	1.22	funct	0.42	func	major facilitator family protein	Transport and binding proteins
SAK_1997	2	1.39	funct	0.67	funct	0.94	func	hypothetical protein	Unknown function
SAK_1998	3	1	funct	0.76	funct	0.61	func	transcription antitermination protein NusG	Transcription
SAK_2003	1	1.21	funct	1.72	funct	0.96	func	preprotein translocase subunit SecE	Protein fate
SAK_2005	1	1	funct	0.62	funct	0.83	func	penicillin-binding protein 2A	Cell envelope
SAK_2006	1	0.65	funct	0.85	funct	0.68	func	ribosomal large subunit pseudouridine synthase A	Protein synthesis
SAK_2007	4	1.5	funct	3.92	funct	2.88	func	hypothetical protein	conserved hypothetical protein
SAK_2009	2	2.03	funct	1.6	funct	1.81	func	deoxyribose-phosphate aldolase	Purines, pyrimidines, nucleosides, and nucleotides
SAK_2010	4	2.37	funct	1.38	funct	1.47	func	concentrative nucleoside transporter (CNT) family protein	Transport and binding proteins
SAK_2011	3	2.59	funct	1.54	funct	1.8	func	uridine phosphorylase	Purines, pyrimidines, nucleosides, and nucleotides
SAK_2013	2	1.26	funct	1.11	funct	0.58	func	chaperonin GroEL	Protein fate
SAK_2014	3	1.38	funct	1.02	funct	0.45	func	co-chaperonin GroES	Protein fate
SAK_2015	4	0.72	funct	9.45	funct	0.64	func	ABC transporter, ATP-binding protein	Transport and binding proteins
SAK_2016	4	0.76	funct	7.55	funct	0.74	func	ABC transporter, permease protein	Transport and binding proteins
SAK_2017	1	0.9	funct	7.88	funct	0.99	func	ABC transporter, substrate-binding protein	Transport and binding proteins
SAK_2018	2	2.78	funct	2.44	funct	0.77	func	Cof-like hydrolase family protein	Unknown function
SAK_2019	1	15.39	funct	45.96	funct	1.39	func	glyoxalase family protein	Unknown function
SAK_2020	2	4.29	funct	16.53	funct	1.33	func	hypothetical protein	conserved hypothetical protein
SAK_2021	2	0.93	funct	0.74	funct	1.27	func	anaerobic ribonucleoside-triphosphate reductase activating protein	Purines, pyrimidines, nucleosides, and nucleotides
SAK_2022	1	0.62	funct	0.49	funct	1.22	func	acetyltransferase	Unknown function
SAK_2023	1	0.84	pseudo	0.4	funct	0.9	func	Gfo/Idh/MocA family oxidoreductase	Unknown function
SAK_2024	3	0.63	pseudo	0.43	funct	1.03	func	hypothetical protein	conserved hypothetical protein
SAK_2025	2	1.64	funct	1.05	funct	0.6	func	anaerobic ribonucleoside triphosphate reductase	Purines, pyrimidines, nucleosides, and nucleotides
SAK_2026	2	1.45	funct	1.87	funct	1.01	func	hypothetical protein	Cell envelope
SAK_2028	3	0.96	funct	0.83	funct	1.27	func	hypothetical protein	conserved hypothetical protein
SAK_2029	3	0.88	funct	0.71	funct	1.6	func	Holliday junction resolvase-like protein	conserved hypothetical protein TIGR00250
SAK_2030	3	1.01	funct	0.66	funct	1.63	func	hypothetical protein	conserved hypothetical protein
SAK_2031	1	2.8	funct	2.02	funct	1.38	func	transcriptional regulator Spx	Regulatory functions
SAK_2032	3	0.79	funct	0.97	funct	0.74	func	recombinase A	DNA metabolism
SAK_2033	2	0.61	funct	0.95	funct	1.05	func	competence damage-inducible protein A	DNA metabolism
SAK_2034	3	1.4	funct	1.27	funct	1.14	func	DNA-3-methyladenine glycosylase I	DNA metabolism
SAK_2035	3	1.57	funct	1.32	funct	1.4	func	Holliday junction DNA helicase RuvA	DNA metabolism
SAK_2036	2	1.54	funct	1.23	funct	1.24	func	major facilitator family transporter	Transport and binding proteins
SAK_2037	3	1.63	funct	1.36	funct	1.24	func	DNA mismatch repair protein	DNA metabolism
SAK_2039	3	1.12	funct	0.19	funct	0.59	func	cold shock protein	Cellular processes
SAK_2040	1	1.25	funct	0.6	funct	1.04	func	DNA mismatch repair protein MutS	DNA metabolism
SAK_2041	3	1.5	funct	0.42	funct	0.83	func	arginine repressor	Regulatory functions
SAK_2042	2	1	funct	0.84	funct	0.65	func	arginyl-tRNA synthetase	Protein synthesis
SAK_2043	2	3.24	funct	0.66	funct	0.81	func	hypothetical protein	conserved hypothetical protein
SAK_2044	3	0.83	funct	1.01	funct	1.1	func	hypothetical protein	conserved hypothetical protein
SAK_2045	3	0.83	funct	0.86	funct	0.88	func	hypothetical protein	Cell envelope
SAK_2046	2	0.81	funct	0.65	funct	0.94	func	aspartyl-tRNA synthetase	Protein synthesis
SAK_2047	1	0.68	funct	0.41	funct	0.98	func	histidyl-tRNA synthetase	Protein synthesis
SAK_2048	6	0.17	funct	0.74	funct	0.88	func	50S ribosomal protein L32	Protein synthesis
SAK_2049	4	0.17	funct	0.85	funct	0.71	func	50S ribosomal protein L33	Protein synthesis
SAK_2068	2	0.72	funct	0.03	pseudo	0.61	pseudo	glycine betaine/carnitine/choline ABC transporter, ATP-binding protein	Transport and binding proteins
SAK_2069	3	0.57	funct	0.06	pseudo	0.58	func	glycine betaine/carnitine/choline ABC transporter, permease/substrate-binding protein	Transport and binding proteins
SAK_2070	2	1.43	funct	0.79	funct	0.93	func	hypothetical protein	Cellular processes
SAK_2071	2	1.5	funct	0.7	funct	0.82	func	hypothetical protein	conserved hypothetical protein
SAK_2072	2	0.5	funct	1.76	funct	1.32	func	hypothetical protein	conserved hypothetical protein
SAK_2073	4	0.45	funct	1.29	funct	1.58	func	hypothetical protein	Cell envelope
SAK_2075	1	1.69	funct	0.57	funct	0.88	func	hypothetical protein	conserved hypothetical protein
SAK_2095	2	0.44	funct	1.5	funct	0.55	func	30S ribosomal protein S4	Protein synthesis
SAK_2096	2	1.13	funct	2.45	funct	1.25	func	hypothetical protein	conserved hypothetical protein
SAK_2097	2	1.03	funct	2.21	funct	1.44	func	replicative DNA helicase	DNA metabolism
SAK_2098	2	0.93	funct	2.16	funct	1.44	func	50S ribosomal protein L9	Protein synthesis
SAK_2099	3	1.93	funct	2.36	funct	1.15	func	DHH family protein	Unknown function
SAK_2100	1	0.88	funct	1.11	funct	1.34	func	tRNA uridine 5-carboxymethylaminomethyl modification enzyme GidA	Unknown function
SAK_2101	1	0.91	funct	0.81	pseudo	1.64	func	MarC membrane protein	Cell envelope
SAK_2102	1	0.78	funct	0.64	funct	1.44	func	tRNA-specific 2-thiouridylase MnmA	Protein synthesis
SAK_2103	1	0.73	funct	1.01	funct	0.79	func	L-serine dehydratase, iron-sulfur-dependent, beta subunit	Energy metabolism

SAK_2104	1	0.79	funct	0.79	funct	0.49	func	L-serine dehydratase, iron-sulfur-dependent, alpha subunit	Energy metabolism
SAK_2105	3	0.9	funct	4.56	funct	1.04	func	transglycosylase-like domain-containing protein	Unknown function
SAK_2106	3	2.02	funct	3	funct	1.07	func	LysM domain-containing protein	Unknown function
SAK_2107	1	1.57	funct	0.75	funct	0.86	func	cobalt ABC transporter, permease protein	Transport and binding proteins
SAK_2108	1	1.83	funct	0.87	funct	0.76	func	cobalt transporter ATP-binding subunit	Transport and binding proteins
SAK_2109	1	0.9	funct	1.3	funct	0.73	func	cobalt transporter ATP-binding subunit	Transport and binding proteins
SAK_2110	3	1.26	funct	1.04	funct	0.71	func	CDP-diacylglycerol--glycerol-3-phosphate 3-phosphatidyltransferase	Fatty acid and phospholipid metabolism
SAK_2111	1	0.87	funct	0.74	funct	1.22	func	M16B family peptidase	Protein fate
SAK_2114	2	1.18	funct	1.63	funct	2.34	func	recombination protein F	DNA metabolism
SAK_2115	2	0.39	funct	0.72	pseudo	0.9	func	glucose uptake protein	Transport and binding proteins
SAK_2116	4	1.57	funct	1.49	funct	1	func	DNA-binding protein	Unknown function
SAK_2117	2	0.62	funct	1.6	funct	1.22	func	inosine 5'-monophosphate dehydrogenase	Purines, pyrimidines, nucleosides, and nucleotides
SAK_2118	2	0.88	funct	0.44	funct	1.06	func	arginine repressor, putative	Regulatory functions
SAK_2119	3	0.82	funct	0.39	funct	1.08	func	Crp/FNR family transcriptional regulator	Regulatory functions
SAK_2120	1	0.21	pseudo	0.1	pseudo	0.53	func	B3/4 domain-containing protein	Unknown function
SAK_2121	3	0.97	funct	2.42	funct	0.18	func	arginine deiminase	Energy metabolism
SAK_2122	4	0.84	funct	2.38	funct	0.17	func	acetyltransferase	Unknown function
SAK_2123	3	0.98	funct	2.88	funct	0.18	func	ornithine carbamoyltransferase	Amino acid biosynthesis
SAK_2124	1	0.99	funct	3.64	funct	0.23	func	arginine/ornithine antiporter	Transport and binding proteins
SAK_2125	2	1.24	funct	3.38	funct	0.22	func	carbamate kinase	Energy metabolism
SAK_2129	1	0.56	funct	0.57	funct	1.15	func	ABC transporter, ATP-binding protein	Transport and binding proteins
SAK_2130	1	0.64	funct	0.84	funct	1.6	func	hypothetical protein	Cell envelope
SAK_2134	1	4.92	funct	6.74	funct	0.7	func	rRNA large subunit methyltransferase	conserved hypothetical protein
SAK_2135	2	1.13	funct	5.92	funct	2.07	func	serine peptidase HtrA	Protein fate
