# Supplementary material for: Reductive evolution in Streptococcus agalactiae and the emergence of a host adapted lineage
Source: BMC Genomics. 2013 Apr 15;14:252. doi: 10.1186/1471-2164-14-252 (PMC3637634; doi:10.1186/1471-2164-14-252)
Supplement: Additional file 8: Table S8 — Provides the characteristics of the Illumina reads and of contigs generated by Velvet. [file 1471-2164-14-252-S8.pdf]

Table S8: Characteristics of the Illumina Reads and of contigs generated by Velvet.

	SS1218	90-503	SS1219	05-108A	CF01173	SS1014
nb of reads	17,506,655	7,105,795	6,980,465	13,362,738	17,037,136	16,757,467
size of reads	36	34	34	36	36	36
reads used to generate the contigs	17,017,388	6,735,673	6,688,552	12,680,839	16,577,819	16,298,314
nb of velvet contigs	116	120	125	137	270	204
nb of velvet contigs>200 nt	76	84	78	87	141	110
median coverage depth	125.7	41.3	41.1	89.6	107.5	107.2
n50	43177	40750	45794	36697	35307	38123
